# Reviving the Dead: History and Reactivation of an Extinct L1

**DOI:** 10.1371/journal.pgen.1004395

**Published:** 2014-06-26

**Authors:** Lei Yang, John Brunsfeld, LuAnn Scott, Holly Wichman

**Affiliations:** 1 Department of Biological Sciences, University of Idaho, Moscow, Idaho, United States of America; 2 Institute for Bioinformatics and Evolutionary Studies, University of Idaho, Moscow, Idaho, United States of America; University of Utah School of Medicine, United States of America

## Abstract

Although L1 sequences are present in the genomes of all placental mammals and marsupials examined to date, their activity was lost in the megabat family, Pteropodidae, ∼24 million years ago. To examine the characteristics of L1s prior to their extinction, we analyzed the evolutionary history of L1s in the genome of a megabat, *Pteropus vampyrus*, and found a pattern of periodic L1 expansion and quiescence. In contrast to the well-characterized L1s in human and mouse, megabat genomes have accommodated two or more simultaneously active L1 families throughout their evolutionary history, and major peaks of L1 deposition into the genome always involved multiple families. We compared the consensus sequences of the two major megabat L1 families at the time of their extinction to consensus L1s of a variety of mammalian species. Megabat L1s are comparable to the other mammalian L1s in terms of adenosine content and conserved amino acids in the open reading frames (ORFs). However, the intergenic region (IGR) of the reconstructed element from the more active family is dramatically longer than the IGR of well-characterized human and mouse L1s. We synthesized the reconstructed element from this L1 family and tested the ability of its components to support retrotransposition in a tissue culture assay. Both ORFs are capable of supporting retrotransposition, while the IGR is inhibitory to retrotransposition, especially when combined with either of the reconstructed ORFs. We dissected the inhibitory effect of the IGR by testing truncated and shuffled versions and found that length is a key factor, but not the only one affecting inhibition of retrotransposition. Although the IGR is inhibitory to retrotransposition, this inhibition does not account for the extinction of L1s in megabats. Overall, the evolution of the L1 sequence or the quiescence of L1 is unlikely the reason of L1 extinction.

## Author Summary

Most of a typical mammalian genome is occupied by transposable elements, which have played an important role in shaping these genomes, and L1s account for approximately half of this transposable element load. Mammals have evolved several mechanisms to control L1 retrotransposition, and yet L1s remain active in almost all mammalian lineages. However, L1s were found to have gone extinct in the megabat family ∼24 million years ago. We were able to trace megabat L1s to the ancestral L1 families shared by all mammals as well as identify bat-specific L1 families. Unlike most well-characterized mammals which have a single active L1 lineage, multiple L1 lineages have persisted in megabats throughout their evolutionary history. When the L1 extinction occurred in megabats, two active lineages lost their ability to retrotranspose almost simultaneously after a burst of activity. We synthesized the L1 from the most active family at the time of extinction and found a long intergenic spacer between its two protein coding genes. Tissue culture assays of the reconstructed megabat L1 revealed that both genes supported retrotransposition, but that the spacer is inhibitory. Despite the inhibition, this family accounted for 18% of the L1s detected in the megabat genome.

## Introduction

L1 (LINE-1, Long INterspersed Element-1) belongs to the superfamily of autonomously replicating, retrotransposable elements that lack long terminal repeats. Functional L1s are 6,000–7,000 bp long and made up of a 5′ untranslated region (5′UTR), two non-overlapping open reading frames (ORFs) known as ORF1 and ORF2, an intergenic region (IGR) usually less than 100 bp and a 3′UTR followed by a poly-adenosine sequence [1]. The proteins encoded by both ORFs are strictly required for L1 retrotransposition and have very strong *cis*-preference [2], [3]. The function of the IGR is less well characterized, but it is known to be indispensable for the translation of human ORF2 protein [4] and to serve as an internal ribosome entry site (IRES) in mice [5].

There is considerable evidence that transposable elements, including L1s, have significant effects on the genome. L1 retrotransposition is one of the major sources of mutagenesis and genome instability [6], [7]. Besides their copy-and-paste retrotransposition mechanism that interrupts genes and disrupts the normal splicing of messenger RNAs [8], L1s also cleave genomic DNA with the endonuclease they encode [9]–[13] and are sites of ectopic recombination due to their homology to each other and prevalence throughout the genome [14]–[18]. L1s and their dependents may be occasionally co-opted to provide host functions. For example, they may serve as the source of new genes [8] or structural chromosome components [19], or regulate genes in their vicinity by various mechanisms [20]–[22]. They have also been proposed to play a role in X chromosome inactivation [23]–[25], neuro-plasticity [26]–[28] and regulatory functions [29].

L1s have been coevolving with their mammalian host genomes since before the eutherians and metatherians diverged [30] more than 160 million years ago (MYA) [31]. The tempo of L1 retrotransposition can vary both between species and at different time intervals within species [32]–[35]. They evolve as master lineages such that closely related active L1 copies succeed the older masters and become new major contributors to the total retrotransposition events [33], [36]–[38]. Most species are dominated for long periods of time by a single such master lineage [1], although multiple lineages are occasionally active at the same time [32], [35], [39]. Retrotransposition of the L1 population is extremely inefficient and few new active elements are produced, with the vast majority of new inserts being 5′ truncated pseudogenes. There are over 500,000 copies of L1 in the human reference genome [40], but only 80–100 of the L1s in an average human genome are estimated to be full-length and retrotranspositionally competent, with just six of these contributing more than 80% of the total L1 activity. These six elements are closely related; all belong to the youngest family of human L1s, and four of them belong to the youngest clade within that family [41]. Because there is no known mechanism for precise excision of L1s from the genome, old elements accumulate and make up 15–20% of a typical mammalian genome [40], [42]. These ‘fossil’ sequences make it possible to track the activity of L1s within a particular mammalian clade back many millions of years.

One possible reason for this unusual pattern of L1 evolution is that L1s are epigenetically silenced [43], [44] and highly regulated by a set of host defense mechanisms [45]–[48], especially in germline cells. Given the strong host defenses controlling L1 activity, it might seem reasonable to expect L1 extinctions among mammalian lineages. To clarify the terms related to loss of L1 activity in this work, we refer to a period of low L1 activity as “quiescence” and complete loss of L1 activity as “extinction.” Indeed, quiescence or extinction of L1 has been proposed several times in the literature [32], [49]–[54], but few of these cases have been examined in a phylogenetic context to convincingly demonstrate that extinction, and not simply quiescence, best explains the lack of recent L1 insertions into the genome. Because L1s are transmitted vertically with no evidence of horizontal transmission among mammals, ancient L1 extinctions would affect all subsequent species and should be the most easily identified and confirmed. One well-documented case of L1 extinction occurred in the ancestor of the megabat family, Pteropodidae, which is the focus of this study. The L1 extinction was verified in 11 sampled genera within Pteropodidae, but did not affect other families of bats. The ancestor of the megabats had two active L1 lineages, both of which became extinct at about the same time at least 24 MYA [50].

In this study, the evolutionary history of L1s prior to their extinction in megabats was explored by data-mining the unassembled genome of *Pteropus vampyrus*, the first publicly available genome trace files of the megabat family. At the time of L1 extinction, *P. vampyrus* contained two active L1 lineages. We determined that these lineages likely diverged before the origin of bats. We reconstructed the master element of the more active lineage at the time of L1 extinction and compared its structure to other active L1s, noting particularly that the IGR between the two ORFs is dramatically longer than that of the well-characterized L1s of human and mouse. Finally, we created chimeric L1s between the reconstructed megabat L1 and a human L1 to test the ability of the extinct megabat L1 to support retrotransposition in tissue culture and we manipulated the IGR to explore its effect on retrotransposition.

## Results

To be clear about nomenclature used in this paper, we refer to clades of closely related L1s identified by shared, co-segregating sites as *subfamilies*. Closely related subfamilies are grouped into *families* that represent a window of L1 deposition into the genome. These families replace each other sequentially within a clade to form a *lineage*.

### Evolutionary history of L1 in megabats

**Figure 1:**
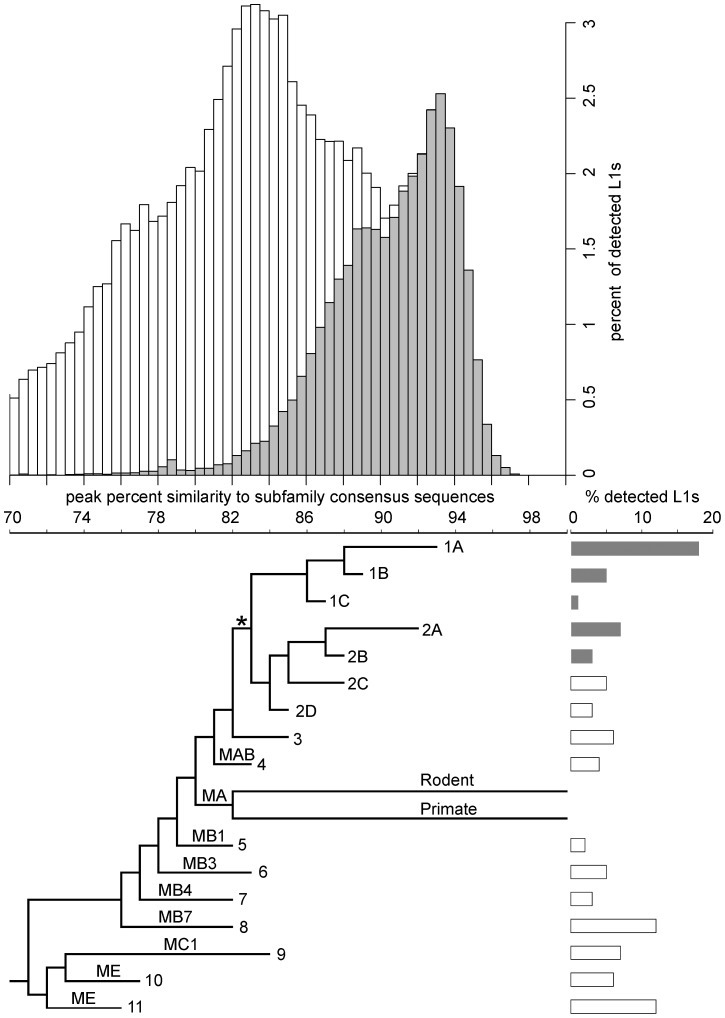
Age distribution and phylogeny of L1s in the megabat genome. The histogram shows the age distribution of megabat L1s as percent of the total 79,978 L1s detected in the megabat genome. Grey bars indicate L1s that are bat-specific. Age of L1s is determined by their percent identity to the corresponding subfamily consensus in 0.5% windows on the horizontal axis – the higher the percent identity, the younger the subfamily. The horizontal axis is shared with the phylogenetic tree which shows the evolutionary history of L1 families. Taxa names are the numbers assigned to megabat L1 families; names on branches are those given to ancestral mammalian L1 families by Smit *et al.* [58]. Divergence of the human- and rodent-specific L1s and their persistence to present time are indicated by labeled branches. The backbone of the tree is derived from the maximum likelihood tree of all megabat L1 subfamilies and ancestral mammalian L1 families shown in Figure S1, and the branch lengths of the tree were calibrated at the peak of retrotransposition of each family as described in Materials and Methods. * indicates the point after which bat-specific L1s (grey bars) diverged. Lengths of the bars to the right of each terminal branch indicate the percent of all detected L1s contributed by that family.

To investigate the history of L1 retrotransposition in the megabats, we identified subfamilies using COSEG in RepeatMasker [55] based on shared, co-segregating sites within 575 bp of the 3′ end of ORF2. These were designated subfamilies 0–63 using the convention of the program. The consensus sequences of these subfamilies were subjected to phylogenetic analysis and the phylogenetic relationships were used to identify families with the stipulation that the pairwise distances between subfamilies within a family be no greater than 3.5%. This distance was based on the observed phylogenetic clustering of subfamily consensus sequences. Given that the L1 masters are constantly being replaced during evolution, perfect designation within large families is not possible. The 3.5% threshold was chosen according to practical observations to cluster closely related subfamilies without inflating the number of families. This method identified 16 L1 families that account for the peaks of L1 fixation in the megabat genome (Figure 1 and Table S1).

Previous work indicated that two major lineages of L1 were active at the time of L1 extinction in megabats [50]. Full-length consensus sequences from two time points in the evolution of each lineage can be found in RepBase [56], [57], designated L1-1_PVa to L1-4_PVa. COSEG analysis confirms and extends this history. Lineage 1 corresponds to families 1A (L1-2_PVa), 1B (L1-3_PVa) and 1C. Lineage 2 corresponds to families 2A (L1-1_PVa), 2B (L1-4_PVa), 2C and 2D. It is clear that these two lineages existed prior to the emergence of the bats since families 2C and 2D are not bat-specific, but are closely related to elements found in various Laurasiatheria species. The older L1 families identified in our work (5–11) have high identity to the L1 families shared by all placental mammals [58] and by the Laurasiatheria superorder [59]. Smit *et al.* [58] designated the ancestral mammalian L1 families from most recent to oldest as L1MA, L1MB, L1MC, L1MD and L1ME. Subfamilies within each family are identified by number, with 1 being the most recent. The bottom panel of Figure 1 places megabat L1 dynamics in the context of these ancestral L1 families and the extant L1 lineages of primates and rodents. The relationship between the COSEG subfamilies, families and the ancestral L1s are summarized in Table S1.

### Tempo of L1 activity and extinction in megabats

To examine the activity and extinction of L1s in megabats, we extracted 79,978 L1 sequences from the ORF2 of L1s in the ∼2× unassembled shotgun sequence of the *P. vampyrus* genome (Baylor College of Medicine) and assigned them to one of the subfamilies described above based on sequence similarity. The age of each sequence was approximated by its percent identity to the subfamily consensus – the higher the percent identity, the younger the sequence. Subfamilies were combined into their designated families as determined by phylogenetic analysis (described above) and the age distribution was determined for each family. Taking all families together, we observed periodic fluctuations in the number of L1s fixed in the genome (Figure 1, top).

**Figure 2:**
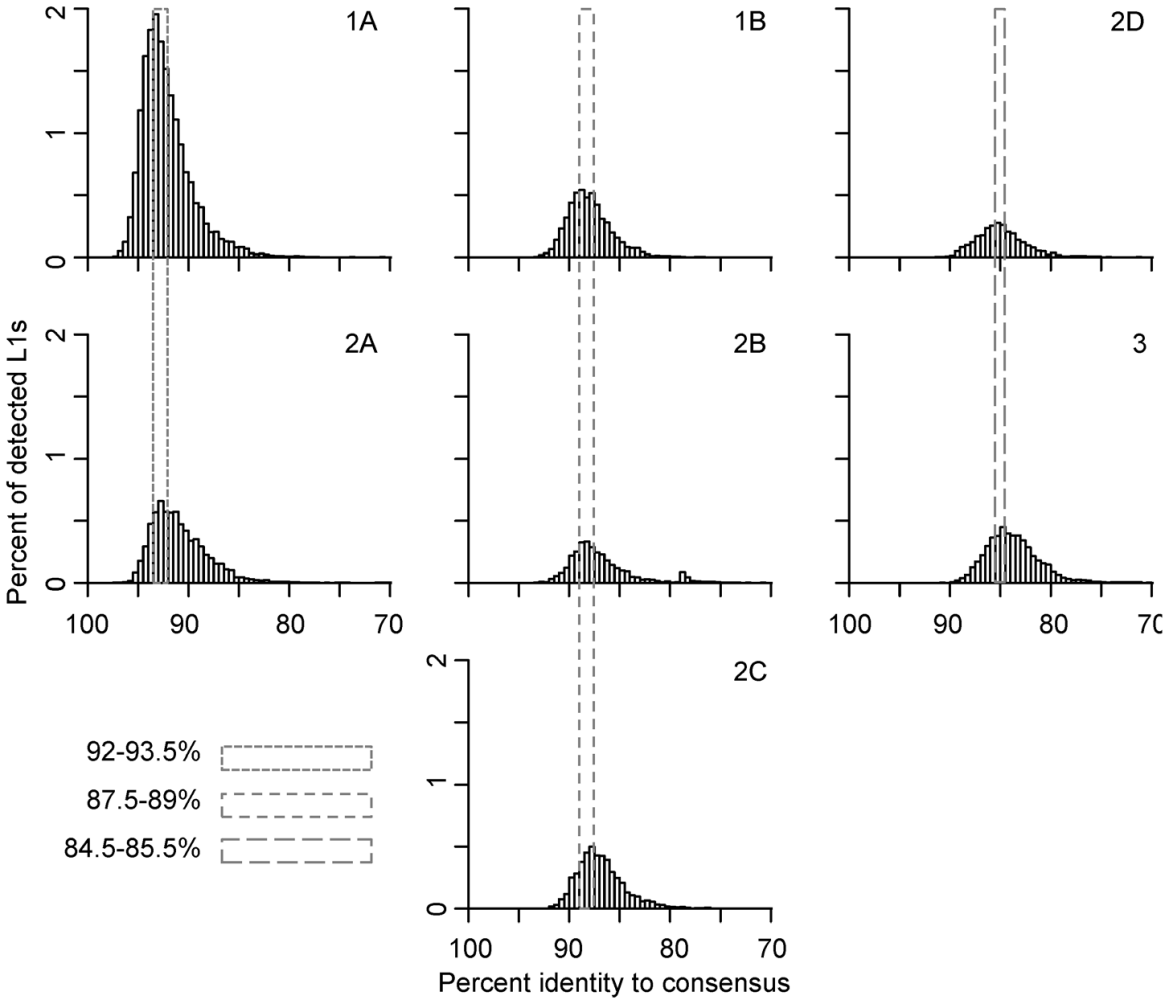
Persistence of concurrently active L1 families. Concurrent L1 families are arranged vertically. Names of families are noted on the top-right corner of each panel. L1 ages are determined by their percent identity to the corresponding subfamily consensus in 0.5% windows – the higher the percent identity, the younger the element. L1 copy numbers are normalized as percent of total detected L1s. The retrotransposition peaks of concurrent families are marked with dashed-line boxes; smaller dashes indicate younger families.

At least two large waves of L1 fixation in megabats can be identified in the lineages described above with peaks at 92–93.5% and 87.5–89% similarity to subfamily consensus sequences (Figure 2). Each peak corresponds to activity of two or more families and to multiple lineages. The most recent peak, accounting for 25% of the L1s detected in the megabat genome, corresponds to families 1A and 2A and is megabat-specific. No more recent waves of retrotransposition can be identified, consistent with the extinction of L1 retrotransposition in the common ancestor of megabats ∼24 MYA [50]. The next peak, accounting for 13% of detected L1s, corresponds to activity in families 1B, 2B and 2C. A third peak, accounting for 12% of detected L1s, resides at 84.5–85.5% and corresponds to families 2D and 3; this peak likely represents retrotransposition prior to the origin of bats. Older waves of L1 fixation are also evident and correspond to ancestral mammalian L1 families.

The dynamics of families within lineages 1 and 2 are not perfectly consistent with short bursts of retrotransposition followed by long periods of quiescence. Given the evolutionary pattern of L1 as master lineages, most L1 sequences evolve neutrally after their insertion into the genome. Therefore, the distribution of mutations in elements inserted at the same time should follow a Poisson distribution (*i.e.*, the mean divergence from the consensus is expected to be equal to the variance of the distribution). However, the mean of each family is 1–2% larger than the peak, indicating that the variance of the distribution is higher than that of a Poisson distribution. This increased variance could be due to sequence differences between active L1s in the same subfamily at the time of transposition, a wave of retrotransposition over an extended period of time, errors introduced during L1 retrotransposition, technical problems with the analysis, or some combination of these. Technical issues might include false detection by RepeatMasker, incorrect assignment of some elements to their lineage or combining small lineages with larger ones, for example. Interestingly, the highest copy number peak is for family 1A, one of the two youngest detectable lineages active just prior to L1 extinction. This peak accounts for 18% of the total L1s detected in the megabat genome.

### Reconstruction of an extinct L1

We sought to reconstruct a full-length version (minus the UTRs, which are difficult to accurately reconstruct) of the more active L1 lineage in megabats at the time of L1 extinction, synthesize it and test its activity in a tissue culture assay. It was not possible to reconstruct the less active lineage with confidence because the copy number, especially in the 5′ end, is too low. Since the extinction of megabat L1 retrotransposition happened in the common ancestor of the family, the retrotransposition history of L1 in *P. vampyrus* represents that of the whole Pteropodidae family.

Reconstruction was conducted on the *P. vampyrus* genome using a consensus-based method, with curated correction of CpG sites. We performed this reconstruction independently, without reference to RepBase [56], [57], thus the RepBase reconstruction served as a way to assess the quality of our reconstruction and a benchmark for problematic areas. Our reconstructed megabat L1 (GenBank accession number KF796623) has 99.7% identity to the RepBase reconstruction (RepBase Reports 10:(3), 474-474, 2010, available at http://www.girinst.org/2010/vol10/issue3/L1-2_PVa.html) at the nucleotide level, with six differences (two in ORF1 and four in ORF2) at the amino acid level. The amino acid differences were examined individually in the original alignments: three resulted from ambiguous nucleotides or frame shifts in the RepBase reconstruction, one from CpG site correction and two from variable sites which we called differently than RepBase. None of these differences were at sites of conserved amino acids (see below). Note that although RepBase designation L1-2_PVa suggests that this sequence falls within lineage 2, we follow the precedence of Cantrell *et al.* [50] to designate it as a member of lineage 1.

We compared the reconstructed L1 to the most recently active consensus sequences from 31 diverse mammalian species (Table S2 and Text S1 and S2). Sequences are taken from RepBase except five which we reconstructed from trace files, including a rodent species carrying dead L1s, *Oryzomys palustris*. As noted in the Materials and Methods, several sequences were edited to restore ORFs. These alterations were generally within A-rich tracts, which are common in L1s and difficult to reconstruct with confidence. Since the 5′ end of ORF1 can be non-homologous in different mammalian species [1], [60], we used only the conserved region of ORF1 (amino acids 123–321, bp 1273–1869 of L1rp, GenBank accession number AF148856) as well as the region corresponding to full-length ORF2 of L1rp (bp 1987–5814) for this comparison. The orthologous region of the reconstructed megabat ORF1 retains all the conserved amino acid sites, while the reconstructed ORF2 has two private changes (L418V and V671T, bp 3238–3240 and 3997–3999, respectively). These differences are consistent between our reconstruction and L1-2_PVa in RepBase and were verified in the original alignment to assure that they are not ambiguous in our reconstruction.

We investigated the adenosine content of the reconstructed terminal members of megabat lineages 1 and 2 and 31 additional L1 consensus sequences from the mammalian species listed in Table S2. L1 A-content of the two ORFs and the intergenic region (IGR) ranged from 39% to 44.5%, with a mean of 41.9%. Megabat L1 A-content was high among the species examined: lineage 1 ranked fifth at 43.7% and lineage 2 ranked second at 44.3%.

To our surprise, the length of the megabat L1 IGR set it apart from the well-characterized L1s of rodents and primates. The IGR lengths of the surveyed L1 sequences from 31 species are listed in Table S2 and range from 18 to 580 bp. At 445 bp, the IGR of the reconstructed L1 is dramatically longer than either the median (63 bp) or mean (172 bp) among the species examined. Long IGRs were found among marsupials, Laurasiatheria (which includes bats) and Afrotheria species, but not among Euarchontoglires. Long IGRs are found in megabat families 1A (445 bp) and 1B (481 bp), but the IGR length of families 2A (38 bp) and 2B (26 bp) is comparable to that of the majority of mammalian species. The IGR lengths in the remaining megabat L1 families are unknown. When multiple sequences were available in RepBase, we used the consensus of the most recently active L1 from each species for comparison; therefore, long IGRs could have existed in older or less active clades, or in sequences for which only partial reconstructions are feasible.

### Retrotransposition of the reconstructed L1

**Figure 3:**
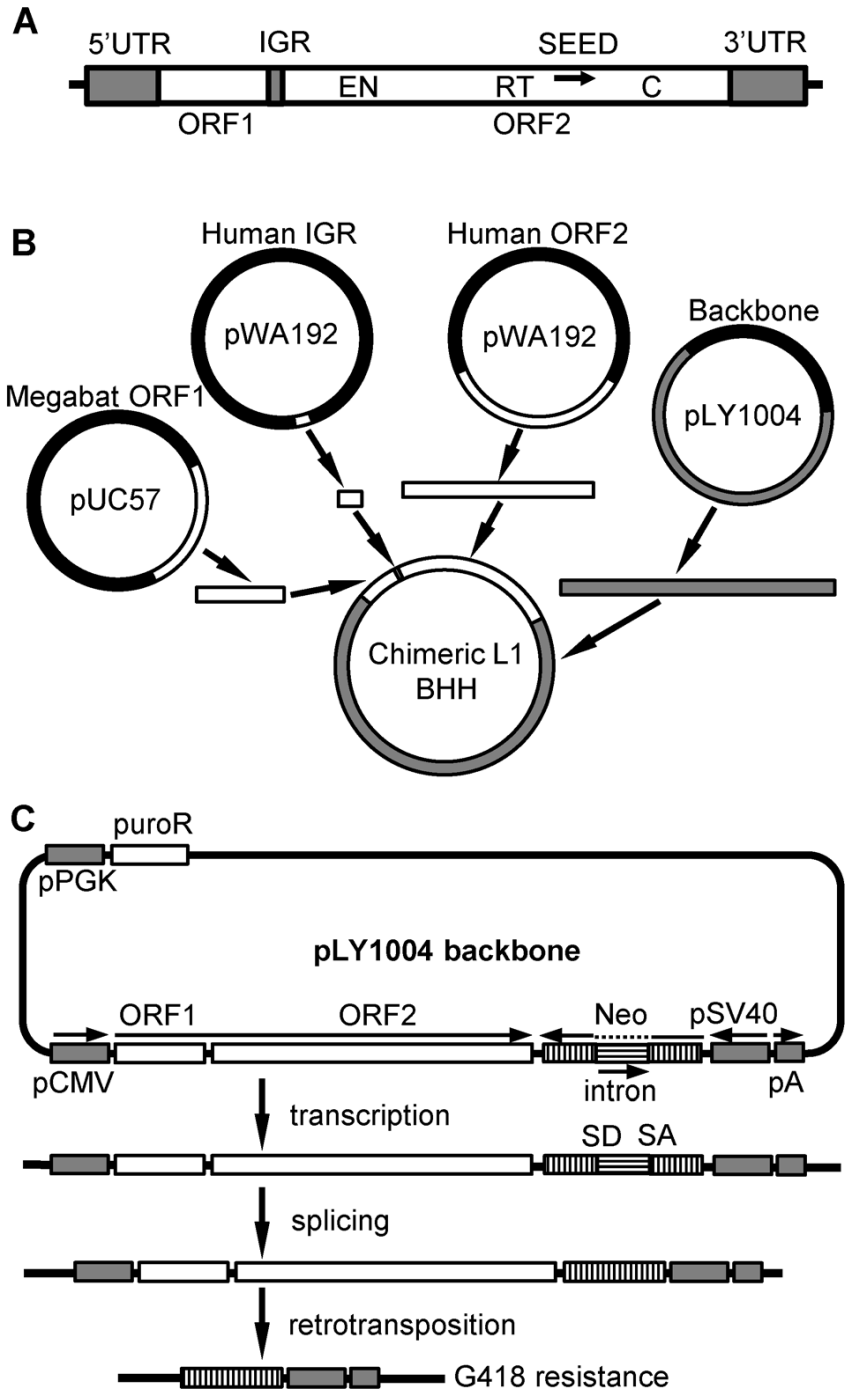
Scheme for assembly of chimeric L1 constructs. (A) Structure of a typical L1. UTR: untranslated region, ORF: open reading frame, IGR: intergenic region, EN: endonuclease motif, RT: reverse transcriptase motif, C: C-terminal domain, SEED: the region amplified by degenerate PCR (arrow) that served as the initial seed for reconstruction of the consensus sequence. (B) Chimeric L1 production. Human and megabat L1 segments were cloned separately into plasmids. L1 segments and the plasmid backbone with compatible overhangs were generated either by PCR or restriction enzyme digestion and joined together by a multi-way ligation. In this example ORF1 and the IGR are from megabat while ORF2 is from human (BBH). All eight combinations were produced in this manner. (C) Retrotransposition rate assay. The backbone of the constructs, linearized pLY1004, includes the puromycin resistance gene (*puro*R) driven by a constitutive promoter (pPGK), and an inverse neomycin resistance gene (*neo*) close to the cloning site for the L1. Puromycin resistance selects for cells that have acquired a L1 construct. Subsequently, neomycin resistance selects for cells that hosted retrotransposition events as follows. Transcription and subsequent retrotransposition of the cloned L1, driven by a pCMV promoter, trigger the splicing between donor (SD) and acceptor (SA) sites, activating the inverse-oriented *neo* cassette which is driven by an SV40 promoter. Thus, a cell will give rise to a colony if it accommodated a retrotransposition event and, thus, excision of the intron in *neo*, allowing it to survive G418 selection.

To ask whether the reconstructed megabat L1 is capable of supporting retrotransposition, we synthesized it and assessed its activity in a retrotransposition rate assay derived from the work of Moran *et al.* [61]. This assay is routinely used to measure retrotransposition rates of L1s in a tissue culture system [47], [62]–[64]. Reconstruction of fossil sequences can be challenging; even one error in reconstruction could block retrotransposition. Therefore, we synthesized the reconstructed gene in three segments and created all possible chimeric combinations using human L1rp [65]–[67] as a scaffold (Figure 3). Human L1rp is one of the most active natural human L1s characterized to date, and thus provides a robust background against which to test the effect of each L1 segment on retrotransposition rate. An independent L1rp construct, pWA192 [67], was used as a positive control. An ORF1 mutant of L1rp [68] cloned in the same genetic context as the chimeric L1s was used as a negative control. The chimeric L1s are named by the source of their ORFs and IGR – H for human L1rp or B for the reconstructed megabat L1. For example, HHH represents the two ORFs and IGR of L1rp (GenBank accession number AF148856), BBB represents the reconstructed megabat L1 (GenBank accession number KF796623) and HBH represents the chimeric L1 that includes human ORF1, megabat IGR and human ORF2.

**Figure 4:**
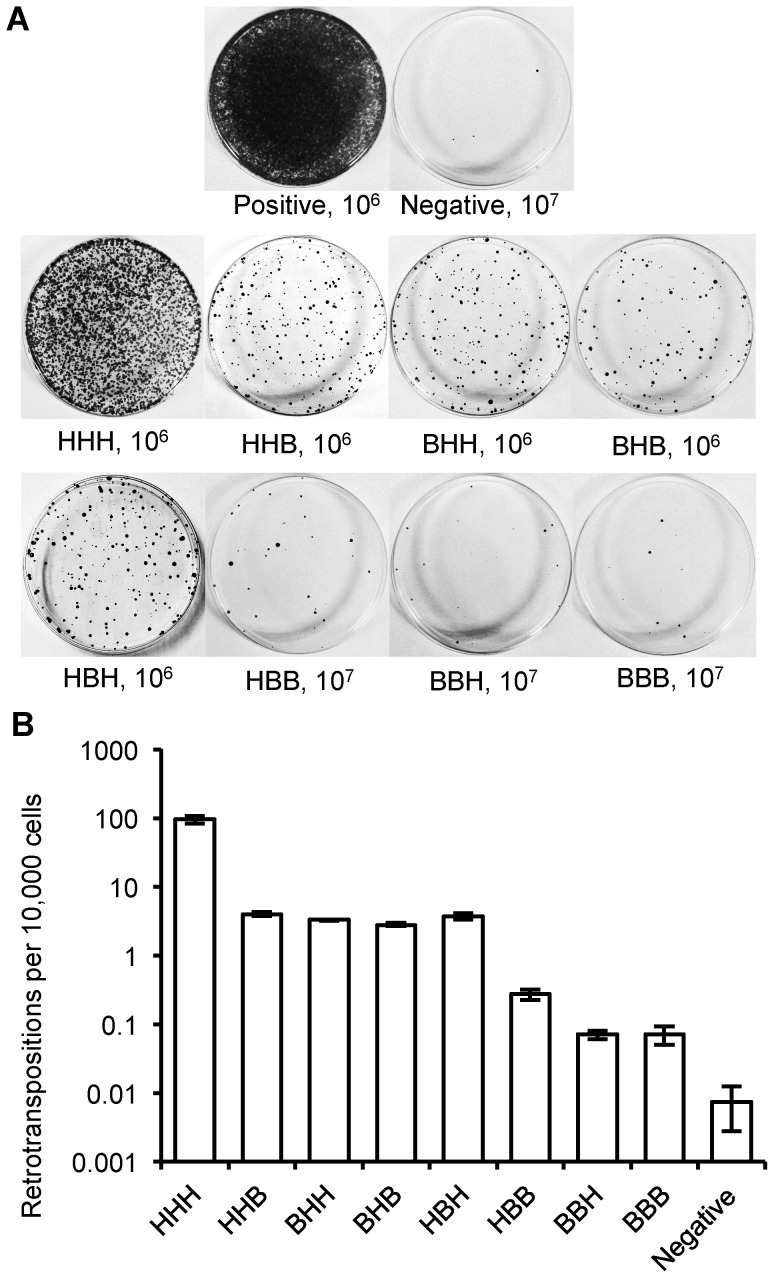
Retrotransposition rate of chimeric L1s. (A) Representative retrotransposition assay plates. Constructs are named with a three letter code based on the origin of their ORF1, IGR and ORF2: **H** for human L1rp; **B** for megabat lineage 1. An independent human L1 construct, pWA192 [67], was used as a positive control and an ORF1 mutant of L1rp [68] that blocks retrotransposition was used as a negative control. The number of cells seeded for G418 selection follows the name; 10-fold more cells were used for the negative control and for constructs with low retrotransposition rates. (B) Comparison of retrotransposition rates (log scale). At least 12 plates were counted for each construct in three independent replicate assays.

Both reconstructed megabat ORFs support retrotransposition, but at lower rates than the highly active human L1rp (Figure 4). Comparisons between the human L1 (HHH) and the constructs containing either one or both of the megabat ORFs (HHB, BHH and BHB) show that replacing the human ORFs with a corresponding megabat version reduces the retrotransposition rate ∼26-fold. We note that the heterologous nature of the chimeric construct could be responsible for part of the retrotransposition rate reduction as shown by Wagstaff *et al.* [63] with the human-mouse chimeras. We verified retrotransposition in two positive colonies from each construct by ascertaining splicing of the G418 resistance intron by PCR using primers flanking the *neo* cassette (Figure S2). An alternative start codon for ORF2, located in the IGR, would make ORF2 36 bp longer. We tested the retrotransposition rate of chimeric L1s based on this alternative ORF2 and no change in retrotransposition rate pattern was observed (data not shown).

**Figure 5:**
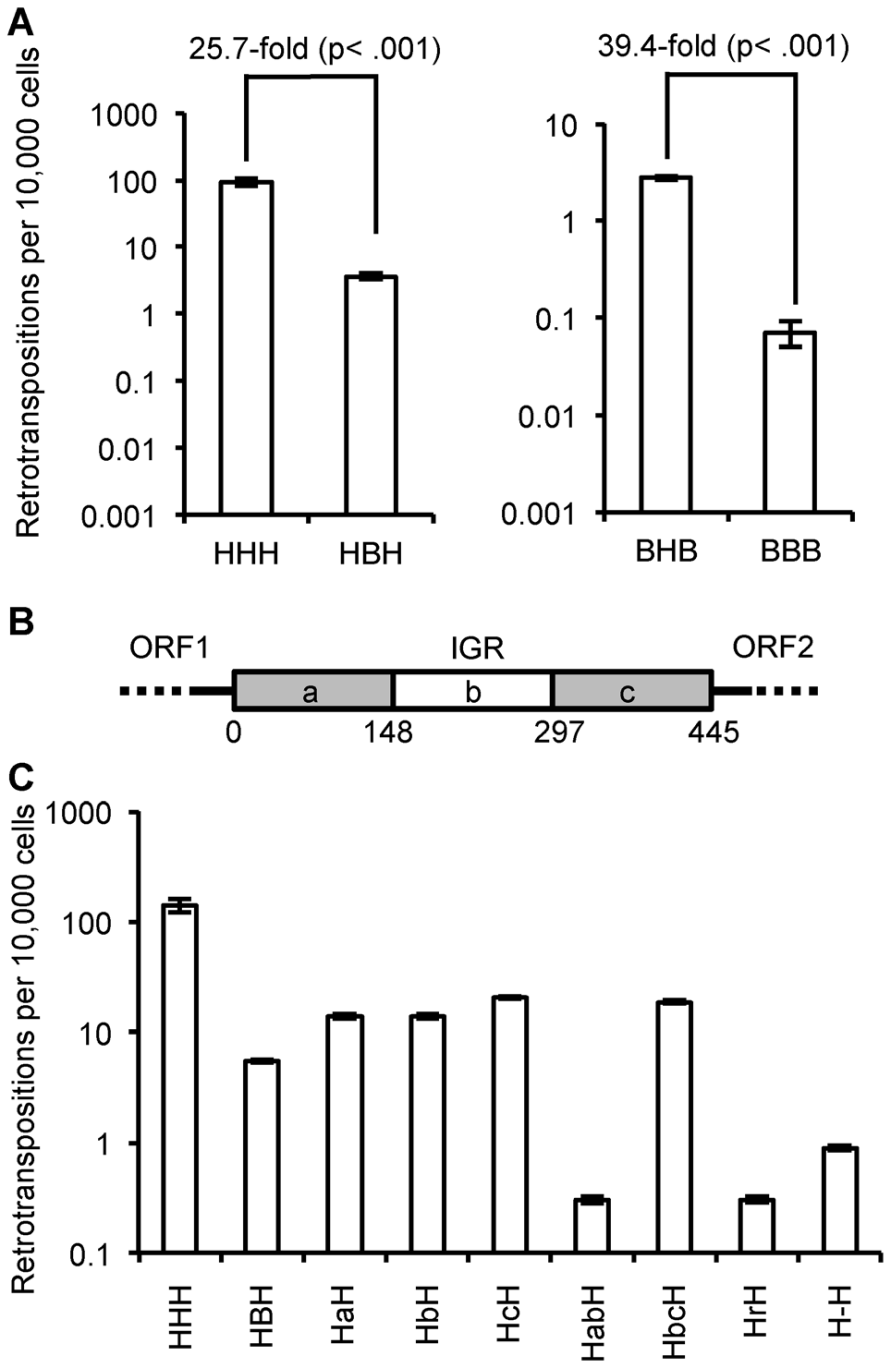
Effect of IGR on retrotransposition rate. (A) Heterologous IGRs: replacing the human L1 IGR with a megabat version reduces the retrotransposition rate ∼25.7-fold, while replacing the megabat IGR with a human L1rp IGR increases the retrotransposition ∼39.4-fold. (B) Schematic presentation of the manipulation of the reconstructed megabat L1 IGR: the IGR was truncated in one-thirds represented by ‘a’, ‘b’ and ‘c’, respectively. Numbers below the scheme indicate the coordinates of the split points of the truncations on the IGR. (C) Manipulated IGRs were tested in all chimeric L1 backgrounds and the results were qualitatively similar. Data are shown for replacement of the human L1rp IGR (HXH); data for the remaining L1 backgrounds are shown in Figure S3. At least four plates were counted per construct. Constructs are named by their composition of the ORFs and IGR. The first character represents the source of ORF1 and the last character represents the source of ORF2: ‘H’ indicates human and ‘B’ indicates megabat. The middle characters represent the manipulation of the IGR: ‘a’, ‘b’ and ‘c’ indicate the truncated IGR parts the construct contains as illustrated in (B) in the order they are present in the construct. For example, ‘HabH’ indicates a construct with human L1rp ORFs and the first two thirds of the truncated megabat L1 IGR. Other IGR manipulations are also abbreviated: ‘r’ indicates a shuffled version of the megabat IGR of the same length and nucleotide composition, and ‘-’ indicates the megabat IGR with all the AUG start codons (excluding the start at the beginning of ORF2) mutated to AGU.

The megabat IGR is inhibitory to retrotransposition. Replacing the native human L1 IGR with that of the reconstructed megabat (HHH→HBH) reduces the retrotransposition rate ∼26-fold, while introducing the human L1 IGR into the reconstructed megabat L1 (BBB→BHB) increases the retrotransposition rate ∼40-fold (Figure 5A). In a mixed ORF context (Figure 4B), both HHB→HBB and BHH→BBH result in ∼30-fold lower retrotransposition rates. Interestingly, the effect of the megabat IGR on the human construct (HHH→HBH) is similar to that seen when replacing either or both ORFs in the human construct with megabat ORFs (HHH→HHB, BHH or BHB). The retrotransposition rates of the chimeric L1s are drastically lowered with the combination of the reconstructed megabat IGR and any of the reconstructed megabat ORFs (BBH, HBB and BBB). Therefore, we conclude that compared to the HHH construct, the dampening effect of exchanging the ORFs is non-additive (BHB vs. HHB and BHH), while exchanging either ORF and the IGR at the same time is approximately additive (HHB vs. HBB, BHH vs. BBH and BHB vs. BBB). The hypothesis that retrotransposition rate is dependent on the amount of megabat L1 sequence in the construct is contradicted by the retrotransposition rate of BHB, which is largely made of megabat sequence but has a retrotransposition rate similar to those of constructs with only one bat segment (HHB, BHH and HBH).

### Dissecting the inhibitory property of the IGR

To further investigate the inhibitory effect of the reconstructed megabat IGR on retrotransposition and its interaction with the L1 ORFs, we manipulated the megabat IGR and tested variants in the chimeric L1 context. Manipulation of the IGR included truncated versions of the full-length IGR, a shuffled version with the same nucleotide composition (GenBank accession number KF796624) and an IGR with the sense-oriented AUG codons in all three reading frames mutated to AGU. We tested these variant IGRs in all four ORF contexts (HXH, HXB, BXH and BXB, where X indicates the IGR variant). We found that while the absolute level of transposition was affected by whether human or megabats ORFs were framing the IGR, the relative decrease in retrotransposition was comparable in all ORF contexts. Therefore, the effect of the manipulated IGR on retrotransposition is shown only in the human L1rp context, HXH, in Figure 5C; the retrotransposition rates of the manipulated IGRs in all other ORF contexts are shown in Figure S3.

To determine whether the inhibitory property of the megabat IGR is due solely to its length, we truncated one-third or two-thirds of the IGR from either the 5′ end, the 3′ end or both (Figure 5B). All the truncated IGRs increase the retrotransposition rate 0.3- to 0.5-fold compared to the full-length version (Figure 5C; HBH compared to HaH, HbH, HcH and HbcH) except the truncation of the 3′ one-third of the IGR (Figure 5C; HBH compared to HabH), which decreases the retrotransposition rate ∼6.9-fold. Thus, while the length of the IGR accounts for part of its retrotransposition inhibition property, there are also effects from other factors.

Although the megabat L1 IGR is inhibitory to retrotransposition compared to its human counterpart, we would expect to see that at this length, the reconstructed IGR still supports retrotransposition better than a randomized version with the same nucleotide composition. The randomized IGR with the same nucleotide composition reduces the retrotransposition rate ∼8.8-fold (Figure 5C; HBH compared to HrH), suggesting that there is co-adaptation of the resident IGR with the L1 ORFs.

Since it has been proposed that the translation of ORF2 is dependent on the existence of a close upstream ORF termination [4], we expected to see lowered retrotransposition rates with all the small ORFs within the IGR eliminated, as this makes the stop codon of ORF1 the closest stop upstream of ORF2 and reduces the probability that ORF2 translation will reinitiate before the ribosome is released from the L1 transcript. Mutating the AUG codons in all three possible frames of the IGR into AGUs decreases the retrotransposition rate ∼3.3-fold compared to the intact bat IGR (Figure 5C; HBH compared to H-H).

## Discussion

### Retrotransposition history of megabat L1s

The acknowledged pattern of L1 evolution is that the active elements within a genome are closely related, giving rise to a single active lineage which dominates the total retrotransposition in the genome for a period of time [38]. Eventually the active elements accumulate debilitating mutations and become less active, but occasionally a new active element derived from an old one will emerge in the L1 population. This element can behave like a ‘stealth driver’ [69] and remain at low activity in the genome for a long period of time. When evolution drives a new element to high activity, the elements derived from it can eventually dominate the genome and give rise to a new family. Repetition of this lifecycle of L1 families results in the periodic fluctuation of L1 activity.

Prior to L1 extinction, megabat L1s experienced periodic fluctuations in the number of elements fixed in the genome. This pattern is also observed in other mammalian clades, and in most cases each peak in copy number is dominated by a single L1 lineage. However, there are exceptions. For example, the human genome has been dominated by a single L1 lineage, but there was a period in primate evolution beginning about 46 MYA when two lineages were simultaneously active [35]. Similarly, two closely related lineages are currently active in the rodent genus *Peromyscus* [39]. Megabats stand out not only for the extinction of their L1s, but because their genomes have been continuously dominated by multiple active lineages with activity peaks of about the same age. Each peak includes two or three divergent families (Figure 2), a pattern that preceded the mammalian radiation and persisted throughout the history of L1 activity in megabats (Figure 1).

Where multiple lineages are maintained, it is possible that they are specialized on different tissue types (*e.g.*, on the male germ line vs. female germ line, or on the germ line vs. the embryo prior to differentiation of the primordial germ cells). Either of these scenarios could be successful in the evolutionary sense as mechanisms to avoid competition while still resulting in insertions that can be inherited by the next host generation. It is also possible that the L1 regulation mechanisms of the host are specific towards a certain lineage. Under that scenario, one lineage could dominate while the other is relatively quiescent, and eventually the second lineage could escape control and the first lineage be silenced. In other words, there would be no reason to expect that lineages would have the same peaks of increased retrotransposition. The fact that distinct lineages experienced fairly synchronized periods of activity and quiescence could suggest global rather than lineage-specific regulation of L1 retrotransposition. Peaks of L1 copy number are generally assumed to indicate transpositional bursts attributable to L1 activity, but other factors might account for peaks of L1 fixation in the genome. For example, host population bottlenecks could account for an increase in the rate of L1 fixation in the genome if there is selection against L1 [70], and such bottlenecks would be expected to affect multiple lineages in a similar manner, accounting for simultaneous peaks of fixation. Another possibility is that these peaks are related to the propensity of L1s to insert into double-stranded breaks [47], [51], [71], [72]. If a genome undergoes a period of extensive DNA damage due to an environmental or biotic assault, insertion into the resulting double-stranded breaks might lead to simultaneous peaks of retrotransposition of whatever L1 families are active at that time.

### Reconstruction of the last active L1 in megabats

To further characterize L1s in megabats at the time of their extinction, we reconstructed the full-length common ancestor of the most active family using a consensus-based method. Because of the unusual mode of L1 evolution [33], [36]–[38], consensus-based reconstruction is the preferred method of ancestral state reconstruction [56], [73]. Reconstruction is particularly challenging for an extinct L1 family because of variation between old L1 insertions that have accumulated private mutations after elements inserted into the genome; this variation eventually dwarfs changes that occur as one family gives rise to the next, and thus to the phylogenetic signal relevant to evolution within active lineages. Since progeny of the most active elements within a family are over-represented in the genome, the resulting reconstructed sequence can best be thought of as representing the most active L1 master sequence at the time of L1 extinction.

The reconstructed L1 sequence of megabat family 1A bears some of the features of a canonical L1 consensus from representative species, but also has some special characteristics to take into consideration. Although we identified and confirmed two amino acid changes in the reconstructed megabat ORF2 at sites that are conserved in all other species, such private changes at otherwise conserved sites were also frequently observed in the L1s used for comparison. The number of private changes in the L1s from a set of species varies from zero to seven with a median of two (Table S2 and Text S1and S2), which is in line with the number of private changes in the reconstructed megabat L1. These same two changes were observed in the RepBase reconstruction, providing further confidence that they are not artifacts. It should be noted that mutations in this set of mammalian L1s are not totally saturated, so conserved sites are not necessarily functionally constrained, but functionally constrained sites should be among the conserved sites. Some sites likely appear to be conserved because of the limited number of ORFs available for comparison.

An unusual aspect of L1 sequences is their high adenosine content on the coding strand and its possible dampening effect on transcription. This A-bias is prominent in the reconstructed megabat L1, which ranks the fifth among the 31 species surveyed. For comparison, the adenosine content of the megabat genome trace file (30%) is also slightly above the average level (29.5%) of the species surveyed (Table S2). The A-richness of L1 can cause elongation [62] and post-transcriptional splicing defects [74]. It may also give rise to a codon usage pattern in L1s that is different from the codon usage of host genes. This implies that the high A-content of the reconstructed L1 is a possible contributor to its own retrotransposition rate and likely to have a dampening effect. It has been shown that A-bias correction with codon optimization increases the retrotransposition rate of a native, ‘hot’ mouse L1 by ∼200-fold [62]. Although the same optimization only increases retrotransposition rate of human L1rp ∼3-fold, the transcription of the codon-optimized L1rp is increased >40-fold [67].

The most unexpected feature of the reconstructed megabat L1 is its long IGR. Alisch *et al.* [4] and Li *et al.* [5] have shown independently that the IGR is indispensable for the translation of L1 ORF2. The work of Alisch *et al.* [4] also demonstrated that the introduction of a long, structured IGR inhibits the retrotransposition of human L1s. This suggests that the long IGRs in megabat L1 lineage 1 may be inhibitory for retrotransposition. We cannot determine from examination of the megabat genome or from the work of Smit *et al.* [58] whether short or long spacers were ancestral among L1s of the Chiroptera (bats). However, L1s with long IGRs can be found in some marsupials, Laurasiatheria and Afrotheria species.

### Demonstration that the reconstructed sequences are active

To determine whether the reconstructed megabat lineage 1 element was active, we made chimeric sequences using human L1rp, a highly active *de novo* insertion, as a backbone [65], [66]. Ideally, these studies would have been carried out in both human and megabat cell lines. However, not all cell lines – and not all clones of permissive cell lines – support L1 retrotransposition. Megabat cell lines are not readily available, and we are unaware of an immortalized cell line from any bat that supports L1 activity. Fortunately, HeLa cells are competent hosts of heterologous and chimeric L1 retrotransposition. Mouse L1s readily retrotranspose in HeLa cells [75], [76] as do chimeras between human and mouse L1s [63]. However, our studies differ from those of Wagstaff *et al.* [63] in that we did not codon optimize our L1 constructs.

Although exchanging the L1rp ORFs with either or both of the corresponding megabat counterparts lowers the retrotransposition rate considerably, the activity of chimeric L1s is comparable to the majority of full-length human L1s. The retrotransposition rate of chimeric constructs containing megabat ORFs is much lower than the retrotransposition rate of the most active ‘hot’ L1s, but more active than 82% of full-length L1s in the human reference genome [41]. The retrotransposition rate of BBB is even lower, but still surpasses that of 56% of full-length L1s in the human reference genome.

There are some caveats relevant to this comparison. First, the retrotransposition assays of Brouha *et al.* [41] were conducted in a different genetic background from the one in this study, but both studies use relative numbers normalized by the retrotransposition rate of L1rp, and thus are comparable. Secondly, although the reconstructed megabat L1 (BBB) supported retrotransposition at about the rate of the average active human L1, it would not be expected to generate half the number of insertion events as a ‘hot’ human L1 because the contribution of individual active L1s to the total retrotransposition activity is unevenly distributed – just six ‘hot’ elements of the 80–100 full-length human L1s are responsible for more than 80% of the total retrotransposition activity [41]. Since the average human L1 barely contributes to the total L1 retrotransposition in the genome, we conclude that the intact reconstructed megabat L1 is able to retrotranspose, but by this measure transposes at a very low rate. The reconstruction did not include the promoter, as L1 retrotransposition driven by a native promoter is difficult to detect in tissue culture assays [64]. Therefore, interactions with heterologous regulatory sequences are not a factor in this assay. No single component of the reconstructed L1s was responsible for the inhibition of retrotransposition compared to L1rp; replacement of each component had a similar effect. This makes it unlikely that either a rate-limiting megabat L1 protein or an interaction with a specific host factor is responsible for dampening activity. We also note that these assays were conducted in a human cell line (HeLa), which is heterologous to the reconstructed L1, so these estimates must be interpreted with caution.

### Conclusion

Demonstrating activity of a reconstructed element in a tissue culture assay is the ultimate test of the quality of the reconstruction. To our knowledge, this is the first L1 element from a species that does not carry currently active L1s to be resurrected and tested for activity. However, ancestral L1s have been extensively reconstructed [58] and some of these reconstructions and their codon-optimized variants have been tested for activity in tissue culture assays. For example, Wagstaff *et al.* [73] showed that reconstructed ancestral L1 from primates are capable of retrotransposition. Another good example of a reconstructed ancient transposable element is Sleeping Beauty [77], [78], an element from fish which is active in human cells and has proven to be a powerful tool for genetic engineering. These reconstructed elements are ancient snapshots from lineages that have been co-evolving with a suite of host factors. It is important to remember that while we can reconstruct the sequence of the ancestral element, we cannot replicate the exact genetic context under which these reconstructed elements were active.

RepBase have been actively reconstructing and hosting reconstructed ancestral transposable elements since its establishment [56], [57]. However, detailed studies of the evolutionary history of a particular transposable element family usually focus on model organisms. The evolutionary history of human [35] and mouse [34] L1 lineages have been well-documented, but data are sparse for most mammalian clades. The work reported here complements that of Khan *et al.* [35] and Sookdeo *et al.* [34], demonstrating the diversity of mammalian L1 evolution patterns and allowing us to understand mammalian L1 evolution at a broader level.

The most striking feature of the reconstructed megabat L1 is the long IGR, which is is co-adapted with the ORFs to support retrotransposition. This is most evident in the comparison of the randomized IGR with the intact version (Figure 5C), where retrotransposition with the intact IGR is 8.8-fold higher than the randomized version with the same base composition. Although the length of the IGR has a major effect on retrotransposition rate, other factors such as secondary structure and splicing sites of the L1 transcript can also dramatically change the retrotransposition rate. Li *et al.* [5] demonstrated that the IGR of a ‘hot’ mouse L1, L1spa, contains an IRES that enhances the translation of a downstream ORF, and the work of Alisch *et al.* [4] suggests that the termination of another ORF directly upstream of the ORF2 start is the key for its translation. Our data demonstrate that the reconstructed L1 containing an AUG-codon-free IGR has a lower retrotransposition rate than that of the intact version. This is in line with the evidence found by Alisch *et al.* [4] as well as the original work by Horvath *et al.* [79] that proposes a reinitiation mechanism for the translation of dicistronic structures.

Perhaps the most difficult aspect to reconcile about the long IGR in lineage 1 is its evolutionary persistence. An active element that deleted this long IGR would be expected to dramatically increase its retrotransposition rate and, thus, to dominate future retrotransposition. That is to say, there should have been strong selection favoring the deletion of the IGR. One might expect such a deletion to be ‘easy’ from an evolutionary perspective since it need not maintain a reading frame, and yet this did not happen.

The tempo of L1 retrotransposition in megabats directly preceding L1 extinction is also noteworthy. A significant burst of retrotransposition occurred just prior to L1 extinction in megabats, contributing 25% of the detectable L1s to the genome. Family 1A accounts for the bulk of this activity – 18% of the total detectable elements in the genome – despite the demonstrated inhibitory effect of the long intergenic spacer on this family. The IGR has a long evolutionary history in this L1 lineage and likely preceded the evolution of megabats. Thus, despite its inhibitory effect on retrotransposition, it is unlikely that it contributed to L1 extinction.

There are some characteristics of bat genomes that make them unique among the mammals. Bats, and especially megabats, have much smaller genomes than other mammals [80]. Data from 43 species of megabats, 62 species of microbats and ∼10,000 other mammalian species suggest that at 2.15 Gbp the megabat average genome size is significantly more constrained than the average of all mammals (3.42 Gbp) and is considerably smaller than even the microbats (2.52 Gbp). It has been proposed that small genome size is related to the ability to fly given the high metabolic rate and small cell size requirements of flight [81]–[83]. For example, it has been shown that bird genomes are smaller and less variable in size than genomes of mammals and amphibians [80] and that their genome size is inversely correlated with their wing loading, an index of flight ability [84].

Since transposable elements are the major contributor to mammalian genome size [85], pressure to constrain genome size will likely be reflected by stronger regulation of transposable elements. This regulation could theoretically result in both suppression of transposition and more efficient removal of inserted elements from the genome. Loss of L1 activity would be particularly effective in slowing expansion of the genome since L1s and the SINEs (Short INterspersed Elements), that co-op the L1 replication machinery, together make up approximately a quarter of a typical mammalian genome [40], [42]. Compared to other mammals, genome size constraint in bats confers a stronger selective pressure on the host defense mechanisms that control L1 retrotransposition, which could serve as the intrinsic driver for the host to develop anti-transposable element strategies that may increase the likelihood of transposable element quiescence and extinction in this group.

## Materials and Methods

### Bioinformatic analysis of L1 history in megabats

Since the large majority of L1s are truncated at the 5′ end [86], the copy number of 3′ ends better represents the history of retrotransposition events. Therefore, we used 575 bp in the 3′ end of L1 ORF2 (as reconstructed below) to get a comprehensive view of L1 retrotransposition. Using the megabat L1 lineage 1 [50] consensus as the query sequence, we ran CENSOR 4.2 [87] against the ∼2× genome trace files of *P. vampyrus* (Baylor College of Medicine, ftp.ncbi.nlm.nih.gov/pub/TraceDB/pteropus_vampyrus/) to find detectable sequences with >60% identity and >90% coverage of the query. Using 2000 random sequences from the CENSOR run, subfamilies were identified based on shared sequence variants (co-segregating mutations) with COSEG 0.2.1 (http://www.repeatmasker.org/COSEGDownload.html) [55] following the default parameters. Nine subfamilies were generated and their consensuses used as query sequences for a second round of CENSOR against the *P. vampyrus* genome. All identified L1 sequences from the second CENSOR run were used for a second round of COSEG, which required the additional parameter of at least 250 sequences to form a subfamily. Consensuses of the 64 subfamilies thus generated were used as query sequences to run CENSOR for a third time. Each hit's percent identity to the corresponding query was used to assign it to a L1 subfamily, and the copy numbers in each subfamily were counted. Seven subfamilies containing less than 250 sequences were removed. Consensuses from each of the remaining 57 subfamilies were used as query sequences to run CENSOR for a fourth time and all detected L1s were assigned to their subfamilies by the percent identity of each hit to its query. The 57 subfamily consensuses were aligned with ancestral mammalian L1s from RepBase [56], [57], reconstructed by Smit *et al.* [58] and Wade *et al.* [59], with the Lasergene software suite (DNASTAR, Madison, WI), and a distance matrix was calculated. Based on the alignment, a maximum likelihood tree was constructed using PhyML [88] with the GTR+I+G model and 100 bootstrap replicates (Figure S1). L1s were then assigned to families based on a <3.5% within-family pairwise distance from their subfamily consensuses. Sequence specificity of L1 families was determined by BLAST [89] against the NCBI whole genome sequencing databases. The consensus sequences of subfamilies 1, 5, 7, 3, 40, 36, 34, 0 and 29 were used as the BLAST queries representing families 1A, 1B, 1C, 2A, 2B, 2C, 2D, 3 and 4, respectively. A subfamily and its corresponding family were considered bat-specific only if <5 of the top 100 BLAST hits were not from bats.

Histograms of L1 age distribution were generated by the R [90] histogram function using a window size of 0.5% (Figures 1 and 2). Percent identities corresponding to retrotransposition peaks of individual families (Figure 2) were determined by R using the kernel smoothing function with 0.2% bandwidth.

### Bioinformatic reconstruction of an extinct megabat L1

A full-length consensus sequence of the most recently active L1 from megabat lineage 1 was reconstructed by a series of progressive steps. The seed for the reconstruction was a conserved 575 bp region in the 3′ half of ORF2 (Figure 3A). This region was previously amplified by degenerate PCR and a consensus sequence was determined [91]. Walks were performed in the 5′ and 3′ directions away from the cloned region and continued in both directions until full-length L1s were reconstructed. To aid with the reconstruction, a software pipeline was developed consisting of Perl (http://www.perl.org/), Ruby (https://www.ruby-lang.org/en/) and Bash (http://www.gnu.org/software/bash/) scripts. The pipeline queried, filtered and extracted data from the genome of *P. vampyrus*. An individual step resulted in the addition of 100–500 bp of sequence to the consensus, depending on the quality of the alignment at the ends, which was then used in the next step of the walk and in the final L1 reconstruction. Candidate sequences were identified in the database using BLAST with default parameters and an e-value of 1×10^−50^, parsed through the BioPerl SearchIO module (http://www.bioperl.org) and screened based on their similarity to the input sequence. Only hits with at least 92% identity were retained to assure that the reconstruction did not include older lineages, and then a Ruby script extracted those sequences with overhangs of at least 100 bp. Alignments for each end were created and hand-edited to yield consensuses of clean read which were aligned into a master alignment. A 300–500 bp region from each end was selected to act as the seeds for the next step in the walk. The process was repeated until the entire element was reconstructed. Upon completion of the full-length L1, a 500 bp seed was chosen arbitrarily from the final consensus and the pipeline was run again to verify the reconstruction. Methylated CpG sites evolve rapidly and must be corrected in the final consensus. CpG sites were identified by their high variation and the presence of dinucleotide sequence CG, CA, TG or TA; these were examined, manually edited and designated as CG in the final consensus. This pipeline also reconstructed the most recently active L1 lineage of four additional species listed in Table S1, but required higher percent identities for the walks to reduce the noise introduced by older lineages.

To compare the reconstruction of the extinct L1 to other L1s, sequences from a range of mammalian species were either reconstructed as described above, or selected from the RepBase report of February 2013 [56]. L1 consensuses of all species available in RepBase were aligned except those of dolphin and American opossum which had problematic regions of non-homology. When multiple L1 consensus sequences for the same species were present in RepBase, the one with highest average percent identity to its genomic sequence was chosen to represent the most recent master L1 in the genome. Some of the RepBase L1 sequences were out of frame at regions containing adenosine runs or contained in-frame stop codons, both resulting in significantly shorter ORFs. The following corrections brought these sequences into the correct reading frame: L1-1_Cpo, ignored an in-frame stop codon at bp 3050–3052 and used the original sequence for the alignment; L1-1_DV, added a N after bp 6015; and, L1A_Mim, deleted an A at bp 1590–1591 and bp 5336–5337.

### Synthesis and cloning of the chimeric L1s

The backbone plasmid for chimera constructions used in the retrotransposition assays was based on pL1PA1tag, a gift from Dr. Astrid Roy-Engel. pL1PA1tag contains a codon-optimized consensus of the PA1 family of human L1 in a pBSSK^−^ (Agilent Technologies, Inc., Santa Clara, CA) backbone. A puromycin resistance gene and its affiliated promoter pPGKpuro (Addgene, Cambridge, MA) were cloned into pL1PA1tag, creating plasmid pLY1004. The L1 insert of pLY1004 was removed by *Nhe*I and *Eco*RI digestion, creating the final plasmid backbone (Figures 3B and 3C).

The reconstructed L1 and manipulated IGR sequences were commercially synthesized by GenScript USA, Inc. (Piscataway, NJ). Reconstructed L1s were synthesized in two blocks consisting of ORF1+IGR and ORF2. The manipulated IGRs were synthesized separately or in combinations containing distinct cloning sites. The synthesized sequences were cloned into pUC57 with flanking ends compatible to the linearized pLY1004 backbone and with *Bsa*I or *Bsm*BI sites to generate compatible overhangs after digestion. ORF1 and IGR were subcloned into separate pUC57 plasmids. Figure 3B illustrates the principle underlying the construction of the chimeric L1s. L1 ORFs and IGRs were amplified from these plasmids by PCR with Phusion high-fidelity polymerase (ThermoFisher Scientific, Waltham, MA) using primers designed to generate compatible overhangs when the PCR products are digested with *Bsa*I, *Btg*ZI or *Eco*RI. Human L1rp segments were cloned from pWA192 [67], a gift from Dr. Wenfeng An, using the same principle. The L1 ORFs, IGRs and the linearized backbone plasmid pLY1004 were joined together by a multi-way ligation using T4 DNA ligase. All restriction enzymes and DNA modifying enzymes were from New England BioLabs, Inc. (Ipswich, MA) unless otherwise specified. All constructs were confirmed by sequencing the L1 insert.

### Retrotransposition assays

Retrotransposition rates were tested in an assay derived from Moran *et al.* [61], in which the number of cell colonies surviving G418 antibiotic selection represents the retrotransposition rate (Figure 3C). Briefly, the transcription and retrotransposition of L1 trigger the splicing of the transcript and excision of the intron of the inverse-oriented *neo* cassette, granting the cell resistance to the antibiotic G418.

The HeLa cell line (ATCC CCL-2) was a gift from Dr. Wenfeng An and maintained in Dulbecco's Modified Eagle Medium with 4500 mg/L glucose and 110 mg/L sodium pyruvate (ThermoFisher Scientific) supplemented by 10% fetal bovine serum (Atlanta Biologicals, Lawrenceville, GA), 2 mM l-alanyl-l-glutamine dipeptide and 100 units/mL Penicillin-Streptomycin (ThermoFisher Scientific). The assay was conducted as described by An *et al.* [67]. The culture medium for antibiotic selection was similar to the cell maintenance medium except 2.5 ug/mL puromycin (CALBIOCHEM, Billerica, MA) or 50 mg/mL G418 (CALBIOCHEM) was added. Plasmids for transfection were prepared with the Promega (Fitchburg, WI) PureYield Plasmid Midiprep System and the cells were transfected with FuGENE HD transfection reagent (Promega) following the manufacturer's protocol. Retrotransposition assays of the chimeric L1s were repeated at least 12 times in three different batches and manipulated IGR assays were repeated at least four times.

To confirm retrotransposition, two retrotransposition-positive colonies of each chimeric L1 construct were isolated with cloning rings, dissociated with trypsin (ThermoFisher Scientific), seeded on T75 flasks and allowed to grow into confluence. Cells were harvested and their genomic DNA was extracted with the QIAamp DNA mini kit (QIAGEN, Germantown, MD). Genotyping PCRs were conducted with primers bracketing the intron of the G418 reporter gene as described by An *et al.* [92]. Briefly, genotyping PCR primers were designed to the *neo* cassette so that cells hosting retrotransposition events, and the corresponding spliced cassettes, yield 653 bp PCR products. pLY1101, a self-ligated version of the linearized pLY1004 without a L1 insertion, was constructed as a positive control; genotyping PCR of pLY1101 yields a 1556 bp construct corresponding to the unspliced *neo* cassette.

## Supporting Information

Figure S1 Maximum likelihood tree of the detected megabat L1 subfamilies. Selected ancestral mammalian L1 families, labeled L1MXX, are included to facilitate comparison. The tree was constructed using PhyML [88] with the GTR+I+G model and 100 bootstrap replicates. Bootstrap values >80 are shown. L1 families are designated to the right of the corresponding subfamilies according to Materials and Methods and Table S1.
(TIF)

Figure S2 Confirmation of retrotransposition. Retrotransposition was confirmed for each construct by PCR of the *neo* cassette from two surviving colonies. Genomic DNA was extracted and used as template. Genotyping PCR primers were designed to amplify the *neo* cassette so that cells hosting retrotransposition events, and thus the spliced cassette, yield 653 bp PCR products. PCR of positive control construct pLY1101, identical to backbone pLY1004 but with no L1 insertion, yields a 1556 bp product that corresponds to the unspliced *neo* cassette. The 653 bp band was detected from all colonies. Non-specific bands were detected in a few cases; these were not further characterized.
(TIF)

Figure S3 Effect of IGR on retrotransposition rate. Results are shown for all chimeric backgrounds on representative retrotransposition assay plates. Columns represent the various genetic contexts of ORF1/IGR/ORF2; H indicates human L1rp sequence, B indicates reconstructed megabat L1 and X corresponds to the IGR manipulation assayed in each row. Characters to the left of the rows indicate the truncation of the megabat IGR as represented in Figure 5B: ‘a’, ‘b’ and ‘c’ indicates the truncated IGR parts the construct contains as illustrated in the order they are present in the construct. For example, ‘HabH’ indicates a construct with human L1rp ORFs and the first two thirds of the truncated megabat L1 IGR. ‘r’ indicates a shuffled version of the megabat IGR with the same length and nucleotide composition, and ‘-’ indicates the megabat IGR with all the AUG start codons (excluding the start at the beginning of ORF2) mutated to AGU.
(TIF)

Table S1 Summary of megabat L1 families. Families are based on <3.5% distance among the corresponding subfamilies identified by COSEG and shown in Figure S1. ‘Ancestral L1s’ are the ancestral mammalian L1 families found in RepBase most closely related to the corresponding megabat families. ‘Fraction’ indicates the percent of 79,978 total detected megabat L1s in that family. ‘Mean identity’ refers to the average percent identity of the sequences in each family to their corresponding subfamily consensus, and ‘peak identity’ refers to the peak of the distribution of the same dataset determined by kernel smoothing as described in Materials and Methods.
(DOCX)

Table S2 Sequences from RepBase are indicated with an X; other sequences were constructed from genomic trace files. Adenosine content is compared between genomic DNA and L1 segments. AT content from the NCBI genome database was divided by two for Genomic %A and does not take into account any strand bias in coding regions. L1 %As were determined from the coding strands. Numbers in parentheses in the IGR length column indicate IGR lengths from alternative ORF2 starts. Average %As and IGR length are in the bottom row.
(DOCX)

Text S1 Alignment of L1 ORF1 sequences. Protein alignment of the homologous region of ORF1, amino acids 123–321, bp 1273–1869 of L1rp (GenBank accession number AF148856), including the reconstructed megabat L1 lineage 1 (L1-2_PVa), megabat L1 lineage 2 (L1-1_PVa), 26 RepBase-reconstructed L1 consensuses and four L1s reconstructed by us as described in Materials and Methods. ‘Conserved sites’ are the conserved amino acid sites among the surveyed species excluding the megabat L1s. L1rp is not shown in the alignment but shares the same nucleotide and amino acid coordinates with L1HS.
(PDF)

Text S2 Alignment of L1 ORF2 sequences. Protein alignment of the homologous region of ORF2 spanning the full length L1rp ORF2 (bp 1987–5814, GenBank accession number AF148856), including the reconstructed megabat L1 lineage 1 (L1-2_PVa), megabat L1 lineage 2 (L1-1_PVa), 26 RepBase-reconstructed L1 consensuses and four L1s reconstructed by us as described in Materials and Methods. ‘Conserved sites’ are the conserved amino acid sites among the surveyed species excluding the megabat L1s. L1rp is not shown in the alignment but shares the same nucleotide and amino acid coordinates with L1HS.
(PDF)
