# Supplementary figures and images for: Reviving the Dead: History and Reactivation of an Extinct L1
Source: PLoS Genet. 2014 Jun 26;10(6):e1004395. doi: 10.1371/journal.pgen.1004395 (PMC4072516; doi:10.1371/journal.pgen.1004395)

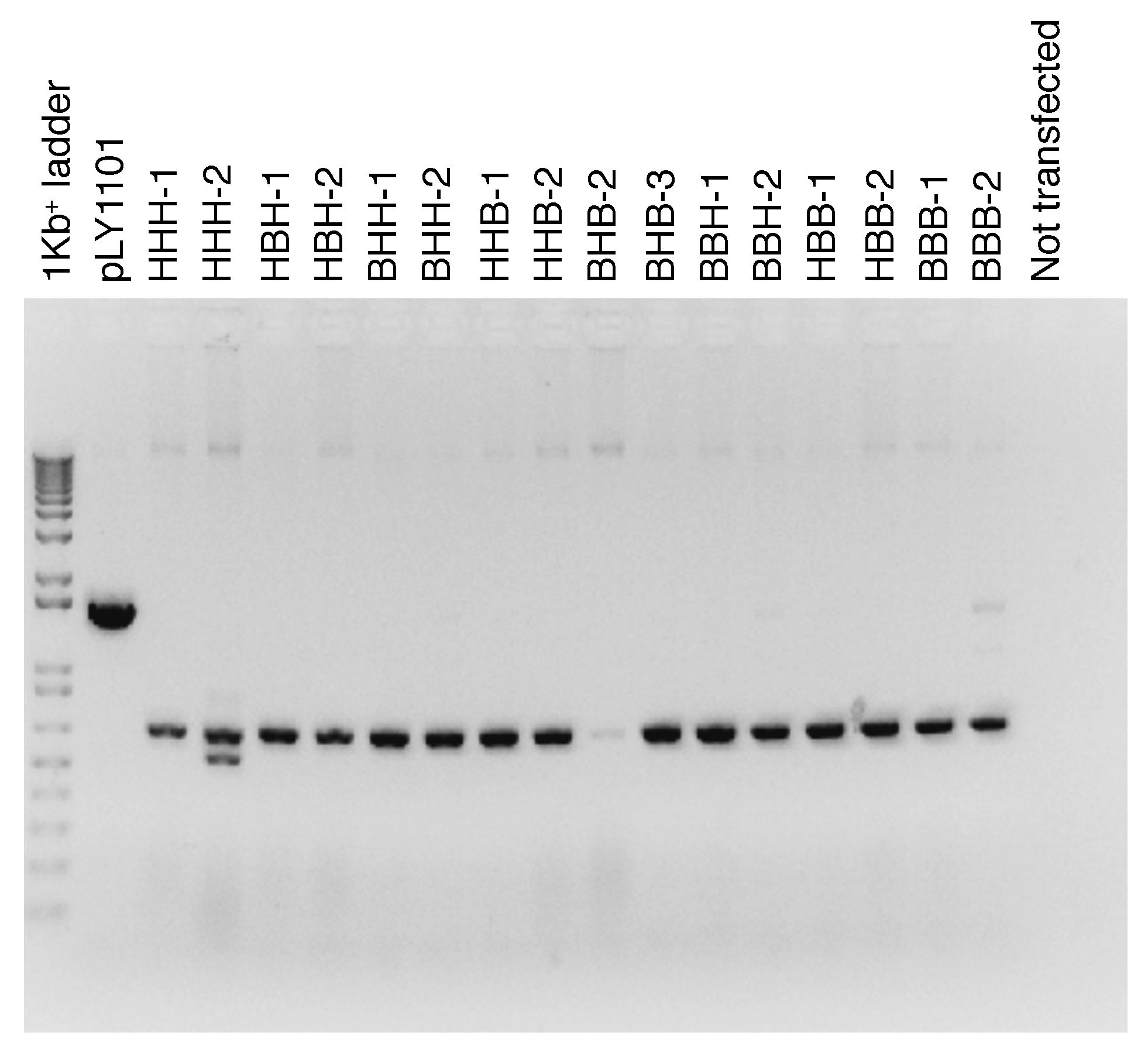

Supplement: Figure S2 — Confirmation of retrotransposition. Retrotransposition was confirmed for each construct by PCR of the neo cassette from two surviving colonies. Genomic DNA was extracted and used as template. Genotyping PCR primers were designed to amplify the neo cassette so that cells hosting retrotransposition events, and thus the spliced cassette, yield 653 bp PCR products. PCR of positive control construct pLY1101, identical to backbone pLY1004 but with no L1 insertion, yields a 1556 bp product that corresponds to the unspliced neo cassette. The 653 bp band was detected from all colonies. Non-specific bands were detected in a few cases; these were not further characterized. (TIF) [file pgen.1004395.s002.tif]

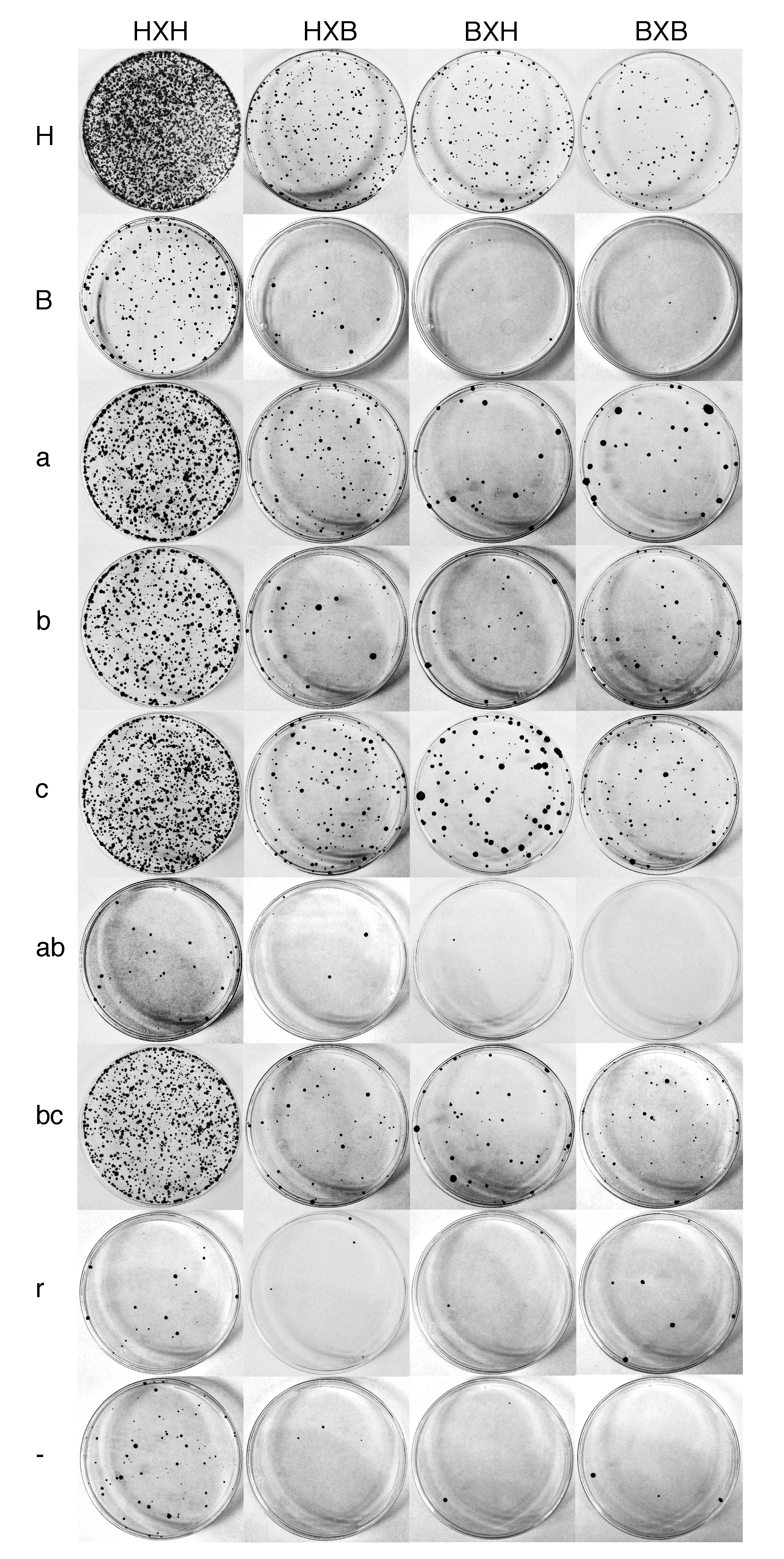

Supplement: Figure S3 — Effect of IGR on retrotransposition rate. Results are shown for all chimeric backgrounds on representative retrotransposition assay plates. Columns represent the various genetic contexts of ORF1/IGR/ORF2; H indicates human L1rp sequence, B indicates reconstructed megabat L1 and X corresponds to the IGR manipulation assayed in each row. Characters to the left of the rows indicate the truncation of the megabat IGR as represented in Figure 5B: ‘a’, ‘b’ and ‘c’ indicates the truncated IGR parts the construct contains as illustrated in the order they are present in the construct. For example, ‘HabH’ indicates a construct with human L1rp ORFs and the first two thirds of the truncated megabat L1 IGR. ‘r’ indicates a shuffled version of the megabat IGR with the same length and nucleotide composition, and ‘-’ indicates the megabat IGR with all the AUG start codons (excluding the start at the beginning of ORF2) mutated to AGU. (TIF) [file pgen.1004395.s003.tif]
