# Supplementary material for: Reviving the Dead: History and Reactivation of an Extinct L1
Source: PLoS Genet. 2014 Jun 26;10(6):e1004395. doi: 10.1371/journal.pgen.1004395 (PMC4072516; doi:10.1371/journal.pgen.1004395)
Supplement: Table S1 — Summary of megabat L1 families. Families are based on <3.5% distance among the corresponding subfamilies identified by COSEG and shown in Figure S1. ‘Ancestral L1s’ are the ancestral mammalian L1 families found in RepBase most closely related to the corresponding megabat families. ‘Fraction’ indicates the percent of 79,978 total detected megabat L1s in that family. ‘Mean identity’ refers to the average percent identity of the sequences in each family to their corresponding subfamily consensus, and ‘peak identity’ refers to the peak of the distribution of the same dataset determined by kernel smoothing as described in Materials and Methods. (DOCX) [file pgen.1004395.s004.docx]

Table S1. Summary of the megabat L1 families.

| Family | Subfamilies | Ancestral L1 | Fraction (%) | Mean identity (%) | Peak identity (%) |
| --- | --- | --- | --- | --- | --- |
| 1A | 1, 52, 55-58, 60-63 | - | 18 | 92 | 93 |
| 1B | 5, 48-51 | - | 5 | 88 | 89 |
| 1C | 7, 47 | - | 1 | 86 | 87 |
| 2A | 3, 44-46 | - | 7 | 91 | 92 |
| 2B | 39, 40 | - | 3 | 87 | 88 |
| 2C | 15, 36-38, 59 | - | 5 | 87 | 88 |
| 2D | 10, 33-35 | - | 3 | 85 | 85 |
| 3 | 0, 29, 30, 32 | **-** | 5 | 84 | 85 |
| 4 | 11, 26-28 | L1MAB_EC | 4 | 83 | 83 |
| 5 | 6, 25 | L1MB1 | 2 | 81 | 82 |
| 6 | 13, 23, 24 | L1MB3 | 5 | 82 | 83 |
| 7 | 14, 21 | L1MB4 | 3 | 81 | 82 |
| 8 | 2, 16, 19, 20, 22 | L1MB7 | 12 | 80 | 82 |
| 9 | 4, 54 | L1MC1 | 7 | 82 | 84 |
| 10 | 12 | L1ME | 6 | 76 | 77 |
| 11 | 17, 18 | L1ME | 12 | 74 | 76 |
