# Supplementary material for: Reviving the Dead: History and Reactivation of an Extinct L1
Source: PLoS Genet. 2014 Jun 26;10(6):e1004395. doi: 10.1371/journal.pgen.1004395 (PMC4072516; doi:10.1371/journal.pgen.1004395)
Supplement: Table S2 — Sequences from RepBase are indicated with an X; other sequences were constructed from genomic trace files. Adenosine content is compared between genomic DNA and L1 segments. AT content from the NCBI genome database was divided by two for Genomic %A and does not take into account any strand bias in coding regions. L1 %As were determined from the coding strands. Numbers in parentheses in the IGR length column indicate IGR lengths from alternative ORF2 starts. Average %As and IGR length are in the bottom row. (DOCX) [file pgen.1004395.s005.docx]

| name / abbreviation | RepBase | latin name | common name | genomic %A | L1 %A ORFs | L1 %A IGR | L1 %A ORFs+IGR | IGR length |
| --- | --- | --- | --- | --- | --- | --- | --- | --- |
| family 2A (L1-1_PVa) | x | *Pteropus vampyrus* | large flying fox | 30.0 | 44.1 | 65.8 | 44.3 | 38 |
| family 1A (L1-2_PVa) | x | *Pteropus vampyrus* | large flying fox | 30.0 | 43.4 | 47.2 | 43.7 | 407(445) |
| L1-Y_CF | x | *Canis lupus familiaris* | dog | 29.1 | 39.4 | 46.9 | 39.5 | 49 |
| L1MAB2_ML | x | *Myotis lucifugus* | little brown bat | 28.8 | 42.6 | 60.0 | 42.9 | 55 |
| L1HS | x | *Homo sapiens* | human | 29.2 | 40.7 | 44.4 | 40.7 | 63 |
| L1-BT | x | *Bos taurus* | cow | 29.1 | 44.4 | 53.8 | 44.5 | 52 |
| L1A_OC | x | *Oryctolagus cuniculus* | rabbit | 28.0 | 40.9 | 44.4 | 41.0 | 81 |
| L1A_Mim | x | *Microcebus murinus* | mouse lemur | 29.3 | 41.4 | 53.7 | 41.5 | 41(86) |
| L1-2_EC | x | *Equus caballus* | horse | 29.2 | 42.9 | 47.6 | 43.2 | 328 |
| L1-2_Dor | x | *Dipodomys ordii* | kangaroo rat | 28.8 | 40.5 | 32.5 | 40.4 | 40 |
| L1-1B_Cho | x | *Choloepus hoffmanni* | two-toed sloth | 30.5 | 43.1 | 48.8 | 43.2 | 82 |
| L1-1A2_Sar | x | *Sorex araneus* | common shrew | 28.6 | 39.2 | 21.5 | 39.3 | 43 |
| L1-1_Vpa | x | *Vicugna pacos* | alpaca | 29.3 | 43.7 | 46.2 | 43.9 | 442 |
| L1-1_TS | x | *Tarsius syrichta* | Philippine tarsier | 30.1 | 42.1 | 61.1 | 42.4 | 90 |
| L1-1_Tbel | x | *Tupaia belangeri* | tree shrew | 29.3 | 41.0 | 60.5 | 41.1 | 43(55) |
| L1-1_Str | x | *Ictidomys tridecemlineatus* | 13-lined ground squirrel | 30.1 | 42.7 | 51.2 | 42.8 | 82 |
| L1-1_SSc | x | *Sus scrofa* | pig | 30.9 | 43.0 | 61.8 | 43.2 | 68 |
| L1-1_Pca | x | *Procavia capensis* | rock hyrax | 29.5 | 41.0 | 41.8 | 41.0 | 421 |
| L1-1_OP | x | *Ochotona princeps* | American pika | 28.4 | 41.7 | 47.2 | 41.7 | 53 |
| L1-1_MD | x | *Monodelphis domestica* | gray short-tailed opossum | 31.0 | 43.1 | 40.1 | 42.7 | 531 |
| L1-1_LA | x | *Loxodonta africana* | African elephant | 29.6 | 43.4 | 49.6 | 43.9 | 458 |
| L1-1_ET | x | *Echinops telfairi* | lesser hedgehog (tenrec) | 28.5 | 39.1 | 47.6 | 39.1 | 42 |
| L1-1_EE | x | *Erinaceus europaeus* | European hedgehog | 29.3 | 41.0 | 50.0 | 41.1 | 56 |
| L1-1_DN | x | *Dasypus novemcinctus* | armadillo | 29.6 | 43.1 | 66.7 | 43.5 | 75 |
| L1-1_Cpo | x | *Cavia porcellus* | guinea pig | 30.1 | 43.2 | 66.7 | 43.3 | 18(39) |
| L1-1_Cja | x | *Callithrix jacchus* | marmoset | 29.4 | 41.2 | 46.0 | 41.3 | 63 |
| L1-1_AMe | x | *Ailuropoda melanoleuca* | giant panda | 29.2 | 39.2 | 37.8 | 39.0 | 580 |
| L1_RN | x | *Rattus norvegicus* | brown rat | 28.7 | 41.4 | 47.5 | 41.5 | 59 |
| Fcat |  | *Felis cattus* | domestic cat | 27.4 | 40.7 | 48.0 | 40.6 | 50 |
| Pham |  | *Papio hamadryas* | hamadryas baboon | NA | 40.8 | 44.4 | 40.7 | 63 |
| Mmul |  | *Macaca mulatta* | rhesus monkey | 29.3 | 40.8 | 44.4 | 40.8 | 63 |
| Meug |  | *Macropus eugenii* | tammar wallaby | 32.7 | 41.6 | 39.3 | 41.9 | 565 |
| Opal |  | *Oryzomys palustris* | marsh rice rat | NA | 42.7 | 52.2 | 42.7 | 23 |
| Average |  |  |  | 29.4 | 41.8 | 49.0 | 41.9 | 155(159) |

Table S2. L1 consensus sequences used for comparison.
