# Supplementary material for: Reviving the Dead: History and Reactivation of an Extinct L1
Source: PLoS Genet. 2014 Jun 26;10(6):e1004395. doi: 10.1371/journal.pgen.1004395 (PMC4072516; doi:10.1371/journal.pgen.1004395)
Supplement: Text S1 — Alignment of L1 ORF1 sequences. Protein alignment of the homologous region of ORF1, amino acids 123–321, bp 1273–1869 of L1rp (GenBank accession number AF148856), including the reconstructed megabat L1 lineage 1 (L1-2_PVa), megabat L1 lineage 2 (L1-1_PVa), 26 RepBase-reconstructed L1 consensuses and four L1s reconstructed by us as described in Materials and Methods. ‘Conserved sites’ are the conserved amino acid sites among the surveyed species excluding the megabat L1s. L1rp is not shown in the alignment but shares the same nucleotide and amino acid coordinates with L1HS. (PDF) [file pgen.1004395.s006.pdf]

```
                           ---------+---------+---------+---------+---------+---------+---------+---------+---------+---------+
                                    10        20        30        40        50        60        70        80        90       100
                           ---------+---------+---------+---------+---------+---------+---------+---------+---------+---------+
Conserved sites            XXXXXXXXXXXXXXXXXXXXXXXXXXXXXXXXXNXRXXGXXEXXXXXXXXXXXXXXXXXXXEXXXXXXXXXXXXXXXXXXXRXPXXXXXXXXXXXRXXXXXX
lineage 1(L1-2_PVa)        DRNLEINQKEEERNRRMKNNEREIQELADTIRRGNIRIMGIIE-GEEKEQGLESIFRQIVDENFPNLRNEL-ELGIQEVNRTPNYLNPKRPSPRHIVLKL
lineage 2(L1-1_PVa)        DKVAEYTQSEQQKEKRIXKNEDSLRDLWDNMKRKNIRIIGVPE-EEEREQGIENLFEEIMTENFPNLAKEL-DIQVQEAQRVPNKRNSNRPTPRHIIIKM
L1-BT                      DRMVEINESERIKEKRIKRNEDNLRDLQDNIKRYNIRIIGVPE-EEDKKKDHEKILEEIIVENFPKMGKEI-ITQVQETQRVPNRINPRRNTPRHILIKL
L1-1_LA                    DQGINTNIAEKKSDKRIKKNEETLRIMWDSIKKDNLRVIGVPE-QGGGTENTEKIVEELLTENFPDIMKDE-RISIQDAHRTPFKIDPKRKTPRHIIIKL
L1-1_Vpa                   DRATESTHSEELQDKQIKNNDNSIRDLWDNIKRPNLRIIGVPE-GEEGSKGIEKVFEEIMTENFPNLKKES-DIQVQEAQRVPNRKNPNRPTPRHIIIKM
L1-1_DN                    DSTSEIKQIVEXVDKKIEKIQLGLRDLNDNAKRSNIRIIGIPE-GEEKGKGSEGVLQEIMAENFPNLLKET-DVHIQEAQRTPLVINPNRPTPRHILVKL
L1-1_Cpo                   DRIAASEQERKDLLKITRNQETTIQQLQDDAKKNNIRMIGINEKEGDNIKDVKRIFREVIAENFPSMRSET-DIRISEAYRTPNSHNQNKTTPRHIIITI
L1-1_SSc                   DRLVEITDAEQKREKRLKTNEESLRELWDNVKRTNIRIIGVPE-GEEREKETEKIFQEIIAENFPNMGKES-LTQIQEAQRVPYKINPRRNTPRHILIKL
L1-1B_Cho                  DDRMENERTKERMGKKIEKIEMDLRDMIDKIKRPNIRLIGVPE-GEEKGKGLERVFKEIVGENFPNLLHNI-NTQSINAQRTPNRINPNKPTPRHILIRL
L1-2_EC                    DRQAEWRQTEEERELRIKKNEENLREIMDSMRSKNIRIIGIPE-NMEKENGAESVLNEIIEENFPNLGING-EMCVEEGFRSPRFVNVKRPTARHIIVKL
L1MAB2_ML                  DKEAKHTQTVLQMEKKIKRQEESLRELWDNMKRNNIRIIGVPE-QQKDEHGLENLFEEIISENFPEVGKK----KVTQAQRVPNKVNPKRPTPRHIIITM
L1-1_Str                   DKVYQLEKSIVNTEKMLKSHEQSIQEIWDVXKKPNLRVIGIEEGTEIQTKGMDSILNEIIVENFPEMKDGM-DCQILEAYRTPNIQNHNRPTPRHIIMKI
L1-1_MD                    QELIKQSQNTKKLEENIKYLTDKVIDLENRGRRENLRIIGLPE-KPEINTKLDMVIQDIIKENCPEILEQGGNTSTDRAHRTPSTLNPQKTTPRNVIAKF
L1-1_TS                    DQNIEITQTLKNTENKLKKTEQNLQEMSDYLKRPNLRIIGLPEAERETETTLEQTFHEIIQENFPYLISDA-KIQTQEIQRTPARQQMRRPTPRHIIIRL
L1-1_OP                    DTQNEHTQFIKQLETSLNKANKTIQEMKDNLRKSNIRIIGLPE-GAEKESGMQMVLDEIIQENFQNTWNMN-PAQIQDGQRTPSRYDPKRSSPRHMVLKF
L1_RN                      DSIEIIDSTVKDNVKRKKLLVQNIQEIQDSMRRSNLRIIGIEESEDSQLKGPVNIFNKIIEENFPNLKKEI-PIDIQEAYRTPNRLDQKRNTSRHIIVKT
L1A_Mim                    DNTLQLNKSVTEIEQRNKRKEQSLQELWDYVKKPNVRVIGLPEGEEDNTQGLDKLFEDIIEENFPGLAQNL-DIQVQEAQRTPGRFNANRKTSRHAVIRL
L1-1_Cja                   DQLNEIKREGKIREKSAKRNEQSLQEMWDYVKRPNLRLIGVPECDEENESKLENTLQDIIQENFPNLARQA-NIQVQEIQRTPQRYSSRRATPRHIIVRF
L1-1_EE                    DELETTKKEVRDLKKRLRDAENNNRVLWDDFKRNNIRIIGLPE-EEREG-EEESILQAIIAENFSSLDNTK-DIKIQEAQRVPNRINPDLKTPRHVILRL
L1-1_Tbel                  DKTSDLDKSIKKIEKTTMKNEDNIRGILDTIKRPNIRIIGIPE-EEENNKGLENLFHEILEENFPNLERHS-NIQTQEIQRTPSRINPRRSSPRHIIARL
L1-1_Pca                   DRATDASVYEQKSEKRNKKNEETLRSMWDSIKRNNLRLIGVPE-QGETSENTESIVAELLKENFPEIMKEE-NIDISDAYRTPPNIDLKRKTPRHIIIKL
L1A_OC                     DREQERIQSDQRKEEEIRNLKHIVGNLQDTIKKPNIRVLGVPE-GMEREKGLEGLFSEILAENFPGLEKDR-EILVQEAHRTPNKHDQKRSSPRHVVIKL
L1HS                       DEMNEMKREGKFREKRIKRNEQSLQEIWDYVKRPNLRLIGVPESDVENGTKLENTLQDIIQENFPNLARQA-NVQIQEIQRTPQRYSSRRATPRHIIVRF
L1-2_Dor                   DSLDIIEKEHTTIQDKSKKYDRSIQHLEDTIRRPNLRIMGVEEHLETEVNGLGNLFNRILAENFPNIQKDR-PIQIQEAFRTPNRPDQNRTSHRHIIIHT
L1-Y_CF                    DKLIAKRETEEKRDKQLKDHEDRLREINDSLRKKNLRLIGVPE-GAERDRGPEYVFEQILAENFPNLGRET-GIQIQEIERSPPKINKNRSTPRHLIVKL
L1-1A2_Sar                 DELQAAYRQQQXMGRDLKIALGRIRVLGDXFKRNNIRIIGLPE-GQGTNPNEKATVKKIIAEKFPELDN-A-GIQIQGARRVPAKRDPNRKTPRHIIVTM
L1-1_ET                    DTQTDLSKRERQSDRKIKETEDSLRSMTDAMKRNNIRIIGLPE-HNTTHKSTAKIAKEFLEENFPTLTREN-QALIQEAERTPARLDPKKNTPRHIIVKI
L1-1_AMe                   DGLVEEKTKIEAGLKKIHAHECRLREITDSMKRSNVRIIGIPE-GVEKNRGLEEIFEQIVAENFPNLARET-SIRVQEAERTPSKLNQDKPTPRHVIVQF
Meug                       QESVKQNLKNEKIEENLKYLIGKTTDLENRSRRENLRIIGLPE-THDEEKSLDNIFQEIIKENCPEVLDSEGKIVIERIHRSPPERDPKLKTPRNIVAKF
Fcat                       DKVMEKEEAEKKRDKKIQEYEGKIRELSDTLKRNNIRIIGIPE-EEERGKGAEGVLEEIIAENFPELGKEK-GIEIQEAQRTPFRRNLNRSSARHIIVKL
Mmul                       DQMNEMKREEKPKEKRRKRNEQSLQEVWDYVKRPNLRLIGVPESEGENGTKLENTLQDIIQENFPNLVGQA-NIQIQEIQRTPQRYSSRRATPRHIIARF
Pham                       DQMNEMKREEKPKEKRRKRNEQSLQEVWDYVKRPNLRLIGVPESEGENGTKLENTLQDIIQENFPNLVGQA-NIQIQEIQRTPQRYSSRRATXRHIIARF
```

```
                           ---------+---------+---------+---------+---------+---------+---------+---------+---------+---------+
                                  110       120       130       140       150       160       170       180       190       200
                           ---------+---------+---------+---------+---------+---------+---------+---------+---------+---------+
Conserved sites            XXXXXXXXXLXXXXXXXXXXXXXGXXXRXXXDXXXXTXXXRXXWXXXXXXXXXXXXXXPRXXXPAXXXXXXXXXXXXFXXXXXXXXXXXXXXXXXXXXXXXX
lineage 1(L1-2_PVa)        SKINDKDRILRAAREKKTVTYKGKPIRLSSDFSAQTLQARKEWNQIFKLLSERNYQPRIMYPAKLSFRYEGEIKTFPDIQKLREFSTTRPALQEILKGVS
lineage 2(L1-1_PVa)        PKVKDKERILKAARERKLVTYKGTPIRMSADFSMETLQARREWQEIFKVMKNKNLQPRILYPARLSIKMEGEIKSFPDRKKLKEFITNKPALQEMLKGLL
L1-BT                      TTIKHKEQILKAAREKQQITHKGIPIRITADLSIETLQARREWQDILKMMKENNLQPRLLYPARISFKYEGEIKSFSDKQKLREFCTTKPALQQILKDIL
L1-1_LA                    AKTKDKQKILKAAREKRKVSFKGESIRISSDYSAETMQARREWDDIYRTLKEKNCQPRIIYPAKLSLKYEGEIKIFTDKHKFREFAKTKPKLQEILKDIV
L1-1_Vpa                   ARVKDKEMILKAAREKQRVNYKGTPIRLSADFSTQTLQARREWQDIFKALNEKKMQPRILYPARLSFRIEGEIKSFTDKKKAAGV.
L1-1_DN                    SNAQDKEKILKAAREKKTITYKGSSIRLSADFSSETMEARRQWYDIVKVLKEKNFQPRILYPAKLAFKHDGEFKIFTDKQKLKEYTNKKPPLQEILKGVL
L1-1_Cpo                   PEIQHKNRLLKAVREKRQITYKGKPIRITADFSAQTIKSRRAWSEVFQILKQNDFQPRLIYPAKLSFKIDGEIRYFHDKEQLKNFMNTKPTLQKILKDSL
L1-1_SSc                   TKIKDKEKILKAAREKKQITYKGTPIRLSADFSTETLQARREWHDILNVMKGKNLQPRLLYPARLSFRFEGEIKTFTDKQKLREFSNTKPALQQILKELL
L1-1B_Cho                  SNTEEKEQVLKAAREKQFTTYKGNNIRLSSDYSAATMEARRQWHDIFKILREKNCQPRILYPAKLSFKFEGELKFFTDKQMLRDFANKRPALLEILKGAL
L1-2_EC                    ANRNDKERILREVRKKKRITYKGAPIRLSADFSTETLQARREWSDIFKALKDKNLQPRILYPARISFRYEGEIKSFPDKQKLREFVTKSPPLQEILKKAL
L1MAB2_ML                  ANVQDKERILQAARERRKVTYKGSPIRLSNDFSTETHQARKEWTEIYKVMQSKGLNPRILYPARLSFKIEGEIRSFTDKKGLREFITTKPAMQEMLKGLV
L1-1_Str                   ANIQDKERILKATREKRQITFRGKPIRLTTDFSXQTLKARRSWNNVFQTLKNNGFQPRILYPAKLSFRFDNEIKIFHDKQKLKEFAARKPALQSILSKIL
L1-1_MD                    QSYQTKEKILQEAR-KRQFRYKGMPIRVTQDLASSTLNDRKAWNMIFRKARELGLQPRISYPAKLTIYFQGKVWAFNKIEDFQLFAKKRPELCGKFDTEN
L1-1_TS                    NKVGTKEKILKAAREKGQITYHGRPIRIAADLSAETLQARRAWSPIFKVLKDKQFQPRITYPAKLSFISEGELKSFPDIQSLRTYAATKPSLHETLKKVL
L1-1_OP                    HSNEDKERILRQVRSREKITYRGKPIRITADFSEETLQARREWTKIFQILNQNNCQPRILYPAKISFVFENEIKYFHSKEKLEEYASTKPALQNLLRNAL
L1_RN                      PNAQNKERILKAVREKGQVTYKGRPIRITPDFSPETMKARRSWTDVIQTLREHKCQPRLLYPAKLSINIDGETKIFHDKTKFTQYLSTNPALQRIINGKA
L1A_Mim                    TKVSTKEALLRAVRQKKQVTYKGKPIRITSDFSNETLQARRDWGPILTLLKQNNAQPRILFPAKLSFVYEGEIKTFSDKQRLREFTKTRPALQEVLKTAL
L1-1_Cja                   TRVEMKEKMLRAAREKGRVTHKGKPIRLTADLSAETLQARREWGPIFNILKEKNFQPRISYPAKLSFISEGKIKSFANKQVLRDFVTTRPALQELLKEAL
L1-1_EE                    ERNKDKERILKAAREKQRVTYKGKPIRLAADFSIQTLQARREWQDIYRVLNEKGFQPRILYPARLSFRLDGSIKTFSDKQQLKEATITKPALKEVLKGLL
L1-1_Tbel                  AKTTDKDKILKLARGKRQVTYKGHPIRLTSDLSAETMQERKEWGSIVKVLSEKQFQPRILYPAKLSFIWEKKIKTFSNKQNLKEFTNTRPALQEVLKGVL
L1-1_Pca                   NKMKHKQQLLKAARLKAKLTFRGKPCRLSSDFSAETMLARRQWHDTFKALKEKNFQPRIIYPAKLSFKYENEIKTFPDKQKLRDFVKTKPKLQEILKEVL
L1A_OC                     STVKHKEKILKCAREKRQITLRGSPIRLTADFSSETLQARREWRDIAQVLRENNCQPRILYPAKLSFVNEGEIKTFHSKQKLKEFVATRPALQKMLKDVL
L1HS                       TKVEMKEKMLRAAREKGRVTLKGKPIRLTADLSAETLQARREWGPIFNILKEKNFQPRISYPAKLSFISEGEIKYFIDKQMLRDFVTTRPALKELLKEAL
L1-2_Dor                   GSTQTKEKILKAVKEKRVITYKGKPIRITSDFSAETIKARRAWNDVCHALKTNNYQPRILYPAKLSFITEGQIKTFHSKEKLKQYISTKPALQKILKDVL
L1-Y_CF                    ANSKDKEKILKAARDKKSLTFMGRSIRVTADLSTETWQARKGWQDIFRVLNEKNMQPRILYPARLSFKMEGEIKSFQDRQQLKEYVTSKPALQEILRGPL
L1-1A2_Sar                 MDVTDRDTILQAARSKKEIAYKGAPLRFTADLSEETLQARRQWWDIVKKLNEMNASPRILYPAKLSFKLEGIIHYFGDKQQLRNFIDSKPNLKEGLKGLL
L1-1_ET                    SNFEEKEKILRAARERKTVTYKGAQVRICSDLSADTMKRRREWSNIFQKLKEKNASPRILYPAKLSIKIDGEIRVFQDKERLKEYAARHPSLRKILADSL
L1-1_AMe                   ANIRSKDTVLKAARAKKFLTYQGKGIRITSDLSTETWNERKAWGGIFKALSEKNMQPRILYPAKLSFRIDGEIKTFQNRQSLTNFVTTKPALQEILRGAL
Meug                       QSYQVKEKILQAAR-KKQFKYQGHTVRITQDLAASTLKDRKNWNPIFRKAKELGLQPRINYPAKFSITFQARRKSFNEIRDFQSFLTKTPELNRQFDLQM
Fcat                       AKYKDKEKILKAARGKRALTYKGRPIRLVTDLSFETWQARKNWHEIFRVLDRKNMQPRILYPASLSFRIEGEIKVFPNKQKLKEFVTTKPALQEILRGTL
Mmul                       TKVEMKEKILRAAREKGRVTHKGKPIRLTADLSAETLQARREWGPIFNILKEKNFKPRISYPAKLSFISEGEIKSFTDKQMLRDFVTTRPALQETLKEAL
Pham                       TKVEMKEKILRAAREKGRVTHKGKPIRLTADLSAETLQARREWGPIFNILKEKNFKPRISYPAKLSFISEGEIKSFTDKQMLRDFVTTRPALQETLKEAL
```
