# Supplementary material for: Reviving the Dead: History and Reactivation of an Extinct L1
Source: PLoS Genet. 2014 Jun 26;10(6):e1004395. doi: 10.1371/journal.pgen.1004395 (PMC4072516; doi:10.1371/journal.pgen.1004395)
Supplement: Text S2 — Alignment of L1 ORF2 sequences. Protein alignment of the homologous region of ORF2 spanning the full length L1rp ORF2 (bp 1987–5814, GenBank accession number AF148856), including the reconstructed megabat L1 lineage 1 (L1-2_PVa), megabat L1 lineage 2 (L1-1_PVa), 26 RepBase-reconstructed L1 consensuses and four L1s reconstructed by us as described in Materials and Methods. ‘Conserved sites’ are the conserved amino acid sites among the surveyed species excluding the megabat L1s. L1rp is not shown in the alignment but shares the same nucleotide and amino acid coordinates with L1HS. (PDF) [file pgen.1004395.s007.pdf]

```
                              ---------+---------+---------+---------+---------+---------+---------+---------+---------+---------+
                                       10        20        30        40        50        60        70        80        90       100
                              ---------+---------+---------+---------+---------+---------+---------+---------+---------+---------+
Conserved sites               XXXXXXXXXXXXXNXNGXNXXXKRXRXXXWXXXXXXXXXCXQEXHXXXXXXXXXXXXGWXXXXXXXXXXXXXGXXXXXXXXXXXXXXXXXXDXXGXXXXX
lineage 1(L1-2_PVa)           MAIRRPHISIITLNVNGLNSPIKRHRVAEWIKKQNPTICCLQETHLSSKDKYRLKVKGWKMIFQANGIQRKAGVAVLISDEIDFKIKKVKKDTEGHFIMI
lineage 2(L1-1_PVa)           KMAVTMYLSVITLNVNGLNAPIKRHRVTEWIRKQDPCICCLQETHFRSNDTHRLKVKGWKKIFHANGNEKKAGVAILISDKIDFKTKAIIRDKKGHYIMI
L1-BT                         M-ATGTYLSVITLNVNGLNAPTKRQRLAEWIQKQDPYICCLQETHLKTGDTYRLKVKGWKKIFHANRDQKKAGVAILISDKIDFKTKAVKRDKEGHYIMI
L1-1_LA                       MTALKTYLSIITLNVNGLNAPIKRQRVTDWIKKHDPSICCLQETHLRLRDTNKLKLKGWKKIYQANNKQKRRGVAILISDKIDFRLKSTTKDKEGHYIMI
L1-1_Vpa                      M-AINTHLSLITVNVNGLNAPVKRHRVADWIIKQEPSICCIQETHFREKDTYRLRVKGWKRIFHANGKAKKAGVAVLISDKIDFKTKAIKKDKEGHFIMI
L1-1_DN                       MANTNNSLKVITLNVNGLNSPIKRFRLGHWIRKYDPSVCCLQETHLRPRDSWRLKVNGWKTIIQANNNQKKAGVAILISDKIDFKCETIVRDKEGYYILV
L1-1_Cpo                      MPTINQHLTVITINVNGLNAPIKRNRLAEWIKKQNPTICCLQETHLTQKDTHRLKVKGWKTILHAAGIQKKAGVAILFADNVNFKPTMTIKDKEGHYILV
L1-1_SSc                      M-AIRTYISIITLNVNGLNAPTKRHRLAEWIQKQDPYICCLQETHFTSRDTYKLKVRGWKKIFHANGDQKKAGVAILISDKIDFKMKNIFRDKEGHYIMI
L1-1B_Cho                     MADSRNAFTVITLNVNGLNSPIKRYRLAEWIKKYEPSICCIQETHLRHRDTKKLKVKGWKKIFHASYSQKKAGVAILISDKIDFKCKDVMRDKEGHYILI
L1-2_EC                       LTALSPHASIITLNVNGLNSPIKRHRVAKWIKEQDPTICCLQETHLSPKDKHRLRVKGWRTILQANSKEKKAGVAILISDQVDFKIRQVKRDTEGQYIMI
L1MAB2_ML                     M-ATNKYLSIITLNVNGLNAPTKRHRVAEWIKKHDPYICCLQETHLIRRDSHRLKVKGWKNIFHANGKEKKAGVAILISDKIHLKVKAITRDKEGHFIIL
L1-1_Str                      MTGSTNHISIVTLNVNGLNSPIKRHRLVTWIKKTNPTICCLQETHMIGKDIHRLKVKGWEKSYHSHGPRKQAGVAILISNKINFKPKLIKKDKEGHYILL
L1-1_MD                       M-PGSPQMTIITLNVNGMNSPIKRRRIAEWIRIQNPTICCLQETHMRRVDTHKVRIKGWSKTFWASTDRKKAGVVIMISDKANAKIDLIKRDREGNYILL
L1-1_TS                       MIGTNSHISIISLNVNGLNAPLKRHRMTKWIKYHQATIYCLQETHLTRKDIHRLKVRGWETNFQANGTQKKGGVAILISDKIPFKLSKIKKDTEGHYIMI
L1-1_OP                       MAGQNHNLSILTLNTNGLNSPIKRHRLTEWIIKQHPTICCLQETHLTRRDSKKLKVKGWKQIFHANGREKKAGVAVLISDDVDFNLTHIKKDREGHYILV
L1_RN                         ITGSNNHYSLISLNINGLNSPIKRHRLTNWIRNEDPAFCCLQETHLRDKDRHYLRVKGWKTTFQANGQKKQAGVAILISNKINFQLKVIKKDKEGHFIFI
L1A_Mim                       MISNLPYLSVLSINVNGLNSPLKRHRLAEWIRKYRPSICCLQETHLTCKDAYRLKIKGWRSIFQANRSQKKAGVAVLISDDLVFKPTKVVKDKEGHYIMV
L1-1_Cja                      MAVSNSHITILTLNVNGLNAPIKRHRLANWIKSQNPSVCCIQETHLTCKDTQRLKIKGWRKIYQANGEQKKAGVAILVSDKIDFKATKIKRDKEGHYIMV
L1-1_EE                       M-ALKYLQSLISINVNGLNSPIKRHRVGRWIRKHNPTICCLQETHLTQQDKHRLKVKGWKTIIQANGPQKRAGTAILISDMIDFKIDKIKKDRNGHYLML
L1-1_Tbel                     MIGSGMEISIITLNVNGLNSPVKRHRLAEWIRKQKPSICCLQETHLMDKDIHRLKVKGWKKIFHANGNQKKAGVAILISDKIDFKTKTVRRDKEGHYIIV
L1-1_Pca                      MAAVNTYLSIITLNVNGLNAPVKRHRVSSWIKRHDPSICCLQETHLRPKDRNKLKIKGWKNIYQANNEQRRAGVAILISDKINFNVKSTIVDKDGHYLLL
L1A_OC                        MAGQSYHLSLVTLNVNGLNCPVKRHRLADWVKEQNPSICCLQETHLSNKDPYRLKVKGWKKIYHANRNEKRAGVAILISDNINFTTKTVKRDKEGHYIMI
L1HS                          MTGSNSHITILTLNINGLNSAIKRHRLASWIKSQDPSVCCIQETHLTCRDTHRLKIKGWRKIYQANGKQKKAGVAILVSDKTDFKPTKIKRDKEGHYIMV
L1-2_Dor                      MAGTPKYLSVLSLNVNGLNSPIKRHRLSSWIKKQDPSICCLQESHLARKNKHIFNVKGWNKIYQANGPHKQAGVAILISDKIDFKLKFVRRDKEGRYILI
L1-Y_CF                       MMTLNSYLSIVTLNVNGLNDPIKRRRVSDWIKKQDPSICCLQETHFRQKDTYSLKIKGWRTIYHSNGPQKKAGVAILISDKLKFTPKTVVRDEEGHYIIL
L1-1A2_Sar                    M--AQNPMTIISLNVNGLNSPIKRHRLAKWIQRLNPTFCCLQETHLNSQNKHRLKVKGWKTILQANNSLKKAGVALLVSDNIDFRLKKIRRDSEGHFLLI
L1-1_ET                       MTTMNPQIVIITLNSNGLNSYIKRKRLEDWLRKHNPSICCLQETHLKRTDKNMLRIKGWQKVYQXNSNSKKAGVAILISDXIDFKIQTIKRDKEGHYIML
L1-1_AMe                      MASLKSYLSIISINLNGLNSPIKRHRVADWIKRHDPSICCLQETHFEPKDAFRLRVRGWSTIFHANGPQKKAGVAILISDRLDFKLEAIERDTEGHYIIL
Meug                          RLEGNTQIVIITVNVNGMNSPIKRRRIAEWIKSHNPTICCLQETHLKRGDTHRIKVKGWSRIYCASAHVRKAGVAILISDKAKAEIDLIKRDKDGNYILL
Fcat                          M-TLNPYLSIITLNVNGLNAPTKRHRVSEWIKKQDPSICCLQETHFRPEDTFRLRVRGWRTIYHATGSQKKAGVAILISDKLDFKLKAVTRDEEGHYIIV
Mmul                          MAGSSSHITILTLNVNGLNAPIKRHRLANWIKSQDPSVCCIQETHLTRRDIHRLKIKGWRKIYQANGEQKKAGVAILVSDKTDFKPSKIKRDKEGHYIMV
Pham                          MAGSSSHITILTLNVNGLNAPIKRHRLANWIKSQDPSVCCIQETHLTRRDIHRLKIKGWRKIYQANGEQKKAGVAILVSDKTDFKPSKIKRDKEGHYIMV
```

```
                            ---------+---------+---------+---------+---------+---------+---------+---------+---------+---------+
                                    110       120       130       140       150       160       170       180       190       200
                            ---------+---------+---------+---------+---------+---------+---------+---------+---------+---------+
Conserved sites             XGXXXXXXXXXXNXYXPXXXXXXXXXXXXXXXXXXXXXXXXXXXGDXNXXLXXXDXXXXXXXXXXXXXXXXXXXXXXXXXDXXRXXXXXXXXXXXFXSXXHXX
lineage 1(L1-2_PVa)         KGIMHQEDITLINIYAPNQGAPKYVKQLLTELKGETDQNTIVVGDLNTPLSDMDRSSKQKINKEITSLNDTLDQLDIIDIYRAFHPKTAAYTFFSSAHGT
lineage 2(L1-1_PVa)         KGSINQEDITLVNIYAPNIGAPTYIKQLLTDIKGEINSNTLIVGDFNTPLTPMDRSSRQRINKETMGLKDTLEQMDLIDIFRAFYPKAAKYTFFSSAHGT
L1-BT                       KGSIQEEEITIINIYAPNTGAPQYVRQMLTSMKGEINNNTIIVGDFNTPLTPMDRSTKQKINKETQTLNDTIDQLDLIDIYRSFHPKTMNFTFFSSAHGT
L1-1_LA                     KGTIDQEDITILNIYAPNDRAARYINQILTELKSEIDTSTIIVGDFNTPLSEKDRTSSKKLNRDTEDLITTINQLDLIDLYRTLHPTAAKYTFFSSAHGT
L1-1_Vpa                    KGVIQDEDITLVNIYAPNIGAPKYIQELLTEIKGDIDGNTIIVGDFNTALTSLDRSSRQKINKATEKLNTTIEKLDLVDIFRALHPPKIEYTFFSSAHGT
L1-1_DN                     KGKICQEDRTIINIYAPNKGASKYVRQTLEKLSERIDASTIIVGDFNTPLSTLDRTSQKRITKETKHLNSILEELDLIDIYRSLHPNTAGYTFFSSAHGS
L1-1_Cpo                    RGKLQEEEITILNIYAPNSRAPSYIKQLLTEMKTQIISNTIVTGDLNTPLTPRDRSTRQKMSKEITELNHTCEQMGLIDIYRMFHPTTSEYTFFSAVHGS
L1-1_SSc                    KGSIQEDXITILNIYAPNTGSPQYIRQLLTTLKGEIDNNTIIVGDFNTPLTAMDRSTRQKINKETQALNEALNQMDLIDIYRTFHPKATEYTFFSSAHGT
L1-1B_Cho                   KGAIQQEEITIINVYAPNQGATKYMRQTLAKLKEAIDVSTIIVGDFNTSLSPIDRSTRQKTNKEIENLNNLINEFDLTDIYRTLHPKSPGYTFFSSAHGT
L1-2_EC                     KGTLHQEEITLINIYAPNTGAPRFIKQLLTDLKEDVKNNTIIVGDLNTPLTSMDRSSRQKINKEIVELNEKLKQLDLIDIYRSLHPEGAEYTFFSSAHGT
L1MAB2_ML                   KGSIQQEDITLVNIYAPNAGAPTYIRKLLEDIRGEIDNNTIIVGDFNTPLTSLDKSARQTISKETAILNDSLDQMDLIDIFRTLHPTAAEYTFLSGAHGT
L1-1_Str                    KGTIHQQDITIINLYAPNNGAATFIKQTLLKFKSQIDHNTIITGDFNTPLSPLDRSSRQKLNKETIELNSTINNLDLTDIYRIYQPSSSGYTFFSAAHGS
L1-1_MD                     KGTLDNEEISLINMYAPNNIAPKFLMEKLGELKEEIDNKTILVGDLNQPLSNLDKSNQKINKKEVKEVNEILEKLELIDIWRKINRDKKEYTFFSAPHGT
L1-1_TS                     KGSLHQQEISILNIYAPNIGAPTFIKQLLGKLKKDIDSNTIITGDFNTPLTTLDRSSGQKISKEIRNLNETLDQMDLIDTYRTLHPKTTEYTFYSSPHGT
L1-1_OP                     KGLIHQEVITIVNIYAPNSNAPSYVKQLLTDLRGDIDMHTIIVGDLNTPLTTIDRSTKQKLNKETTELIQTIEQLDLVDIYRIFYPKATDYTFFSAVHGT
L1_RN                       KGKIHQDELSILNIYAPNTRAPTYVKETLLKLKTHIAPHTIIVGDFNTPLSSMDRSWKQKLNSDVDRLREVMSQMDLTDIYRTFYPKAKGYTFFSAPHGT
L1A_Mim                     KGTVQQEEITILNIYAPNLGAPRFIKQTLLELSKWINSNSIIAGDFNTPLTARDRSSKQKINKEIMDLNKTLEQLGLTDIYRTFYPKSTEYTFFSSAHGT
L1-1_Cja                    KGSIQQEELTILNIYGPNTGAPRYIRQVLNDLQRDLDSHTIIVGDFNTPLSILDRSTRQKINKDIQDLNSDLEQANLIDIYRTLHPKSTEYTFFSAPHHT
L1-1_EE                     RGSVNQEDLTIINIYAPNEKPSKYIKLLLKELQQYINSNTIIVGDFNTPLSQLDRSSRKKISKDIRELNEEIDKLELLDIFRVIHPKKLEYTFYSNPHGS
L1-1_Tbel                   DGVIQQEDITLINIYAPNSGAPKYVKERLLELKGDIGKNTIIVGDLNTPLSPIDRSSRQKINREIQDLNDTINGMGLTDIYRTFHPTSTTYTFFSPRHGT
L1-1_Pca                    KGSVHKQDLTILNIYAPNVRAPKYVEETLTTLKRELDDSTIIVGDFNTPLSLLDRSSRKKLSKDTEDLIDTINQLNLIDIYRTFHPSADNYTFFSSAHGT
L1A_OC                      KGSIQQEDITIINVYAPNYRAPVYLKDLLTDLKGDLDPNTIVLGDFNTPLSEIDRSTGQKINKDTVDLNDTIAQMDLTDIYRTFNPTAKDFTFFSAVHGT
L1HS                        KGSIQQEELTILNIYAPNTGAPRFIKQVLSDLQRDLDSHTLIMGDFNTPLSTLDRSTRQKVNKDTQELNSALHQADLIDIYRTLHPKSTEYTFFSAPHHT
L1-2_Dor                    KGSLLQEDITILNIYSPNNGAPKFIKETLLSLKSHIDPKTLIVGDFNTPLSPLDRSTRQKLNRETADLNSCIDQLDLTDIYRTFHPAASEYTFFSAAHGT
L1-Y_CF                     KGSIQQEDLTILNIYAPNVGAAKYINQLLTKVKKYLDNNTLILGDFNLALSILDRSSKQNISKETRALNDTLDQMDFTDIYRTLHPNSTEYTFFSSAHGT
L1-1A2_Sar                  KGYVQQEEITLLNVYAPNEGPAKYLQQLLTDLKKDIASNTIVVGDFNTALSPLDRSRRLKLTKEILALKEEIEERGLIDLYRALYPQKKEYTFFSSAHGT
L1-1_ET                     KGSVNQEAXSILNIYAPNNGAANFVKQTIKKMKKEITDSTIIVGDFNTPLSEKDRSLGKKLSKDAIELNNVIRQQGLIDIYRAFHPNAKGFTFFSSPHGS
L1-1_AMe                    KGSIQQVDMTIINIYAPNRGAARYTSQLLTKIKRHIDKNTVIVGDLNTPLSEIDRTPWQKLSKESKALNAILDELDLIDIYRTLHPRTKEYSFYSNAHGT
Meug                        KGTIDNEAISLLNMYAPSGIASRFLEERLGELKEEIDSKTILVGDLNLPLSELDKSNLKINKKEVKEVNKTLDKVDMIDLWRKLNGNRKEYTFFSAVHGT
Fcat                        TGSIHQEELTIINVYAPNTGAPKYIKQLLINISNLIDKNVVIAGDFNTPLTEMDRSSRHTVNKETRALNETLDQMDLTDIFRTLHPKATEYTFFSSAHGT
Mmul                        KGSIQQEELTILNIYAPNTGAPRFIKQVLRDLQRDLDSHTIIMGDFNTPLSTLDRSTRQKVNKDIQELNSSLQQADLIDIYRTLHPKSTEYTFFSAPHRT
Pham                        KGSIQQEELTILNIYAPNTGAPRFIKQVLRDLQRDLDSHTIIMGDFNTPLSTLDRSTRQKVNKDIQELNSSLQQADLIDIYRTLHPKSTEYTFFSAPHRT
```

```
                    ---------+---------+---------+---------+---------+---------+---------+---------+---------+---------+
                             210       220       230       240       250       260       270       280       290       300
                    ---------+---------+---------+---------+---------+---------+---------+---------+---------+---------+
Conserved sites     XXXIDHXXXXXXXXXXXXXXXXXXXXXXXDHXXXXXXXXXXXXXXXXXXXXWXXXXXXLXXXXXXXXXXXXXXXXXXXXNXXXXXXXXXXWDXXKAVXRGXXI
lineage 1(L1-2_PVa) FSRIDHILGHRDSLNKYKRVEIIPTIFSDHNALKLEINCKKKSGRTTNTWRLNNMLLKNNWVREEIKREIKRYIETNDNDSTTYQNFWDTAKAVIRGKFI
lineage 2(L1-1_PVa) FSRIDHMLGHKSSLNKFKKIEIISTVFSDHNGMKLEINYKKKTGKYTNTWRLNNMLLNNEWVINEIKEEIKRYLETNENENTTTQNLWDTAKAVLRGKFI
L1-BT               FSRIDHILGHKASLGKFKKIEIIPSIFSDHNAVRLDLNYRRKTIKNSNIWRLNNTLLNNQQITEEIKKEIKICIETNENENTTTQNLWDAVKAVLRGKFI
L1-1_LA             FSRIDHILGHKTNLCRVQNIEILQSIFSDHKAIKLEINNRKTREKKSNTWKMNNTLLKKDWVIEDIKEGIRKFIESNENENTSYQNLWDTAKAVLRGQFI
L1-1_Vpa            FSRIDHVLGHKRNLNKFKKIEIISSIFTDHNAMKLEINNRETKEKKRKAWRLNNMLLKKQWINEEIKAEIKKYLETNDNESTTTQNLWDTAKAVLRGKFI
L1-1_DN             FSKIDHMLGHKERLNEFRKIEIIQNNISDHSGVKLEICKGQRPRFHTTIWKLNSTLLEKQWVKEEISKEINDYLETNDNDNTTYQNLWDAAKAVLRGKFI
L1-1_Cpo            FSKIDHILAHRTYLNKCKRVEIIPCMLSDHSALKLEINDKRYCKNPANTWKLNNTLLSNQWVTEEIKEEIKQYLKENENADTTYRNLWDAMKAVLRGKFI
L1-1_SSc            FSKIDHILGYKSNLGNFKKIEIISSIFSDHNAIRLEINNKKKTAKNTNTWRLNNMLLNNQWITEEIKEEIKKYLAANDNEDTTLQNLWDAAKAVLRGKFI
L1-1B_Cho           FSRIDHMLGHKTSLNKFKKIEIIQSTFSDHNGIQLEVNNHQRLRKFTNTWRLNNTLLNNQWVKEEIAREIAKYIEMNENENTTYQNLWDAAKAVLRGKFI
L1-2_EC             FSRIDHMLGNKASLYKFKKIEIITSIFSDHSAIRLEINYKKKAEKGTKMWRLNNTLLNKQWIIEEIKEEIKKYLETNENDSMPYQLIWDTAKAVLRGKFI
L1MAB2_ML           YSKIDHILGHKQSLHKFKKIEIIKSIFSDHDGIMLEINYNKNNPKYSNTWKLNSMLLNIDWVTDDIKEEIKNILDTNDNENTTIQNLWDTMKAVLRGKFI
L1-1_Str            YSKIDHILCHRATLSKYKGVEIIPCTISDHNGMKLEINDKRRKEKSYTTWKMNNMLLNDQWVTEDIKEEIKKFLEXNDNTDTTYRNLWDTMKAVLRGKFI
L1-1_MD             FTKIDHTLGHRNIAHKCKKAEIMNAAFSDHKAIKIMISNGTWKTKSKTNWKLNNMILQNRLAKEEIIETINNFIKENDNGETSFQTFWDAAKAVIRGKFI
L1-1_TS             YSKIDHIIGHKSSISKFKRTEILPCTFSDHSGIKINIDTNKVPPKPTKTWTLNSMMLNNSWVNDDIKTEIKRYLETNENEETSYQNLWDALKAVIRGEFI
L1-1_OP             FSRIDHMIGHKANLNNFKKIGIIPCTLSDHHGMKLEISNSKCPRKYRNSWRLNNMLLNEQWIREEIKDEIKKFMETNENSDTTFQNLWDTAKAVLRGKLI
L1_RN               FSKIDHIIGQKTGLNRYRKIEIIPCVLSDHHGLKLVFNNN-KGRMPTYTWKLNNALLNDNLVKEEIKKEIKNFLEFNENEDTTYPNLWDTMKAVLRGKLI
L1A_Mim             FSKIDHILGHKENLKKFKKIEIIPCTFSDHSGIKLEINPNRNSHFYTKTWKLNNLLLNDYFVNEEIKTEIKKFYEENDNGETSYQLLWDTAKAVLRGKFI
L1-1_Cja            YSKIDHIIGSKSLLSKCKRTEIITNSLSDHSAIKLELRIQKLTQNRTASWKLNNWLLNVDWINNEMKAEIKKFFETNENEDTTYQNLWDTFKAVSRGKYI
L1-1_EE             FSRIDHMLGHKDSISQFKSTEIIPSIFSDHSGIKLTLNNQQKISNSPKMWKLNSTLLNNFWVKEEIKEEIKMFREFNENEDTSYQNIWDTAKAVLRGKFI
L1-1_Tbel           FSKIDHILGHKSSLYRFKGIETVPSIFSDHDGIKLKINLKKQPRKHTNTWKLNNLLLNDFFTTEEIKAEINKFMETNDNEXTSVQNLWDTAKAVIRGKFI
L1-1_Pca            FSRIDHVIGHNAMLNKIHTIELLQSIFSDHKAIKVEINSRKNIDRKWNSWKLNNILLKKTWVIEEIKDELKKFMDSNENENTSYQNLWDTAKAVLRGLFI
L1A_OC              FSRIDHILGHKASLSKFKRIRIIPCSFSDHKGMKLEISNSGIPRAYANTWRLNNMLLNEQWVIEEIKREIKNFLEVNEDNSTTYQNLWDAAKAVLRGKFI
L1HS                YSKIDHIVGSKALLSKCKRTEIITNYLSDHSAIKLELRIKNLTQSRSTTWKLNNLLLNDYWVHNEMKAEIKMFFETNENKDTTYQNLWDAFKAVCRGKFI
L1-2_Dor            FSKIDHILGHKANLSKFRNIEVIPCILSDHKGIKLELNTKSHQRKSGITWRLNNTLLNHEWVMEEIRKEIQMFMLFNKNEHTNYQLLWDTAKAVLRGKFI
L1-Y_CF             FSRIDHILGHKSGLNRYQKIGIVPCIFSDHNALKLELNHNKKFGRTSNTWRLRTILLKDKRVNQEIKEELKRFMETNENEDTTVQNLWDAAKAVLRGKYI
L1-1A2_Sar          FSRIDHALGHATYLNRINKIDIVPAIFSDHDALKIEVNRGQMQKTKSNTWKLNSSMLNNEWVRKEIKEEIKRYLETNENEDTSYQNLWDAAKAVLRGKFI
L1-1_ET             FSRIDHMLGHKSSLSKFKHIDIIQTSFSDHHAIKLEINKRTSRKARATNWRMNNDLLRHEWVQAQIRXDIRKFLETNENENTTYQNLWDTAKAVIRGSLI
L1-1_AMe            FSRIDHALGHKTGLSQYQKIEIIPCIFSDHNALKLELNHKEKPGRNSNTWRLRTILLKNDSINQEIKKQIKQFMETNENEYTTVQNLWDTAKAVLRGKYI
Meug                FTKIDHVLGHKNLTIQCRKVEIINASFSDHNALKITCNKRPWKEKPKINWKLNNLILKKGWVKEEIIETINNFIQENDNSETTYQNLWDTAKAVIRGSFI
Fcat                FSKIDHILGHKTALHKFTRIEIIPCILSDHNAMKLEINHRKKSGKPPKAWRLKNTLLTNEWVNQAIREEIKKYMETNENENTTIQTLWDAAKAVLRGKYI
Mmul                YSKIDHVIGSKALLSKCTRTEIITNCLSDHSAIKLELRTKKLNQNRSTTWKLNNLLLNDYWVHNEMKAEIKMFFETNENKDTTYQNLWDTFKAVCRGKFI
Pham                YSKIDHIIGSKALLSKCTRTEIITNCLSDHSAIKLELRTKKLNQNRSTTWKLNNLLLNDYWVHNEMKAEIKMFFETNENKDTTYQNLWDTFKAVCRGKFI
```

```
                               ---------+---------+---------+---------+---------+---------+---------+---------+---------+---------+
                                        310       320       330       340       350       360       370       380       390       400
                               ---------+---------+---------+---------+---------+---------+---------+---------+---------+---------+
Conserved sites                XXXXXXXXXXXXXXXXLXXXXXXXEXXXXXXXXXXXXXXXXXXXXXXXXXXEXXXXXXXIXXXXXWXXEXXXKIDXXLXXLXXXXXEXXXIXXXXXXXXXX
lineage 1(L1-2_PVa)            SLQAYLQKQERAQINNLTLHLKKLEKEEQMKPKVSRRKEIIKIRAEINEIENKKTIEKINATKSWFFEKVNKIDKPLARLTKEKRERTQINKIRDERGEI
lineage 2(L1-1_PVa)            AIQAYLKKQEKAQINNLTLQLKELEKEQQTKPKVNRRKEIIKIRAEINEIESRKTIQKINETKSWFFERINKIDRPLARLIKEKRERTQINKIRNERGEI
L1-BT                          AIQAHLKKQEKSQINNLTLHLKQLEKEEMKNPRVSRRKEILKIRAEINAKETKETIAKINKTKSWFFERINKIDKPLARLIKKQREKNQINKIRNENGEI
L1-1_LA                        SINAHIQKEERAKIRELSLQLEQIESEQQKNPSGTRRKQIIKIRAELNELENRKTIERINKAKSWFFEKINKIDKPLARLTKEIQERKQITRIRNEKDHI
L1-1_Vpa                       AIQAFLKKEEQSQINKLTHHLNQLEKEEQKAPKSSRRKEIIKIREELNTIEINKTIEKINQTKSWFFEKVNKIDKPLAKLTKKKKERAQISKIRKENGEI
L1-1_DN                        AINSYIKKEERAKIEELTAHLKELEKQQQSNPTGRRRKEITKIRAELNEIENKKALEKINKTKSWFFEKINKIDKPLARLTKKKREKMQIHKIRNEKGDI
L1-1_Cpo                       ALSSHIRKTERIQINNLMLHLKQLEKEEQVKPKAKRREEIIKIRAEINAIETKKTIQRINESKSWFFERINKIDKPLANLI.KREEKAQIHAIRNEKGEI
L1-1_SSc                       AIQAHLRKQEKAQINKLTLHLKQLEREEQTRPKVSRRKEIIKIRAEINEIETKKTIEKINETKSWFFEKINKIDKPLARLIKQKRERTQINKIRNEKGEV
L1-1B_Cho                      ALNAYIKKEERAKIKELMEQLKKLENEQQTNPKPSRRKEITRIKAEINDIENKKTIERINNTKSWFFEKINKIDKPLARLTKSKREKTHINKIMNEKGDI
L1-2_EC                        AIQAHLNKQEKSQISNLKAHLTELEKKEQMKPKVSRRREIIKIRAEINTIETKKAVERINETKSWFFEKINKIDKPLARLTKKKREKAQINKIRNERGEI
L1MAB2_ML                      ALQAYLKKQEKMVVNHLTLQLKELEREQQENPRVSRRKEIIKIRAEINDIETKKTIQKINETKSWFFERINKIDKPLARLTKKQRERSQINQIRNDRGEI
L1-1_Str                       SWSSFLKKRKNQQINELTLHLKNLEKEEQNNSKCSRRQEIIKIRAEINEIETKKTIEKIDKTKSWFFEKINKIDKPLAMLTKRRRERTQITNIRDEKGNI
L1-1_MD                        SLKAHINKQGRAEINQLEMQLKKLESDQIKNPQQKTKLEILKIKGEINKIESDRTIDLINKTRSWYFEKTNKIDKVLVNLIKKRKEEKQIHSIKDEKGDS
L1-1_TS                        SLQTHMRKMEGTEIDNLTSHLKKLEKQDHKNPNFSRRIQITKIKAQIQDIEDKKIIQKINETKSWFFERVNKIDGPLARLTKKKREKNQISTIRNTKDEV
L1-1_OP                        AIGAHVKAQERRQIQELNTHLQELEKQQQKSPTHNRKQEIIKTREEINQIEIKKTIHKINESKSWFFEKINKIDTPLARLTKKKQEKARINSIKDEKGNI
L1_RN                          ALSACRKKQERAYVSSLTAHLKALEQKEANTPRRSRRQEIIKLRAEINQVETKRTIERINRTKSWFFEKINKIDKPLARLTRGHRECVQINKIRNEKGDI
L1A_Mim                        SINAYNQKTRRSQIDNLMKRLKELEKEEQTNPKPSRRSEINKIKSELNEIENREAIQEINKTKSWFFEKINKIDTPLAKLTKSRKEKSLISSIRNKKGDI
L1-1_Cja                       AISAHMRRVERSKIDTLSSKLKELEEQDQKNSKPSRRQEITKIRAELKEIETRKTLQKINKSRSWFFEKINKIDRPLARLIKKKRENNQIDAIKNDKGEI
L1-1_EE                        AIQAHIRKQEKAQINSLIAHLKDLEEEQQRNPKATRRTEITKVRAEINNIENRKTIQKINESKCWFFERVNKIDKPLARLTKQKREKTQINRIVNERGDI
L1-1_Tbel                      AISAHKKRMERWQVDNLSSCLRELEKQQQTNPQNARRKEIIKVRAEINEIENKRTIQKINESKSWFFEKINKIDTPLARLIKKKKERNQINQICDETGNI
L1-1_Pca                       SINAHIWKEERNQIKELTLQLEQVEREQQRNPSGTRRNEIIKIRAELNELENRKTIEKIEKTKSWFFEKINKIDKPLAKLTKEKQERKQITQIRNERGVV
L1A_OC                         SIGAYIKKLERHQIDELSIHLKDLENLQQTRPKSSRRREIIKIREEINRIESRKTLQKISQTRSWFFEKINKIDTPLAQLTKKRREKTQINKIRDEKGNV
L1HS                           ALNAYKRKQERSKIDTLTSQLKELEKQEQTHSKASRRQEITKIRAELKEIETQKTLQKINESRSWFFERINKIDRPLARLIKKKREKNQIDTIKNDKGDI
L1-2_Dor                       ALSSYINKLERSETNNLMTHLNLLEKEQQDKPQSSRRKEIIKIKSELNELESKKTIERINKTKSWFFEKLNKIDRPLANLIRKRREHTQINKLRDENGNI
L1-Y_CF                        AIQASIQKLERTQIQKLTLHIKELEKKQQIDPTPKRRRELIKIRAELNEIETRRTVEQINRTRSWFFERINKIDKPLASLIKKKREKTQINKIMNEKGEI
L1-1A2_Sar                     ALQAYLRKEERARINNLTSQLKTLEKDQQKEPKPGRRKEIIKLRAEINDMETQKTIRKINETKSWFFEKINKIDKPLARLTKKERERTLISRIRNEKGDI
L1-1_ET                        SLNAYMKKEERRMTDTLTQNLQQLEQSQQNHPSNSKRKEIIKIRAELNQWEDKRTIQKINAAKSWFYERINKIDSPLAKLTKDRKEQTSIARMRDETGAI
L1-1_AMe                       AIQASLKRIEKSKMQFLYSHLKKLEQQQRDRPNPLTRKELTKIRAEINELETRTTVEQINRTRSWFFERIHKIDRPLAKLVQKQRERTEIIKIMTEKGEV
Meug                           SLNAYINKIEKEEINDLGLQLKKLEKEQIENPQVNTKLEILKTKGEINKIEIKKTIELINKTNSWFYEKTNKIDKPLVNLIKKKKEENQITNIKNERGEL
Fcat                           AIQAYLKKQEKSQIQNLTAHLKELEAEQQRQPKPSRRREIIKIRAEINNIESKKTVEQINETKSWFFEKINKIDKPLARLLKKKREMTQIDKIMNENGII
Mmul                           ALNAHKRKQERSKIDTLTSQLKELEKQEQTHSKASRRQEITKIRAELKEIETQKTLQKINESRSWFFEKINKIDRPLARLIKKKREKNQIDAIKNDKGDI
Pham                           ALNAHKRKQERSKIDTLTSQLKELEKQEQTHSKASRRQEITKIRAELKEIETQKTLQKINESRSWFFEKINKIDRPLARLIKKKREKNQIDAIKNDKGDI
```

```
                              ---------+---------+---------+---------+---------+---------+---------+---------+---------+---------+
                                      410       420       430       440       450       460       470       480       490       500
                              ---------+---------+---------+---------+---------+---------+---------+---------+---------+---------+
Conserved sites               XXXXXXIXXXXXXXXXXXLYXXXXXXXXXXXXXXXXXXXXXXLXXXXXXXXXNXPIXXXEXXXXIXXXXXXKXPGPDGXXXEFXXXXXXXXXPXLXXXXXXIX
lineage 1(L1-2_PVa)           TTDIAEIQRIIQEYYEKVYNTKFNNLEEMDQYLEKYNLPRLNQEELENLNRPISSMEIETIIKNLPKSKSPGPDGFTSEFYQTFKEDLIPILLKLFQKIE
lineage 2(L1-1_PVa)           TTDSTEIQWIIRKYYEQLYANKLDNLEEMDTFLETYNLPRLSQEETENLNRPITTNEIESVIKNLPKNKSPGPDGFIGEFYQTFKEELSPILLKLFQKIQ
L1-BT                         TTDNTEIQRIIRDYYQQLYANKMDNVEEMDKFLEKYNFPKLDQEEIENLNRPITTMEIETVIKNLSANKSPGPDGFTAEFYRKFREELTPILLKLFQKIA
L1-1_LA                       TTEPNEIKRIISDYYEKLYSNKFXNLEEMDEFLEKHYLPKLTHSEVEQLNRPITKKEIETVIKKLPTKKSPGPDGFTAEFYQTFREELTPLLLKVFQSIE
L1-1_Vpa                      TTNKIEIQNIIREYYEKLYGTKLDNLEEMDKFLETYCPPKLNQEETEHLNNPITRKEIEIAIKNLPTNKSPGPDGFTGEFYQTYKEELIPVLLKLFQTIE
L1-1_DN                       TTDPTEIKTIIRGYFEKLYSNKNDNLEEMDKFLETHKQPILTKEEIDDLNKPITSREIESVIKNLPTKKSPGPDGFTGEFYKTFRKELTPILLKLFQKIE
L1-1_Cpo                      TTDPIEIQKIINTYFENLYSQKFDNTEEIDRFLETYEVPKLDQEDVKLLNNPISVNEIENVIKSLPTKKSPGPDGFTAEFYKKYKEDLMPTLLKLFNEIE
L1-1_SSc                      TTDTTEIQRIIRDYYMQLYANKMENLEEMDKFLEKYNLPRLNQDEIEKMNGPITRTEIETVIKKLPTNKSPGPDGFTGEFYQTFREELTPLLLKLFQKIA
L1-1B_Cho                     TADPEEIXKIIRGYYEQLYANKLDNVEEMDNFLETYEQPRLTREEIEDLNQPITSKEIQSVIKKLPTNKCPGPDGFTGEFYQTFQKELTPILLKLFQNIE
L1-2_EC                       TTDSAEIQQIIREYYKKLYANRMDNLEEMDKFLDSYNLPKLTQEEADNLNRPITRKEIETAIKNIPKNKTPGPDGFPGEFYQTFREDLIPILFKLFQKIR
L1MAB2_ML                     TTDPTEIQMIVKQYYGQLYSNKLDNLEEMDKFLEKYNIPKLNQEESKNLNRPITMEEIEAVIRKLPANKSPGPDGFTGEFYQTFKEELKPTLLRLLQKIQ
L1-1_Str                      TTDTTEIQKIIRKYFETLYSNKIEDSEDIDKFLKAYDLPRLRQEDTHNLNRPITKEEIEEAIKRLPTKKSPGPDGYTAEFYKTFKEELIPILFKLFQEIE
L1-1_MD                       TSNEEEIKAIIRNYFAQLYGNKYTNLGEMDEYIQKYKLPRLTEEEIEFLNNPISEIEIHQAIKELPKKKSPGPDGFTCEFYQTFREQLTPILYKLFDIIS
L1-1_TS                       TSDPEEIQKIIRDYYVHLYGNKLENQKEMEDFLTSHNLPRLEQEEIETLNRPITIKEIDHVIRKLPTKKSPGPDGFPAEFYKTFKEELIPILLKVFQAIE
L1-1_OP                       TTDTASIKAIIRNYYKALYSNKSEDHQEMEKFLDFYHLPKLSPEATNDLNKPITEAEIESVIKDLPTKKSPGPDGFTTEFYKTFRTELTPILYKLFKTIE
L1_RN                         TTDSEEIQKIIRSYYKNLYSTKLENLQEMDNFLDRYQVSKLNQEQINQLNNPITPKEIEAVIKGLPTKKSPGPDGFSAEFYQTFIEDLIPILSKLFHKIE
L1A_Mim                       TTDPKEIQDTIYEYYKNLYAHKLENVEEMDKFLETHSLPRLNQEEIDSLNRPISTAEIETAIKNLPKKKSPGPDGFTPEFYHTYKEELVPILQKLFHNIE
L1-1_Cja                      TTDPTEIQTIIREYYKQLYAHKLVNLEEMDKFLDTCVLPSLNQEEVETMNRPITRSEVEAAIKSLPHKKSPGPDGFTAEFYQTHKEELLPFLLKLFQIIQ
L1-1_EE                       TTDTAEIQHIMRGFYEQLYATKLENLEEMNDFLDTYQLPKLSKEEVDNMNRPITANEIETVIKNLPKNKSPGPDGFTNEFYKTFKEELIPLLLKVFQKIE
L1-1_Tbel                     TTDIAEIHNIIRGYYERLYANKQDNLEEMDKFLDAYKLPRLDQEDIENLNRPITSQEIESVINNLPTKKSPGPDGFTCEFYKTFKEELTPILLKLFLKIQ
L1-1_Pca                      TTDATEIKNIITEYYERLYSNKFENLGEMDXFLDSYHLPKLSQREAEQLNKPITKEEIEKVIKTLPTKKSPGPDGFTAEFYQAFREELSPILLRLFHSIE
L1A_OC                        TTDTTEIKRIIRNYYKDLYASKQGNLSEMDRFLDTCNLPKLNQEDIENLNRPITETEIETVIKALPTKKSPGPDGFTAEFYQTFKEELTPILLKLFRTIE
L1HS                          TTDPTEIQTTIREYYKHLYANKLENLEEMDTFLDTYTLPRLNQEEVESLNRPITGSEIVAIINSLPTKKSPGPDGFTAEFYQRYKEELVPFLLKLFQSIE
L1-2_Dor                      TTETTKIQNIIREYFSKLYANKFENLTEMDLFLANIDMPKLNQDELNYLNRPISSIEIETAIRDLPAKKSPGPDGFTAEFYKAFKAELTPILLQLFNEIE
L1-Y_CF                       TTNTKEIQTILKTYYEQLYANKLGNLEEMDAFLESHKLPKLEQEEIENLNRPITREEIEAVIKNLPRHKSPGPDGFPGEFYQTFKEEIIPILLKLFGKIE
L1-1A2_Sar                    ATETNEIQKIIRDYFENLYATKQENLEEMDKFLDSYNLPRLNQEDLEXLNSPINIKEIETVIKSLPKNKSPGPDGFTSEFFQTFKEDLLPVLLKLFQEIE
L1-1_ET                       TTDPNEIKRIITKYYEGLYSNEFRNMEDMDKYLEKQSLPRLSQTEIKNLNKPIAKEEIERVIKNLPTKKAPGPDGFTAEFYQAFREELTPILHKVFHNIE
L1-1_AMe                      TTSTIEIARIIRNFYQQLYAKKLNNLEEMEAFLETYKLPRLKQEEIDFLNRPINYEEIESVINNLPNNKTPGPDGFPGEFYQTFKEEIIPILLKLFQKIE
Meug                          TSNEEEIKTIIRNYFAQLYAHKFDNLNEMDEYFKKYKLPRLTEEEVEYLNNPISEKEIEQAINELPRKKSPGPDGFTSEFYQTFKEQLIPILHTLFLKIG
Fcat                          TTNPSEIQTIIREYYEKLYANKLDNLEEMDKFLNTHTLPKLNQEEIESLNRPITSEEIESVIKNLPTNKSPGPDGFPGEFYQTFKAEIIPILLKLFQEIE
Mmul                          TTDPTEIQTTIREYYKHLYANKLENLEEMDNFLDTYTLPRLNQEEVESLNRPIAGSEIEAIINSLPTKKSPGPDGFTAEFYQRYKEELVPFLLKLFQSIE
Pham                          TTDPTEIQTTIREYYKHLYANKLENLEEMDNFLDTYTLPRLNQEEVESLNRPIXGSEIEAIINSLPTKKSPGPDGFTAEFYQRYKEELVPFLLKLFQSIE
```

```
                     ---------+---------+---------+---------+---------+---------+---------+---------+---------+---------+
                              510       520       530       540       550       560       570       580       590       600
                     ---------+---------+---------+---------+---------+---------+---------+---------+---------+---------+
Conserved sites      XXXXLPXXFXXXXXXLXPKXXXXXXXXXENXRPXSXMNXDAKXXXXXILAXRXQXXIXXXXHXDQXGFIXXXQXXXNXRKXIXXXXXIXXXXXXXXXXXSXD
lineage 1(L1-2_PVa)  EEAILPNSFYEANITLIPKPGKDNTKKENYRPISLMNTDAKILNKILANRIQQHIKKIIHHDQVGFIPGAQGWFNIRKSINVIHHINKLKNKNHMIISID
lineage 2(L1-1_PVa)  EEGRLPNSFYEATITLIPKPGKDITKKENYRPISLMNIDAKILNKILANRIQQYIKNIIHHDQVGFIPGMQGWXNIRKSINVIHHINKSKDKNHMIISID
L1-BT                EEGKLPNSFYEATITLIPKPDKDPTKKENYRPISLMNIDAKILNKILAIRIQQHIKKIIHHDQVGFIPGMQGFFNIRKSINVIHHINKLKNKNHMIISID
L1-1_LA              NDGILPNSFYEATISLIPKPGKDITKKENYRPISLMNIDAKILNKILANRIQQHIKKIIHPDQVGFIPGMQGWFNIRKTINVIHHINKTKDKNHMILSID
L1-1_Vpa             KEGILPNSFYEATITLIPKPGKDTTKKENYRPISLMNIDAKILTKILANRIQQHIKKIIHHDQVGFIPGTQGWFNIRKSINVIHHINKRKDKNHMIISID
L1-1_DN              TEGTLPNSFYDANITLVPKPNKDTTRKENYRPISLMNLDAKILNKILANRIQQHIKRIIHHDQVGFIPGMQGWFNIRKSINVIHHINRLKEKNHMIISID
L1-1_Cpo             REAILPKSFLEANITLVPKPEKDPTKKENYRPISLMNTDAKILNKILANRMQQIIKKIIHHDQVGFIPGMQGWFNIRKSINVIHHINKAKNKNHMIISID
L1-1_SSc             EEGILPNSFYEATVTLVPKPDKDSTKKENYRPXSLMNIDAKILNKILANRIQQYIKRIVHHDQVGFIPGMQGFFNIRKSISVIHHINKLKNKNHMILSID
L1-1B_Cho            ENGTLPNTFYEANINLIPKPGKDATKKENYRPISLMNIDAKILNKILANRIQRHIKKIIHHDQVGFIPGMQGWFNIRKSINVLQHINKSKEKNQMIISID
L1-2_EC              EDGTLPNTFYEANITLIPKPDKDTTKKENYRPISLMNIDAKILNKILATRIQQFIKRIIHQDQVGFIPGTQGWFNIRKSINVIHHINKLRNKNHMIISID
L1MAB2_ML            EEGTLPSSFYEASITLIPKPGKDITMKENYRPISLMNIDAKILNKILANRIQQYIRRIIHHDQVGFIPEMQGWYNIRKSINVIHHINKLKEKNHMVISID
L1-1_Str             KEGALPNSFYEANITLIPKPDKDTSKKENYRPISLMNLDAKILNKILANRIQKHIRKLVHHDQVGFIPGMQGWFNIRKSINAIHHINRLKDKNHMIISID
L1-1_MD              KEGVLPNSFYDTNMVLIPKPGRSKTEKENYRPISLMNIDAKILNRILAKRLQQVIRRIIHHDQVGFIPGMQGWFNIRKTIHIIDHINKQTSKNHMIISID
L1-1_TS              KDGTLPKSFYEANITLIPKPGKDPTKKENYRPISLMNIDAKILNKILANRIQQYISKIIHHDQVGFIPGMQGWFNIRKTINVIKYINRCQNKNHMIISLD
L1-1_OP              KEATLPNSFYEANITLIPKPDRELTEKENYRPISLMNIDAKILNKILANRIQKHIRQIVHPDQVGFIPGMQGWFNIRKSINVIHHIQKLKNKNHMIVSID
L1_RN                TDGALPNSFYEATITLIPKPHKDTTKKENFRPISLMNIDAKILNKILANRIQEHIKTIIHHDQVGFIPGMQGWFNIRKTINVIHYINKLKEQNHMIISLD
L1A_Mim              KNGNLPDTFYEANITLIPKPGKDATKKENYRPISLMNIDAKIFNKILANRIQTLIKKIIHHDQVGFIPGMQGWFNIRKSINAIHHINRSKNKDHMILSID
L1-1_Cja             KEGILPKSFYETNIILIPKPGRDSTRKENFRPISMMNIDAKIFNKILASRLQQHIKKLIHHDQVGFIPGMQGWFNIRKSINVIHHINRTKNKNHMIISID
L1-1_EE              DTGILPASFYEANITLIPKADRDTTKKENYRPISLMNIDAKILNKILANRIQQYIKKIVHHDQVGFIPGMQGWFNIRKSINVIHHINKSKTKNHMVISID
L1-1_Tbel            KEGNLPNSFYEASIILIPKTDKDMTKKENYRPISLMNIDAKILNKILANRIQQHITKIIHHDQVGFIPGMQGWFNIRKTINVIHHINRKKDKNHMIISID
L1-1_Pca             RDGVLPNSFYEASITLIPKPGKDPTKKENYRPISLMNLDAKIFNKILANRIQQHIKKIIHHDQVGFIPGMQGWFNIRKTINVINHINNTKDKDHLILSID
L1A_OC               EEGILPNSFYEASITLIPKPEKDAALKENYRPISLMNIDAKILNKILANRMQQHIRKIIHPDQVGFIPGMQGWFNVRKTINVIHHINRLQKKNHMIISID
L1HS                 KEGILPNSFYEASIILIPKPGRDTTKKENFRPISLMNIDAKILNKILANRIQQHIKKLIHHDQVGFIPGMQGWFNIRKSINVIQHINRAKDKNHMIISID
L1-2_Dor             RESSLPNTFYEAIITLIPKPGRDSSKKENYRPISLMNIDAKLLNKILANRLQQFIQKIIHHDQTGFISGMQSWFNIRKSINIIHHINRSKNKNHMVISID
L1-Y_CF              RDGVLPNSFYEASITLIPKPDKDPAKKENYRPISLMNMDAKILNKILANRIQQYIKKIIHHDQVGFIPGTQGWFNTRKTINVIHHISKRKTKNHMILSLD
L1-1A2_Sar           ETETLPNSFYEAHISLIPKANKDTTKKENYRPISLMNTDAKILNKILANRIQQLIKKIIHHDQVGFIPGMQGWFNIRKSINIIHHINKRKDKNHIIISID
L1-1_ET              EDGKLPNSFYEASITLIPKQGKDPTGIENYRPISLMNIDAKILNKILANRIQKYIKHIIHHDQVGFIPGMQGWFNIRKSINIIHHIEKKKDKNHMIISID
L1-1_AMe             TEGKLPNSFYEANITLIPKPGKDPLKKENYRPISLMNMDAKILNKILANRIQQYIKRIIHHDQVGFIPGMQAWFNTRKSINVIHHINKKRLKNHMILSID
Meug                 EEGVLPNSFYDTNMVLIPKPGRDKTEKENYRPISLMNIDAKILNKILAKRIQHLITRLIHYDQVGFIPGLQGWFNIRKTISIIDHINNKANKNHMIISID
Fcat                 REGKLPDSFYEASITLIPKPDRDPVKKENYRPISLMNMDAKILNKILANRIQRHIKRIIHHDQVGFIPGMQGWFNIRKSINVIHHINKKKEKNHMILSID
Mmul                 KEGILPNSFYEANIILIPKPGRDTTKKENFRPISLMNIDAKILNKILANRIQQHIKKLIHHDQVGFIPGMQGWFNIRKSINIIQHINRTKDKNHMIISID
Pham                 KEGILPNSFYEANIILIPKPGRDTTKKENFRPISLMNIDAKILNKILANRIQQHIKKLIHHDQVGFIPGMQGWFNIRKSINIIQHINRTKDKNHMIISID
```

```
                    ---------+---------+---------+---------+---------+---------+---------+---------+---------+---------+
                             610       620       630       640       650       660       670       680       690       700
                    ---------+---------+---------+---------+---------+---------+---------+---------+---------+---------+
Conserved sites     AEKAFDXXQXXFXXXXLXXXGXXGXXXXXXXXXXYXXPXAXIXXNGXXXXXXXXXXGXXQGCPLSPXLFXXVLEXLAXXXRXXXXIXGXXXXXXEXKXSLX
lineage 1(L1-2_PVa) AEKAFDKIQHPFMIKTLNKIGIEGKYLNIIKAIYDKPSANIILNGEKLKTFPLRSGTRQGCPLSPLLFNITLEVLASAIRQEKDIKGIQIGNEEVKLSLF
lineage 2(L1-1_PVa) AEKAFDKIQHPFMIKTLSKVGIEGTYLNIIKAIYDKPTANIILNGERLKAFSLRSGTRQGCPLSPLLFNIVLEVLATAIRQEREIKGIRIGKEEVKLSLF
L1-BT               AEKAFDKIQHPFMIKTLQKAGIEGTYLNIIKAIYDKPTANIILNGEKLKAFPLKSGTRQGCPLSPLLFNIVLEVLATAIRAEKEIKGIQIGKEEVKLSLF
L1-1_LA             AEKAFDKVQHPFMIKTLTKIGIEGKFLNIIKGIYAKPTANITLNGENLKAFPLRTGTRQGCPLSPLLFNIVLEVLARAIRLDKEIKGIRIGKEEVKLSLF
L1-1_Vpa            AEKAFDKIQHPFMIKTLAKVGIEGTYLNIIKAIYDKPTASIVLNGEKLKSFPLKSGTRQGCPLSPLLFNIVLEVLATAVRQEKEIKGIQIGKEEVKVSLY
L1-1_DN             AEKAFDKIQHPFLIKTLQKIGIQGNFLNMIKSIYEKPKANIVYNGEILKSFPLNSGTRQGCPLSPLLFNIVLEVLARALRQEPDIKGIQIGKEEVKISLF
L1-1_Cpo            AEKAFDKVQHLFMIRTLQKIGIDGLYLNIIKAIYDKPTASIILNGQKLKAFTLKSGTRQGCPLSPLLFNIVLEVLARAIRQEREIKGVKIGKEEVKLSLF
L1-1_SSc            AEKAFDKIQHPFLIKTLQKVGITGTYLNMIKAIYDKPTANIILNGEKLKEFPLRSGTRQGCPLSPLLFNIVLEVLATAIREVKEIKGIQIGKEEVKLSLF
L1-1B_Cho           AEKAFDKIQHPFLIKTLQKVGIEGNFLNMIKSIYEKPTASIVLNGERLKAFPLRSGMRQGCPLSPLLFNIVLEVLARAIRQDKEIKGIQIGKEEVKLSLF
L1-2_EC             AEKAFDKIQQPFMIKTLNKMGIEGNYLNIIKAIYDKPIANIILNGQKLNPIPLKTGTRQGCPLSPLLFNIVLEVLARAIRQEKGIKGIQIGREEVKLSLF
L1MAB2_ML           AEKAFDKIQHPFLIKTLRKVGIEGSYLNIIKAIYDRPTANIILNGQKLTPFPLRTGTRQGCPLSPLLFNIVLEVLAIAIRQEEEIKGIQIGKEEVKLSLF
L1-1_Str            AEKAFDKVQHPFMFKTLEKLGITGTYLNIVKAIYAKPQASIILNGEKLKAFPLKSGTRQGCPLSPLLFNLVLEVLARAIRQTKEIKGIKIGKEEIKLSLF
L1-1_MD             AEKAFDKIQHPFLLKTLESIGIEGSFLKIINSIYLKPTANIICNGDKLDAFPIRSGVKQGCPLSPLLFDIVLETLAVAIREDKEIEGIRIGKEETKLSLF
L1-1_TS             AEKAFDKIQHPFLIKTLEHLGIRGTYLKIVKAIYEKPTASILLNGQKLEPIPLKTGTRQGCPLSPLLFNIVLEVLARAIREEEAIRGIQIGKEEVKLSLY
L1-1_OP             AEKAFDKIQHHFLLKTLTKVGIDGKIHNIIKAIYEKPNASIILNGEKLEPFPLRSGTRQGCPLSPLLFNIVLEVLAEAIRQEKEIRGIQMGNEEVKLSLY
L1_RN               AEKAFDKIQHPFMIKVLERIGIQGPYLNIVKAIYSKPVANIKLNGEKLEAIPLKSGTRQGCPLSPYLFNIVLEVLARAIRQQKEIKGIQIGKEEVKISLF
L1A_Mim             AEKAFDKIQHPFMIRTLKKIGIEGTYLKMIQAIYDRPIANIILNGERLKSFPLRTGTRQGCPLSPLLFNIVLEVLATAIRQENGIKGIQIGAEEIKLSLF
L1-1_Cja            AEKAFDKIQQPFMLKTLNKLGIDGTYLKIIKAIYDKPTANIILNGQKLEAFPLKSGTRQGCPLSPLLFNIVLEVLARAIRQEKEIKGIQIGKEEAKLSLF
L1-1_EE             AEKAFDKIQHPFMIKTLQKMGIDGKFLKIVESIYSKPTANIILNGEKLEAFPLRSGTRQGCPLSPLLFNIVLEVLAIAIRQEQGIKGIQIGREEVKLSLF
L1-1_Tbel           AEKAFDKIQHPFMIKTLSKIGIEGSFLNTIKAIYEKPTANIILNGEKLKAFPLKTGTRQGCPLSPLLFNIVLEVLAIAIREENSIKGIRIGKEELKISLF
L1-1_Pca            AEKAFDKVQHPFMLKTLSKMGIDGKFLNTIRAIYTKPIAHIILNGERLKDFPLRSGTRQGCPLSPLLFNIVLEVLARAIRQDQEIKGICIGKEEVKISLF
L1A_OC              AEKAFDKIQHPFMMKTLSKLGLEGTFLNTIKAIYEKPTANILLNGEKLEAFPLRSGTRQGCPLSPLLFNIVLEVLARAIRQEKEIKGIQIGKEELKLSLF
L1HS                AEKAFDKIQQPFMLKTLNKLGIDGTYFKIIRAIYDKPTANIILNGQKLEAFPLKTGTRQGCPLSPLLFNIVLEVLARAIRQEKEIKGIQLGKEEVKLSLF
L1-2_Dor            AEKAFDKIQHPFMMKVLEKLGIHGTFLNTIKAVYDKPTASIILNGEKLKPFPLNSGTRQGCPLSPLLFNLVLECLATAIRQDADIKGIQIGKEEIKLSLF
L1-Y_CF             AEKAFDKIQHPFLIKTLQSVGIEGTFLDILKAIYEKPTANIILNGEALGAFPLRSGTRQGCPLSPLLFNIVLEVLASAIRQQKDIKGIQIGKEEVKLSLF
L1-1A2_Sar          AEKAFDKIQHPFMMKTLAKMGIEGTFLNIVKAIYHKPTASIVLNGEKLRAFPLRTGTRQGCPLSPLLFNIVLEVLAIAIRQEKDIKGIQVGKEEIKLSLF
L1-1_ET             AEKAFDNIQHPFLIKTLMKIGLEGKFLKLIQAIYERPTANIIVNGEKTKTIPLKKGTRQGCPLSPLLFNIVLEVLANNIRQRKDIKGIQMGEEEVKLSLF
L1-1_AMe            AEKAFDKIQHPFLIKTLQSVGIEGTFLNLIKAIYEKPTASIILNGEKLEAFPLRSGTRQGCPLSPLLFNIVLEVLATAIRQQKGIKGIQIGKEEVKLSLF
Meug                AEKAFDKIQHPFLLKTLENVGIKGTFHKIISSIYLKPSASIICNGDKLDAFPIRSGVKQGCPLSPLLFNMVLEMLAVAIRQDKDIQGIRIAKEETKLSLF
Fcat                AEKAFDKIQHPFLIKTLEKVGIEGTYLKIIKAIYEKPTANIILNGEKLRAFSLRSGTRQGCPLSPLLFNIVLEVLASAIRQQKEIKGIKIGKDEVKLSLF
Mmul                AEKAFDKIQQPFMLKTLNKFGIDGTYLKIIRAIYDKPTANIILNGQKLEKFPLKTGTRQGCPLSPLLFNIVLEVLARAIRQEKEIKGIQLGKEEVKLSLF
Pham                AEKAFDKIQQPFMLKTLNKFGIDGTYLKIIRAIYDKPTANIILNGQKLEKFPLKTGTRQGCPLSPLLFNIVLEVLARAIRQEKEIRGIQLGKEEVKLSLF
```

```
                             ---------+---------+---------+---------+---------+---------+---------+---------+---------+---------+
                                      710       720       730       740       750       760       770       780       790       800
                             ---------+---------+---------+---------+---------+---------+---------+---------+---------+---------+
Conserved sites              ADDMXXYXXXPXXSXXXXXXXXXXXXXXVXGYXINXXKXXXXXXXXXXXXXXXXXXXXXXXFXXXXXXXXXYLGXXLXXXXXXLXXXNXXXXXXXXXXXXXXXWX
lineage 1(L1-2_PVa)          ADDMILYIENPKDSTRTLLETISKYSKVSGYKINVQKSIAFLYSNNEVSEKEVEKIIPFAIATKRIKYLGINLTKHVKDLYNENYKTLLKEIEEDTKKWK
lineage 2(L1-1_PVa)          ADDMILYIENPKDSTKKLLELINEFSKVAGYKINIQKSVAFLYTNDELSEREIKKTIPFTIASKTIKYLGIKLTKKVEDLFSENYKSLKKEIEEDTNKWK
L1-BT                        ADDMILYIENPKDSTRKLLEIINDYSKVAGYKINTQKSLAFLYTNNEKTEREIKETIPFTIATERIKYLGIYLPKETKDLYLENYKTLVKEIKEDTNRWR
L1-1_LA                      ADDMIXYTENPKESSRKLLKLIEEFGRVSGYKINIQKSLGFLYINKKNTEEEITKSIPFTVAPKKIRYLGINLTKDVKDLYKENYKALLQEIQKDILKWK
L1-1_Vpa                     ADDMLLYIENPKRSTQKLLELIEEFSKVAGYKINVQKSVAFLYTNDKSTEKESKETIPFKIAPKVIKYLGINLTKEAKELYTENYKPLMKEIKEDFKKWK
L1-1_DN                      ADDMILYIENPERSTTKLLELINEFSKVAGYKINAQKSVAFLYTNNEQDQEEIKKQIPFTIVNKKIKYLGINLTKEVKNLYTENYTRLFKEIKEDLNKWK
L1-1_Cpo                     ADDMILYIEDPLNSIERLLDTINKFSNVAGYKINTQKSIAFLYTNNKITEREIRETALFTLASKKMKYLGITLTKEVKDLYSENYNTLKKEIEDDLRKWK
L1-1_SSc                     ADDMILYLENPKDSTRKLLELIHEFGKVAGYKINTQKSIAFLYTNNEKAEKEIREAIPFTIASKRIKYLGVNLPKETKDLYSENYKPLMKEIKDDTNRWK
L1-1B_Cho                    ADDMILYLENPEKSMIQLLELINKFSKVAGYKINAHKSVMFLYARNERSEETLKKKIPFSIATKKIKYLGINLTKDVKDLYKENYITLLKEIERDLKRWK
L1-2_EC                      ADDMILYIENPKESIGKLLEVINNYSKVAGYKINLHKSVAFLYSSNEPTEKELKNTIPFTIATKRIKYLGVNLTKEVKDLYNENYKAFLRELDDDIRRWK
L1MAB2_ML                    ADDMILYIQNPRDSIKKLLDLIHEFGNVAGYKINPKKSEAFLYTNSELSEREIRKTIPFTIAPKKLRYLGINLTKEVKDLYSENYRTLKKEIEEDINRWK
L1-1_Str                     ADDMIIYLTDPKGSTKKLLELINEFSKVAGYKINTHKSKAFLYISNKTSEMEIRKTTPFTISSKKIRYLGINLTKEVKDLYNENYRTLKREIEEDLRRWK
L1-1_MD                      ADDMMVYLKNPRDSTKKLIEIINNFSKVAGYKINPHKSSAFLYISNTAQQQELEREIPFKITLDKIKYLGIYLPRQTQELYEHNYKTLATQLKLDLNNWK
L1-1_TS                      ADDMXVYLENPRESVKGLLTLIKAFGKVSGYKINVQKTIAFLYTNNKQTETQIKNTVPFTIATKKMKYLGIFLTRDVKDLYNENYKTLLKEIKADTNKWK
L1-1_OP                      ADDMILYVEEPRDSIQRLLELVREFGRVAGYKINEQKSTAIVYANSPKMEKDLTSKIPFKITEKSMKYLGINLTKNVGDLFEENYKLLKKEIEQDLKRWS
L1_RN                        ADDMIVYLSDPKSSTRELLKLINNFSKVAGYKINSNKSVAFLYTKEKQAEKEIRETTPFIIDPNNIKYLGVTLTKQVKDLYNKNFKTLRKEIEEDLRRWK
L1A_Mim                      ADDMILYLENPKDSTKKLLELINEFSKVSGYKINTQKSEAFIYANNNLIENQIKDSIPFTIATKKLKYLGIYLTKEVKDLYRENYETLRKEIAEDVNRWK
L1-1_Cja                     ADDMIVYLEDPIVSAQNLLKLISNFSKVSGYKINVQKSQAFLYTNNRLKESQIKNELPFTIATKRIKYLGIQLTRNVKDLFKENYKPLLNEIREDTNRWR
L1-1_EE                      ADDMIVYMEKPKESSKKLLEIIRQYSNVSGYKINIQKSVAFLYANTKLEEIEIQKSVPFSIATKTIKYLGVNLTKEVKDLYTENYESLLKEIEKDTKKWK
L1-1_Tbel                    ADDMMVYLEDPKVSMKKLLEVISEYSKVAGYKINIQKSIAFLFMNNKLNEAELKKGIPFTVATRCIRYLGINLTKKVKDLYKENYENLKKDIESDIKKWK
L1-1_Pca                     ADDMIVYAENPKESISKLLKLIEEFSKVSGYKINISKSVGFLYTNNETVKEEIIKSIPFTIAPKKIKYLGINLTRETTDLYKENYKPLLQETKRDLDKWK
L1A_OC                       ADDMILYLGDPKNSTKRLLELIEEFGKVAGYKINAQKSTAFVYTDNAMAEEELLRSIPFTIATKTIKYLGINLTKDVKDLYDENYKTLKKEIEEDTKKWK
L1HS                         ADDMIVYLENPIVSAQNLLKLISNFSKVSGYKINVQKSQAFLYTNNRQTESQIMGELPFTIASKRIKYLGIQLTRDVKDLFKENYKPLLKEIKEDTNKWK
L1-2_Dor                     ADDMILYLKNPIDSTPKLLKLIQNFGKVAGYKINPQKSMAFLYANNEKSVAEIRKATPFVIAPQKIKYLGINLTKEVKDLYDENFKILKKEINTELKKWQ
L1-Y_CF                      ADDMILYIENPKVSTPRLLELIQQFGSVAGYKINAQKSVAFLYTNNETEEREIKESIPFTIAPKSIRYLGINLTKDVKDLYPQNYRTLLKEIEEDTKRWK
L1-1A2_Sar                   ADDMILYLENPKTSTKKLLETIDSYSKVAGYRINTQKSMAFLYANNEREESDMXKAIPFTIAPQKIKYLGISLTKEVKDLYNENYKTLLQEIKEDTRKWK
L1-1_ET                      ADDMILYIENPKSSTAGVLTAIEEYGRVAGYKINKQKSVGFLYTSDRTMEEGIKKEVPFTVAKNKLKYLGIYLTKNTKDLYKENYKTLLQETKSDLHKWK
L1-1_AMe                     ADDMILYMENPKESTPKLLEVIEQFSKVAGYKINAQKSVAFLYTNNETEEREIRESIPFTITPKTMRYLGINLTRDVKDLYARNYRSLLKDIEEDIKRWK
Meug                         ADDMMIYLENPRDSSKKLLELINNFGKVAGYKINPHKSSAFLYISNKVQQQEIEREIPFKVRVDSIKYLGVYLPKQTQGLYEHNYKTLFAQIKSDLSKWK
Fcat                         ADDMILYMENPIDSTKSLLELIQEFSKVAGYKINVQKSVAFLYTNNEATERQIKKLIPFTIAPRSIKYLGINLTKDVKDLYAENYRKLMKEIEEDLKKWK
Mmul                         ADDMIVYLENPIVSAQNLLKLISNFSKVSGYKINVQKSQAFLYTSNRQTESQIRNELPFTIASKRIKYLGIQLTRDVKDLFKENYKPLLSEIKEDTNKWK
Pham                         ADDMIVYLENPIVSAQNLLKLISNFSKVSGYKINVQKSQAFLYTSNRQTESQIMNELPFTIASKRIKYLGIQLTRDVKDLFKENYKPLLSEIKEDTNKWK
```

```
                    ---------+---------+---------+---------+---------+---------+---------+---------+---------+---------+
                            810       820       830       840       850       860       870       880       890       900
                    ---------+---------+---------+---------+---------+---------+---------+---------+---------+---------+
Conserved sites     XXXCXWXGXXNXXXKMXXLPXXXYXXXAXPIXXXXXXXXXXXXXXXXFXWXXXXXRIXXXXXXXXXXXGGXXXPXXXXYYXAXXXKXXWYWXXXRXXDXWX
lineage 1(L1-2_PVa) DIPCSWIGRINIVKMTILPKALYRFNAIPIKIPSAFFKEIEQKIIRFVWKHKRPRIAKAILRKKNEAGGITLPDFKLYYKATVIKTAWYWQQNRHTDQWN
lineage 2(L1-1_PVa) HIPCSWVGRINIVKMAILPKAIYRFNAIPIKIPMAFFRELEQIILKFIWSHKKPRIATAILRKKNKVGGITLPDIKLYYKATVIKTAWYWHKNRYIDQWN
L1-BT               NIPCSWIGRINIVKMSILPKAIYRFNAIPIKLPTVFFTELEQIISQFIWKYKKPRIAKAILRKKNGAGGINLPDFRLYYRATVIKTVWYWHKDRNMDQWN
L1-1_LA             NIPCSWIGRLNIVKMSILPKAIYTXNALPIQIPMSYFKGIEKQITNFIWKGKKPRISKALLKKKKKVGGLTLPDFRTYYTATVVKTAWYWYNNRHIDQWN
L1-1_Vpa            DIPCSWIGRINIVKMVTLPKAIYGFNAIPIQLPRTYFTELEQIIIKFIWNHQRPRIAKALLKRKKEAGGITLPDFRQYYRATVIKTAWYWYQNRHIDQWN
L1-1_DN             NIPCSWIGRLNIIKMSILPKLIYTFNAIPIKINAAFFKELEKLTMKFIWKGKRPRIAKDILKKKNEIGGITLPDFKTYYKATVVKTAWYWHKERHTDQWN
L1-1_Cpo            DIPCSWIGRTNIVKMAILPKLLYRFNAIPIKIPSTYLIDLEKSLLNFIWNQKRPRIAKAILSSKDKAGGITIPDLKLYYKATVVKSTWYWNQNRAEDQWN
L1-1_SSc            DIPCSWIGRVNIIKMTILPKAIYRFNAIPIKLPRTFFTELEQNILKFVWKHKRPRIAKDILKKKNGAGGIRLPDFRLYYKATVVKTAWYWHKDRHIDQWN
L1-1B_Cho           NIPCSWIGRLNVIKMSILPKLIYRFNAIPIKIPTTYFADLEKLVIKFIWKGKMPRIAKNTLKKKNEVGGLTLPDFEAYYKATVVKTAWYWHKDRYIDQWN
L1-2_EC             DIPCTWIGRINIVKMSILPKAIYRFNAIPIRIPMTFFTELEQRILKFIWGNKRPRIAKAILRKKNKTGGITIPDFKTYYKATVIKTAWYWYKNRCTDQWN
L1MAB2_ML           NIPCSWIGRINIIKMSILPKAIYKFNALPIKIPMAFFKDLERTLQKFIWNKKRPRIAAAILRK-NKVGGISMPDIKLYYKATVLKTAWYWHKNRHIDQWN
L1-1_Str            NVPCSWIGRTNIIKMAILPKVLYRFNAMPIRIPXAFLVEIDKAIMKFIWKNKRPRIAKAILSRKCESGGIAIPELKLYYKAIVTKTAWYWYQNRRVDQWY
L1-1_MD             NINCSWIGRANIIKMTILPKLIYLFSAIPIELPKYFFTDLEKTITKFIWKNKRSRISREIMKKNTYDGGLAVPDLKLYYKAAVIKTIWYWLRNRKEDQWN
L1-1_TS             NIPCSWIGRINIVKMSILPKAIYKFNAIPIKLPTTFFSDLEKTTQEFIWKHKRPRIARTILSKKNKAGGITIPDFKLYYKATIIKTAWYWYRNRHIDQWN
L1-1_OP             NIPCSWIGKINIIKMSILPKAIYTFNAIPIKLPKTFFTELETMIQRFIWKHKKPRIARTILKNRKLAGGITVPDLWTYYRAVVIKTAWYWHKDREEDQWS
L1_RN               DLPCSWIGRINIVKMAILPKAIYRFNAIPIKIPIQFFKELDRTICKFIWNNKKPRIAKAILNNKRTSGGITIPELKQYYRAIVIKTAWYWYRDRQIDQWN
L1A_Mim             SIPCSWIGRLNIIKMSILPKLIYRFNAIPIKIPSAFFTDIEKIILRFVWNQRRPRISRAILGNKNKMGGINMPDIKLYYKAVVIKTIWYWHKNRNIDQWN
L1-1_Cja            NIPCSWLGRINIVKMAILPKVIYRFNAIPIKLPMTFFTELEKTTLNFIWNQKRARIAKSILSKKNTAGGITLPDFKLYYKATVIKTAWYWYQNRDIDQWN
L1-1_EE             DIPCSWVGRINIIKMNILPRAIYKFNAIPIKIPSTFFRRIEKMLQMFIWNQKRPRIAKTILRKKNRTGGITLPDLKLYYRAIVIKTAWYWNMNRHTDQWN
L1-1_Tbel           DIPCSWIGRINIIKMAILPKLIYMFNAIPIKIPEIFFRDLEKTIIEFVWNHKRPRIAKAILRKKNGVGGISLPDPKIYYKAVVIKTAWYWHKNRHVDQWK
L1-1_Pca            NLPCSWIGRLNIVKMSILPKAIYLYNAIPIQVPASFFNEMEKQITNFVWKGKKPRISKVLLKKKNTLGGLSLPDLRTYYTATVVKTAWYWYKNRQIDQWN
L1A_OC              NLPCSWIGRINIIKMSILPKAIYRFNAIPIKIPKTFFSDLEKMVLKFIWRHKRPRIAKAILYNKNKAGGITIPDFRTYYRAVVIKTAWYWYRNRWIDQWN
L1HS                NIPCSWVGRINIVKMAILPKVIYRFNAIPIKLPMTFFTELEKTTLKFIWNQKRARIAKSILSQKNKAGGITLPDFKLYYKATVTKTAWYWYQNRDIDQWN
L1-2_Dor            SLPCSWIGKINIVKMAILPKAIYKFNAIPIKIPTSFFKEIEEAIQKFIWNNKRPRIAKTLLNGKNTAGGISIPNFKLYYKAIVIKTAWYWHKNRPQDQWN
L1-Y_CF             NIPCSWIGRINIVKMSMLPRAIYTFNAIPIKIPWTFFRELEQIILRFVWNQKRPRIARGILKKKTISGGITMPDFRLYYKAVVIKTVWYWHKNRHIDQWN
L1-1A2_Sar          DIPCSWIGRINIVKMAILPKALYKFNAIPIGIPLXFFKEMEQALLKFIWNNKPPRIAKAILGKK-KMGGINLPNFQLYYKAVVIKTAWYWNKGRAADQWN
L1-1_ET             NIPCSWIGRLNIVKMTILPKALYKFNAIPIQIPSTFFKELEKLTTNFIWRGKKPRISRELLKKKDTVGGLALPDFNAYYTATVVKTAWYWHNDRHSDQWK
L1-1_AMe            NIPCSWIGRINIVKMSILPRAIYTFNAIPIKIPRTFFKELEQIVLKFVWNQKRPRISKELLKRKNKAGGITMPDFELYYKAVITKTAWYWHKNRHIDQWN
Meug                NISCSWVGRANIIKMTILPKLIYLFSAIPIKLSDNYFLELDKIISKFIWKNKRSRISKGLMKRNAWEGGLALPDLKLYYKAAIIKTTWYWLRNREVDKWN
Fcat                DIPCSWIGKINIVKMSILPKAIYTFNAIPIKIAPAFFSKLEQAILKFIWNHKRPRIAKGILKKKTKAGGITIPDFSLYYKAVIIKTAWYWHKNRHIDQWN
Mmul                NIPCSWIGRINIVKMAILPKVIYRFNAIPIKLPMSFFTELEKTALKFIWNQKRARISKTILSQKNKAGGITLPDFKLYYKATVTKTAWYWYQNRDIDQWN
Pham                NIPCSWIGRINIVKMAILPKVIYRFNAIPIKLPMSFFTELEKTALKFIWNQKRARISKTILSQKNKAGGITLPDFKLYYKATVTKTAWYWYQNRDIDQWN
```

```
                              ---------+---------+---------+---------+---------+---------+---------+---------+---------+---------+
                                      910       920       930       940       950       960       970       980       990      1000
                              ---------+---------+---------+---------+---------+---------+---------+---------+---------+---------+
Conserved sites               XXXXXXXXXXXXXXXXDXXXXXXXXXXXXXFXKXXXXXXWXXXXXXXXXXXXXXXPXTXXXSXWXXXLXXXXXXXXXXXXXXXXXXXXXXXXXXXXXXXX
lineage 1(L1-2_PVa)           RIESPEVNPYLYGQIIFDKKAKNIQWRKESLFNKWCWENWKATCKRMKLDCCLSPHTKINSKWIKDLNIRPETIKCIEENIGTKLKDLGLKEDFMN-LTS
lineage 2(L1-1_PVa)           RIESPEINPRLYAHLIYDKGGKNIQWGKDSLFNKWCWENWTDTCKKKKLDHLLTPYTRIDSKWIKDLNVRPETIKLLEENIGSKLSDITNRNFFSD-ISP
L1-BT                         KIESPEINPRTYGHLIFDKGGKDIQWIKDNLFNKWCWEIWSTTCKRMKLDHFLTPYTKINSKWIKDLNVRPETIKLLEENIGKTLSDIYHSRILYD-PPP
L1-1_LA                       RIENPDINPSTYEQLIFDKGPVSVNWGKDSLFNKWCWHNWISICKKMKQDPYLTPCTKTNSKWIKDLNIKTKTIKIMEEKIGTTLGALIQGINRIQNITK
L1-1_Vpa                      RIESPEMNPQTFGQLIFDKGGKNIQWNKDSLFSKWCWENWTAACKTMKLEHTLTPYTKINSKWIKDLNIRQDTINLLEENIGKTLSDIHFKNFLLE----
L1-1_DN                       RIESSDIEPHIYSHIIFDKATKPSQLGESGLFNKWCLENWIAICRRMKEDYHLTPYTKINSRWIKDLNIRAKTIKTLESSVGKHLQDLVIG-NGFMNITP
L1-1_Cpo                      RLEDTTTTTNTLNHLIFDKGAKQVHWKNDSLFNKWCWKNWLSICRKLKLDPCLSPCTKLKSKWVKDLNIKTETLNLLEDKLGRNLEDIGVGREFMN-RTQ
L1-1_SSc                      RIESPELNPRTYSQLIYDKGGKNIQWRKDSLFNKWCWENWTATWKRMKLEHSLTPYTKINSKWIKDLDIRPDTIKLLEENIGQTLSDINDSNIFSD-PPI
L1-1B_Cho                     RIENSEIDPQIYGRLIFDKAPKATELGHNSLFNKWGWESWISISKRMKEDPYLTPYTKINSKWIKDLNIKDSTIKLLEDNVGRHLQDLVLG-GHFLDLTP
L1-2_EC                       RIESPEIKPHIYGQLIFDKGAEGIQWRKESLFNKWCWENWKATCKRMKIDHSFSPFTKINSKWIKDLKVRPETIRLLEENVGSTLFDISIKRIFSDTMPS
L1MAB2_ML                     RIESPEISPNQYAQLIFDKGGMNIQWSQDSLFNKWCWENWTDICKKMKLDHQLTPYTRINSKWIKDLNVRQETIKILEEYKGNKISDICQNNFFTD-TAP
L1-1_Str                      RIEDTETNPQSYNYLIFDKGAKNMQWRKDSIFNKWCWENWKSICNKMKLNPFLSPATKVNSKWIKELDIKXETLRLIEGKVGYDLHIVGSGSKFLN-RTP
L1-1_MD                       RLGEND-----LSKTVYDKPKDPSFWDKNPLFDKNCWENWKTVWERLGIDQHLTPYTKINSKWVSDLNIKKETISKLGKHRIVYMSDLWEGKGFKT-KQD
L1-1_TS                       RIEIPEAKPQFLNQLIFDKAPTTYHWGEENLFSKWCWENWLTTCRRLKQDPYLSPCTKVNSKWIRDLNVKPQTIRTLEK-EGNTLMEIGTGIQFLY-KTR
L1-1_OP                       RIETPEGNPHRYSQIIFDKKTNDNPGKWEGLFNKCCWDNWLIACRNKKIDPHLSPYTKIRSKWITDLNLHPETFKLLEENVGNTLQHLGVGPHFLK-KTP
L1_RN                         RIEDPEMNPHTYGHLIFDKGAKTIQWKKDSIFSKWCWFNWRATCRRMQIDPCLSPCTKLKSKWIKDLHIKPDTLKLIEEKLGKHLEHMGTGKNFLN-KTP
L1A_Mim                       RCENPDIKPSSYSHLIFDKADKNIRWGKESLFNKWCWENWIATCRRLKQDPHLSPLTKTNSRWITDLNLRYETIRTLEEKVGNTLLDIGLGKEFMK-KSP
L1-1_Cja                      RTEASEATQHIYNHTIFDKPDKNKQWGKDSLFNKWCWENWLAMCRKQKLDPFLTPYTKINSRWIKDLNIRPNTIKTLEENLGKTIQDIGVGKDFMT-KTP
L1-1_EE                       RIESPEMRPHTYGHLIFDKGAQTITWGKQSLFNKWCWKQWVETCRRMKLNHCISPNTKVNSKWIKDLDVRPETIRYLEENIGRTFFRINFKDIFNE-TNP
L1-1_Tbel                     RIETPEITPKAYSQLIFDKGYQSIHWEKENLFSKWCWKNWVSTCRKMKLNPHLSPLTTVNSKWIKDLNLRPQTIKLLVENAGETLGDIGVGKDFLN-KTP
L1-1_Pca                      RIETPEMTPSAYEQLIFDKGPKTLNWGKNSIFNKWCWQNWLSICNKLNQDPYLTPYTKTNSNWIKDLNIKPKTIKLIQEKVGTTMEVLIHNEDRLQNLSK
L1A_OC                        RIETPEINPNIYSQLIFDQGSKTNSWSKDSLFNKWCWENWISTCRIMKQDPYLTPYTKIHSTWIKDLNLRPDTIKLLEN-IGETLQDIGTGKXFLE-KTR
L1HS                          RTEPSEIMPHIYNYLIFDKPEKNKQWGKDSLFNKWCWENWLAICRKLKLDPFLTPYTKINSRWIKDLNVRPKTIKTLEENLGITIQDIGVGKDFMS-KTP
L1-2_Dor                      RIENPEMNLQTYSHLIFDKGAKTIEWKKDSLFSKWCWQNWLTTCRKLKLDPYISPCTRINSKWIKDLEVKADTLKTLQDGIGETLGLLGSGRNFLN-RDP
L1-Y_CF                       RIENPEVDPELYGQLIFDKGGKTIHWKKDSLFNKWCWENWTSTCRRMKLDHSLSPYTKINSKWMKDLNVRQDSIKILEKNTGNTLFELGHSNFLQD-TST
L1-1A2_Sar                    RVEYSDTHPQIYDHLIFDKGARNVKWSKESMFNKLCWQNWTATCKKMGLDLHLSPCTKVRSKWIKDLNIRPESLRYIEDKVGKTLHDIEAKGIFKA-DTP
L1-1_ET                       RIESPRIKPSAYRQLIFDKGPKTIKWEADALFNKWCWKQWISTCRKMKQDVYLTPCTRISSRWITDLEVKPQTIRTIKEGIGTNLRALAQGTHRLTAIGK
L1-1_AMe                      RIENPEMDPRLFGQLIFDKAGKNIRWKKDSLFNKWCWENWTATCKRMKLDHSLTPYTKINSKWMKDLNVRQESIKILEENIGNNFYDIGQSNLFHD-TSP
Meug                          RLGTQDAVGKEYSNLLFDKPKDPSFWDKNSLFDKNCWENWITVWRKLGIDPYLTPYTRIKSKWVHDLGIKIDTMNKLEKQGIVYLSDLWRREEFFT-KGE
Fcat                          RIETPELDPQTYGQLIFDKAGKNIQWKKDSLFNKWCWENWTATCRRLKLDHFLTPFTKINSKWIKDLNVRQETIKTLEEKAGKDLSDLSRSNLLLD-TSP
Mmul                          RTESSEIIPHIYSHLIFDKPERNKKWGKDSLFNKWCWENWLAISRKLKLDPFLTPYTKINSRWIRDLNVRPNTIKILEENLGSTIQDIGMGKDFMS-KTP
Pham                          RTESSEIIPHIYSHLIFDKPERNKKWGKDSLFNKWCWENWLAISRKLKLDPFLTPYTKINSRWIRDLNVRPNTIKILEENLGSTIQDIGMGKDFMS-KTP
```

```
                              ---------+---------+---------+---------+---------+---------+---------+---------+---------+---------+
                                     1010      1020      1030      1040      1050      1060      1070      1080      1090      1100
                              ---------+---------+---------+---------+---------+---------+---------+---------+---------+---------+
Conserved sites               XXXXXXXXXXXXXXXXXXXXXXXXXXXXXXXXXXXWEXXXXXXXXDXXLXXXXXXXXXXXXXXXXXXXXXXXXXXXWXXXXXXXXXXXXXXXXXXXXXX
lineage 1(L1-2_PVa)           KAREVKAKINEWDYIKLKSFCSAKETVNKVKRQPSEWENIFASNASDKGLISKIYKELIRLNNNKKTND-----PIKKWAEDLNRHFSLEDIQMANKYMK
lineage 2(L1-1_PVa)           RARETKEKINKWDYIKLKSFCTAKETINKTKRQPTVWEKIFANDTSDKGLISKIYKELIQLN-NKKTNN-----PIKKWAEDLNRYFSKEEIQMANRHMK
L1-BT                         RIMEIKAKINKWDLINLKSFCTSKETISKVKRQPSEWEKIIANEATDKQLISKIYKRLLQLN-SRKIND-----PIKKWAKELNRHFSKKDIQMANKHMK
L1-1_LA                       ---NDEEKPDNWELLKIKHLCSSKDFTKRVKRPPTDWERIFSYDISDQRLISKIYMILSKLN-HKKTNN-----PIKKWAKDMNTHFTKEDIQAANRYMR
L1-1_Vpa                      ---EIKARINKWDLMKLTSFCRAKETRNKTRRKPTEWEKIFASET-DKGLISKIYKQLIRLN-KKKINN-----PIQKWAEDLNKQFSKEDIQMIKKHMK
L1-1_DN                       KARAAKELIDKWDFLKIKAFCTSKEFVKKVKREPTQWEKIFGNHISDKKLITCIYKELLYLE-NKKINN-----PFKKWEKDLNRHFSKEEIQMAKKHMK
L1-1_Cpo                      TAQEILPRINNWDHFLLKSFCMSKEISSIVKRKPTNWEKILVNSLSDKGLLSKTYKELKKLR-PPKFKD-----PIQKWASEMNTHFSDEEMQMANKYMK
L1-1_SSc                      RVLTIKRKINKWDLIKLQSFCTAKETLNNTKRQPTEWEKIFASESTDKGLISKIYKXLLQLH-TKKTNN-----PIKKWAEDLNRQFSKEDIQMAEKHMK
L1-1B_Cho                     KAQATKEKIDKWELLKLRSFCTSKEFVKKVKRQPTQWEKIFGNHVSDKRLISCIYKEILQLN-DNSTDS-----PIIKWAKDMKRQFSEGEIQMAKKHMK
L1-2_EC                       QRRETIERINKWDFIRLKSFFKANENRIETKKQPTNWEKIFASHISDKGLISLIYKELSQLN-HKTSNN-----PIKKWAGDMNRHFSKEDILMANRHMK
L1MAB2_ML                     RAVETKEKMNKWDYIKIKSFCTAKETINKTTRKPTSWENIFANVISDKGLISKIYRELIQLN-KKKINN-----PIKKWAKDLNRHLSKEDIQKAKRHMK
L1-1_Str                      VAQELITRINKWDLLKLKSFFSARETVKEVNREPTSWEQIFTPHTSDRALISRMYKELKKLN-NKITNN-----PINKWAKDLNRHFSEEDIQSINKYMR
L1-1_MD                       IERITKCKINNFDYIKLKSFCTNKTNITKIRRETTNWEKIFIETS-DKGLITHIYNELNQLY-KKSSHS-----PIDKWAREMDRQFSDKEIKTINKHMK
L1-1_TS                       NPQDLREKIDKWDLIKLTSFCKAKETIKRAGRQPTDWEKVFANSRSDKGLTSWIYKELKRAE-KKKTNN-----PIIKWAKDMNRHFTKEDIRAANKHMK
L1-1_OP                       NAVEIKTKINNWDLIKLRSFCTARETINKVKRQPTEWEKIFAHDIGDRGLISRIYKELQNNQ-NVKTNK-----PLKKWAREMGKHFTKEQTQMANKHMK
L1_RN                         MAYALRSRIDKWDLIKLQSFCKAKDTVVRTKRQPTDWEKIFTNPTTDRGLISKIYKELKKLD-RRETNN-----PIKKWGSELNKEFTAEECRMAEKHLK
L1A_Mim                       KAITAATKINKWDMIKLQSFCTAKEIVMKVNRQPTEWEKIFASYASDKGLITRIYLELTKIR-KKKSNN-----PIKKWAKDLNRNFSKEDRRMANKHMK
L1-1_Cja                      KALATKAKIDKWDLIKLHSFCTAKETVIRVNRQPTEWEKIFAVYPSDKGLISRIYKELKQIY-KKKTNK-----PIQKWAKDMNRHFTKEDIHEANKHMK
L1-1_EE                       ITRKTKASINLWDYIKLKSFFTAKETTTQIKRPLTEWEKIFTCHTSDKSLITNIYKELARLN-NKTTNN-----PIQKWGEDLDRIFTTEEIQKAEKHMK
L1-1_Tbel                     KAQAIIPKIDKWDYIRLRSFCTAKDTVTSVNRQPSEWENIFVKYASDKGLITRIHRELKCLM-RKKMIN-----PIKRWESELNKSLSKEDIRTAKKHMR
L1-1_Pca                      KPQSTEEKIDKWELLRIKHFCSSKDFINRIKRTPTEWEKIFGNSMSDRRLISIIYKILQNIN-KKKINH-----PIKRWAKEMDRHFTKEEIQAAKKHMR
L1A_OC                        EAQAVKAKINYWDCIKLRSFCTAKETVRRVKRQPTEWEKIFANYATDKGLITRIYKEIKKLH-KNKTNN-----PLKRWAKDLNRHFSKEEIQMANRHMK
L1HS                          KAMATKAKIDKWDLIKLKSFCTAKETTIRVNRQPTTWEKIFATYSSDKGLISRIYNELKQIY-KKKTNN-----PIKKWAKDMNRHFSKEDIYAAKKHMK
L1-2_Dor                      ETQQIKERLDKWDCIKLQSFCRANDIASKINRKPTDWEKIFTSHTTDKGLISKIYLELKKLN-PPTTNPQRNNCPINKWAKDLKRNFSEEERRMANRHMK
L1-Y_CF                       KAKETKAKMNYWDFIKIRSFCTAKDTVNKTQRQPTEWEKIFANDISDKGLVSKIYKELIKLN-TKETNN-----PIMKWAKDMNRNLTEEDIDMANMHMR
L1-1A2_Sar                    LAKQVKTEINKWDYLKLRSFCTSRETVTKIQRQSTEWERIFTQYPSDKGLITRIYNALVELH-KKKTAN-----PIKKWGDEMNRNFPKEEIRMAERHMR
L1-1_ET                       GTHAGELEIDKWDLIRIKHLCTSKDFTKRVTRQPTDWERIFSNDTSDKGLITKIYNTLMACK-KRKTVN-----PLRKWAKELKRAFTREETXMANKHMR
L1-1_AMe                      KAREIKDKMNLWDFIRIKSFCTAKETVKKTKRQPTEWENIFAKDTTDKGLVSKIYKELLKLN-TRETNK-----QIIKWAEDMNRHFSNEDIQMANRHMK
Meug                          IECIMKCKMDNFDYIKLRSFCTTKPNATKIRRDVVNWERIFTAKLGDKGLISRIYRELTQMY-NHTSHS-----PIDKWSKDMNRQFSEEEIKAIYNHMK
Fcat                          KARELKAKVNYWDLMKIKSFCTAKETTNKTKRQPTEWEKIFANDISDKGLVSKIYKELTKLH-TRKTNN-----PVKKWAENMNRHFSKEDIRMANRHMK
Mmul                          KATAAKAKIDKWDLIKLKSFCTAKETTIRVNRQPTEWEKIFAIYSSDKGLISRTYKELKQIY-KKKTNN-----PIKKWAKDMNRHFSKEDIHTANRHMK
Pham                          KATAAKAKIDKWDLIKLKSFCTAKETTIRVNRQPTEWEKIFAIYSSDKGLISRTYKELKQIY-KKKTNN-----PIKKWAKDMNRHFSKEDIHTANRHMK
```

```
                              ---------+---------+---------+---------+---------+---------+---------+---------+---------+---------+
                                      1110      1120      1130      1140      1150      1160      1170      1180      1190      1200
                              ---------+---------+---------+---------+---------+---------+---------+---------+---------+---------+
Conserved sites               XCSXSLXXXEXQIKTXXRXHXTXXRXXXXXXXXXXXXCWXGCXXXGXLXHCWWXCXXXXPXWXXXWXXXXXXXXXXXPXXPXIXLLGXXXXXXXXXXXXX
lineage 1(L1-2_PVa)           RCSTSLAIREMQIKTTMRYHLTPVRIAIINRTSNNKCWRGCGEKGTLIHCWWDCKLVQPLWKTVWRFLK-KLRIDLPYDPAIPLLGIYPKDLKTHIRKDI
lineage 2(L1-1_PVa)           RCSRSLIIREMQIKTTMRYHLTPVRMAIINKSKN-KCWRGCGEKGTLIHCWWDCKLVQPLWKTVWRFLK-KLKMDIPFDPAIALLGIYPKKTKTIIRKDL
L1-BT                         RCSTSLIIREMQIKTTMRYHFTPVRMAAIQKSTNNKCWRGCGEKGTLLHCWWECKLVQPLWRTVWRFLK-KLEIEPPYDPAIPLLGIHTEK--TRRERDT
L1-1_LA                       KCSRSLAIREMQIKTTMRFHLTPTRLALIQKTQNNKCWRGCGEIGTLIHCWWECKMVQPLWKSIWRYLK-QLEIELPYNPEIPLLGIYPRETRAFTQTDI
L1-1_Vpa                      KCSISLIIREMQIKTTMRYHLTPVRMAVIQKSXNDKCWRGCGERGTLLHCWWECSLVQPLWKTVWRFLK-RLGIDLPYDPGIPLLGLYPEG--NLLQDDT
L1-1_DN                       KCSKSLAIREMQIKTTMRYHLTPIRLAAMKKTEEYKCWRGCEERGTLIHCWWECRRIQPFWRTVWRFLK-KLAIDLPYDPAIPLLGIYPAELKTRTQTDI
L1-1_Cpo                      KCSSSLVIREMQIKTTLRYHLTPERMARIKKTNNNKCWRGCGEKGTLLHCWWECRLVQPLWRSVWRFLK-KLGLEVPFIPAIPLLGIFPEELKASYHSDI
L1-1_SSc                      RCSASLIIREMQIKTTMRYHLTPARMAIIQKSTNNKCWRGCGEKGTLVHCWWDCKLVQPLWKAVWRFLR-KLNIELPFDPAIPLLGIYPEK--TTTRKDT
L1-1B_Cho                     KCSASLAIREMQIKTTMRYHLTPIRMAAIKQTGNYKCWRGCGEIGTLIHCWWDCIMVQPLWKTVWQFLR-KLDIELPFDPAIALLGIYPEDLKAVTRTDI
L1-2_EC                       RCSSSLIIREMQIKTTLRYHLTPVRMTKISKTNSNKCWRGCGEKGTLIHCWWECKLVQPLWKTVWRFLK-KLKIELPYDPAIPLLGIYPKSLKSAIPKVL
L1MAB2_ML                     TCSKSLIIREMQIKTTMRYHLTPVRMAIINKSTNDKCWRGCGEKGTLVHCWWECRLVQPLWKAVWRFLK-KLEMELPFDPVIPLLGIYPKKPETPIRKDI
L1-1_Str                      KCSPSLAIREMQIKTTLRYHLTPVRLAAIMKSNNNKCWRGCGEKGTLVHCWWDCKMVRPIWKAVWRFLG-KLGMDPPFDPAIALLGLFPEDLKRAHYRDT
L1-1_MD                       KCSTSLIIREMQIKTTLRYHLTPSRLANITAKESNECWRGCGKVGTLIHCWWSCELIQPFWRAIWNYAQRATKEYLPFDPAIALLGLYPKEIMDT---KT
L1-1_TS                       KCSTSLIIREMQIKTTLRYHLTPVRMAIINNSKNNSCWRGCGEKGTLLHCWWECKLVQPLWKAVWRFLK-ALKIDLPYDPAIPLLGIYPEEHKSLYKKDT
L1-1_OP                       KCSSSLAIREIQIKTSMRYHLTPVRLAHMNKSTNNTCWRGCGEKGSLLHCWWGCRLVQPLWKSVWRIFK-QLKFNIPYDPAIALLGIYPEH--LFYEKPT
L1_RN                         KCSTSLVIREMQIKTTLRFHLTPVRMAKIKNSGDSRCWRGCGERGTLLHCWWDCRLVKPFWKSVWRFLR-KLDIELPEDPAIPLLGIYP-KDASTYKRDT
L1A_Mim                       KCSTSLIIREMQIKTTMRYHLTPVRMAFIKKSPNNKCWRGCGERGTLLHCWWDCKLVQPLWKAIWRYLK-AIQVNLPFDPAIPLLGIYPNDPVTLYKKDT
L1-1_Cja                      KCSSSLVIREMQIKTTLRYHLTPVRMAIIKKSGDNRCWRGCGEIGTLLHCWWECKLVQPLWKTVWRFLK-DLEIEIPFDPAIPLLGIYPKDYKSFYYKDT
L1-1_EE                       KCSKSLIVREMQIKTTMRYHFTPVRMSHIRKGNSSKCWRGCGVKGTLLHCWWECQLVQPLWRTVWRTLR-RLEMDLPYDPAIPLLGIYPKEPNTSIQKDL
L1-1_Tbel                     KCSTSLIIREMQIKTTMRYHLTPVRMAKIKNIKSDKCWRGCGEKGTLLHCWWECKLVQPLWRTIWRFLK-DLQIELPFDPAIPLLGIYPKDRRTLSQKSA
L1-1_Pca                      TCSRSLVIKEIQIKTTMRYHLTPTRLAMIQKTQNNKCWRGCGESGTLLHCWWGCKMVQPLWKAIWRFLK-KMDIDIPFDPVIPLLGIYPTESRPFTPTDT
L1A_OC                        KCSRSLAIREMQIKTTMRFHLTPVRMAHXQKSTNNRCWXGCGEKGTLTHCWWECKLVKPLWKSVWRFLR-NLNITLPFDPAIPLLGIYPKEFKLANKKAV
L1HS                          KCSSSLAIREMQIKTTMRYHLTPVRMAIIKKSGNNRCWRGCGEIGTLLHCWWDCKLVQPLWKSVWRFLR-DLELEIPFDPAIPLLGIYPKDYKSCCYKDT
L1-2_Dor                      KCSTSLAIKEMQIKTTLRYHLTPVRTAKIKNSNNNRCWRGCGQKGTLLHCWWECKLVQPLWKAVWRFLK-RLHIELPYDPAIPLLGIYPNVYKQDHTKAT
L1-Y_CF                       KCSASLAIREIQIKTTMRYHLTPVRMGKINKAGNNKCWRGCGEKGTLLHCWWECELVQPLWKTVWRFLK-QLKIYLPYDPAIALLGIYPKDTNAMKRRDT
L1-1A2_Sar                    KCSTSLIIREMQIKTTMRYHLTPQRLAHIPKNKSNRCWRGCGEKGTLLHCWWECRLVQPFWKTIWTILK-KLEIELPFDPAIPLLGIYPGEAKRYSRDGI
L1-1_ET                       KCSRSLAIREMQIKTTMRYHLTPLRLAQISKSESNKCWRGCGEVGTLLHCWWSCRFVQPLWKAIWRYLK-KMEIDLPYDPAIPLLGIYPVEATNKTRPVI
L1-1_AMe                      KCSKSLAIREIQIKTTLRYHLTPVRMAKIDKARNNNCWRGCGERGSLLHCWWECKLVQPLWKTVWRSLK-KLKIELPYDPAIALLGVYPKDTDVVKRRAI
Meug                          KCSKSLLVREMQIKTTLRYHITLXRLANMTEQENDKCWRGCGKVGTLIHCWWSCERIQPFWRAIWNYAQRATKMCIPFDPAISLLGLYPQEIIKMGKGPT
Fcat                          RCSASLLIREIQIKTTLRYHLTPVRVAKMNKSGDYRCWRGCGETGTLLHCWWECKLVQPLWKAVWRFLR-KLKIDLPYDPAIALLGIYPRDTGVLMHRAT
Mmul                          KCSSSLAIREMQIKTTMRYHLTPVRMAIIKKSGNNRCWRGCGEIGTLLHCWWDCKLVQPLWKTVWRFLK-DLELDVPYDPAIPLLGIYPKDYKLCCYKDT
Pham                          KCSSSLAIREMQIKTTMRYHLTPVRMAIIKKSGNNRCWRGCGEIGTLLHCWWDCKLVQPLWKTVWRFLK-DLELDVPYDPAIPLLGIYPKDYKLCCYKDT
```

```
                              ---------+---------+---------+---------+---------+---------+---------+---------+-----
                                      1210      1220      1230      1240      1250      1260      1270      1280
                              ---------+---------+---------+---------+---------+---------+---------+---------+-----
Conserved sites               XXXXXXAAXXXXAXXWXXXXXPXXXXWXXKXWXXXXXEXYXXXXXXXXXXXXXXXWXXXXXXXXXXXXXXXXXXXXXXXXXXXXX
lineage 1(L1-2_PVa)           CTPMFIAALFTVARTWKQPKCPTIDDWLKKLWYIYTMEYYSAIRRD-EILPFATTWMDLEIIVLSEISQTEKVENHMISLICGI.
lineage 2(L1-1_PVa)           CTPIFMAAQFTIAKIWKQPKCPSTDDWIKKLWFIYTMEYYSAIKKN-EILPFATTWMDLEVIMLSEISQTEKDKYHMISLICGI.
L1-BT                         CTPMFIAALFIIAKTWKQPRCPSADEWIRKLWYIYTMEYYSAIKKN-TFESVLMRWMKLEPIIQSEVSQKEKHKYSILTHIYGI.
L1-1_LA                       CTPMFIAALFTIAKSWKQPRCPSTDEWVNKLWYIHTMEYYASIKNSDESVKHFITWRNLEGIMLSEISQRQKDKYCIRPLL.
L1-1_Vpa                      CTPMFIAALFTIAKTWKQPKCPSTGDWIKKMWYIYTMEYYSAIKTD-NITPFAATWMLLENVILSEVSQKEKEKYHMRSLICGI.
L1-1_DN                       CTPMFIAALFTIAKSWNQPKCPSTDEWINKMWYIHTMEYYSAVRTNT-LQTHVITWMNLENLMLSEATQALKDKYYMTSMI.
L1-1_Cpo                      CAPMFIAAQFVIARSWKQPKCPSTEEWIKKLWYFYTMEYYSAIKKD-HIEIFINKWAQLETILISEINQSRMCEYRIVSLM.
L1-1_SSc                      CTPMFIAALFTIAKTWKQPKCPSTEEWIQKMWYIYTMEYYSAIKKN-EIPAFLATWMDLETIMLSEVSHTMRHQHQMLSLTCGI.
L1-1B_Cho                     CTPMFIAALFTIAKRWKQPKCPSTDEWINKMWYIHTMEYYAAVRRND-VVKHMTTWMNLEDIMLSEISQAQKEKYYMLPLM.
L1-2_EC                       CTPMFIAALFTIAKTWKQPKCPSTDEWIKKMWYIYTMEYYSAAKQN-KIIPFAITWMDLERIMLSEISQREKDNLCMTPLI.
L1MAB2_ML                     CTQMFIAAQFTIAKIWKQPKCPSVDEWIRKLWYIYTMEYYAAVKKK-ELLPFATAWMELESIMLSETSQSMKEKYHMISLFHG.
L1-1_Str                      ATSMIIAAQFTIARLWNQPRCPSIDEWIKKMWHLYTMEYYAALRND-KIIEFAGKWMALEQIMLSEASQALKNKCQMSSLI.
L1-1_MD                       CTKIFIAALFVVAQNWKTRGCPSIGEWLNKLWYMLVMEYYCAQRNN-KVEKFHGDWNNLQEVMQSERSRTRRTLYTETNTLWYNR
L1-1_TS                       CTRMFIAALFTIARTWKQPCCPSKEDWIKKMWYIYTMEYYAAIKKN-KIMNFAATWMELESIILSDLSQKQRSEYHMFSLI.
L1-1_OP                       CTPMLIAAQSVIAKTWKQPKCPSTEDWIRKLWFIYSMEYYSAIKKN-KMQFFVAKWAKLETIMLREMSQSQKVKYHMFALI.
L1_RN                         CSTMFIAALFIIARSWKEPRCPSTEEWIQKMWYIYTMEYYSAIKNN-EFMKFVGKWLELENIILSELTQSQKDIHGMHSLISGY.
L1A_Mim                       CTRMFIAAQFIIARLWKQPKCPSIQEWINKMWYMYTMEYYSALRNNGDIAHLIFSWLELEPILLSEVSQEWKNKHQIYSPANWY.
L1-1_Cja                      CTRMFIAALFTIAKTWNQPKCPSMIDWTGKMWHIYTMEYYAAIKND-EFVSFVGTWMNLENIILSKLTQEQKMKYRMFSLIGGC.
L1-1_EE                       CTHMFLAAQFVIAKTWKQPRCPTTDEWLSKLWYIYTMEYYSAVKNG-DFTVFSRSWMDLEKIMLSEISQKQKDEYGMISLSGRS.
L1-1_Tbel                     CTSMFIAALFTIAKTWNQPKCPQTDEWIGKVWYIYSMEYYSAIKNN-KTLEFDRKWSDXEDLLLSEVSQAMKERYCMYPLNIYRL
L1-1_Pca                      CTRMFIAAQSTIAKRWNQPRCPTTEEWIGKLWYIYSMEHYAMIKNNDDSRKHLLTWFQLEDIMLSESSQRQKDKYCMRPLR.
L1A_OC                        CTLMFIAAQFTIAKTWNQPKCPSTVDWIKKLWDMYSLEYYTAVRNN-EIQSFATKWRNLEHIMLSEXSQSQRDKYHMFSLIGDN.
L1HS                          CTRMFIAALFTIAKTWNQPKCPTMIDWIKKMWHIYTMEYYAAIKND-EFISFVGTWMKLETIILSKLSQEQKTKHRIFSLIGGN.
L1-2_Dor                      STTMFIAALFTIAKIWNQPRCPSVDEWIKKMWYIYTMEFYASIRKN-DIAPFVRKWKNLEKIILSEVSQAQRNIGPMVSLICNI.
L1-Y_CF                       CTPMFLAAMATIAKLWKEPRCPTKDEWIKKMWFMYTMEYYSAIRND-KYPPFASTWMELEGIMLSEVSQSEKDKHYMFSFIWGI.
L1-1A2_Sar                    CISMFIAALFTIATIWKKPECPKTDDWLKKLWYIYTMEYYVAVRKH-EVMKFAYKWINMESIMLSEMSQKERDRHRKIALICGI.
L1-1_ET                       CAPMFIAAQFTIAKTWKQPKFPSIDEWISKLWHIHTMEYYAALKSADDRMKHVASWEELEGIMLSEVSQXQKDRYNMSPLR.
L1-1_AMe                      CTPMFIAALSTIAKSWKEPRCPSTDDWIKKLWSIYTMEYYSAIRKN-EFSTFAATWTALEEIMLSEISQAEKDNYHMISLIYGT.
Meug                          CTKIFIAALYVVAKNWKSRGCPSIGEWLNKLWYMNVMEYYCAIRND-EQEDFREAWKDLYDLMLSERSRTRRTLCTATTTVCESF
Fcat                          CTPMFIAALSTIAKLWKEPKCPSTDEWIKKLWFIYTMEYYVAMRKN-EIWPFVATWMELESVMLSEISHTEKDRYHMVSLLCGS.
Mmul                          CTRMFIAALFTIAKTWNQPKCPSVTDWIKKMWHIYTMEYYAAIKKD-EFVSFVGTWMQLETIILSKLSQEQKTKHRMFSLIGGN.
Pham                          CTRMFIAALFTIAKTWNQPKCPSVTDWIKKMWHIYTMEYYAAIKKD-EFASFVGTWMQLETIILSKLSQEQKTKHRMFSLIGGN.
```
